# New Polyketides and New Benzoic Acid Derivatives from the Marine Sponge-Associated Fungus *Neosartorya quadricincta* KUFA 0081

**DOI:** 10.3390/md14070134

**Published:** 2016-07-16

**Authors:** Chadaporn Prompanya, Tida Dethoup, Luís Gales, Michael Lee, José A. C. Pereira, Artur M. S. Silva, Madalena M. M. Pinto, Anake Kijjoa

**Affiliations:** 1 ICBAS—Instituto de Ciências Biomédicas Abel Salazar, Universidade do Porto, Rua de Jorge Viterbo Ferreira, 228, 4050-313 Porto, Portugal; chadaporn@buu.ac.th (C.P.); lgales@ibmc.up.pt (L.G.); jpereira@icbas.up.pt (J.A.P.); 2 Interdisciplinary Centre of Marine and Environmental Research (CIIMAR), Rua dos Bragas 289, 4050-313 Porto, Portugal; madalena@ff.up.pt; 3 Department of Plant Pathology, Faculty of Agriculture, Kasetsart University, 10900 Bangkok, Thailand; agrtdd@ku.ac.th; 4 Instituto de Biologia Molecular e Celular (IBMC), Universidade do Porto, Rua de Jorge Viterbo Ferreira, 228, 4050-313 Porto, Portugal; 5 Department of Chemistry, University of Leicester, University Road, Leicester LE 7 RH, UK; ml34@leicester.ac.uk; 6 Departamento de Química & QOPNA, Universidade de Aveiro, 3810-193 Aveiro, Portugal; artur.silva@ua.pt; 7 Laboratório de Química Orgânica, Departamento de Ciências Químicas, Faculdade de Farmácia Universidade do Porto, Rua de Jorge Viterbo Ferreira, 228, 4050-313 Porto, Portugal

**Keywords:** *Neosartorya quadricincta* KUFA 0081, marine-derived fungus, polyketides, pentaketides, benzoic acid derivatives, *Clathria reinwardti*

## Abstract

Two new pentaketides, including a new benzofuran-1-one derivative (**1**) and a new isochromen-1-one (**5**), and seven new benzoic acid derivatives, including two new benzopyran derivatives (**2a**, **b**), a new benzoxepine derivative (**3**), two new chromen-4-one derivatives (**4b**, **7**) and two new benzofuran derivatives (**6a**, **b**), were isolated, together with the previously reported 2,3-dihydro-6-hydroxy-2,2-dimethyl-4*H*-1-benzopyran-4-one (**4a**), from the culture of the marine sponge-associated fungus *Neosartorya quadricincta* KUFA 0081. The structures of the new compounds were established based on 1D and 2D NMR spectral analysis, and in the case of compounds **1**, **2a**, **4b**, **5**, **6a** and **7**, the absolute configurations of their stereogenic carbons were determined by an X-ray crystallographic analysis. None of the isolated compounds were active in the tests for antibacterial activity against Gram-positive and Gram-negative bacteria, as well as multidrug-resistant isolates from the environment (MIC > 256 μg/mL), antifungal activity against yeast (*Candida albicans* ATTC 10231), filamentous fungus (*Aspergillus fumigatus* ATTC 46645) and dermatophyte (*Trichophyton rubrum* FF5) (MIC > 512 µg/mL) and in vitro growth inhibitory activity against the MCF-7 (breast adenocarcinoma), NCI-H460 (non-small cell lung cancer) and A375-C5 (melanoma) cell lines (GI_50_ > 150 µM) by the protein binding dye SRB method.

## 1. Introduction

**Figure 1:**
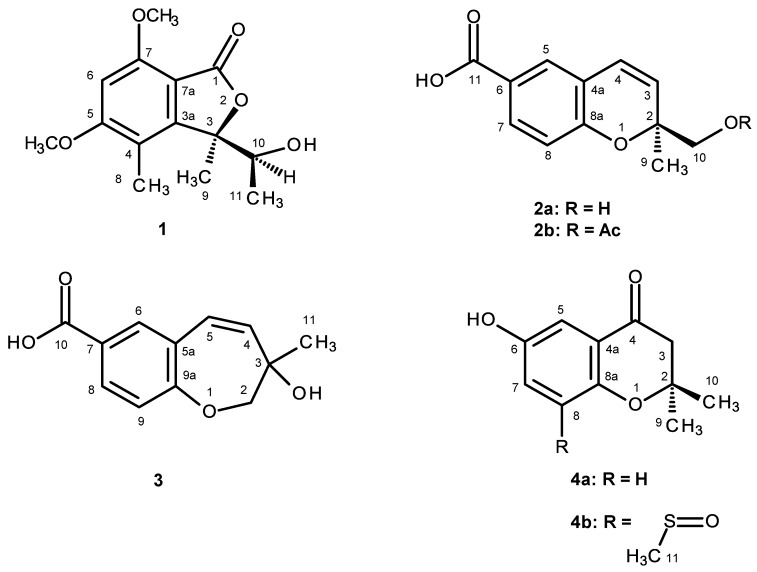

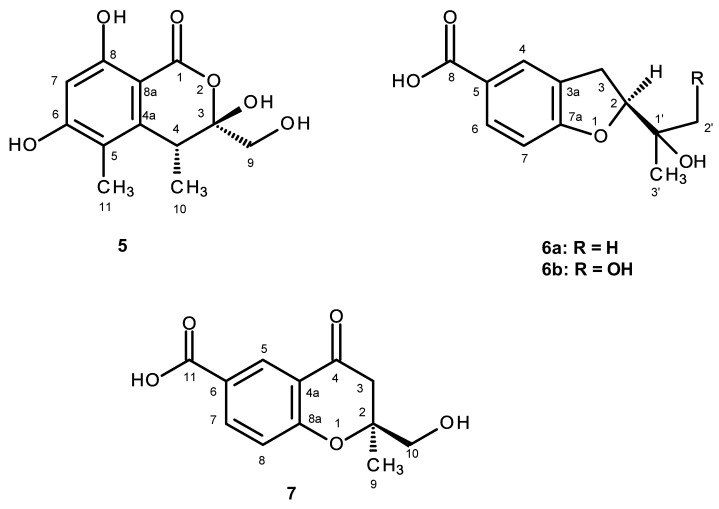
Secondary metabolites of *N. quadricincta* KUFA0081.

*Aspergillus* section *Fumigati* and its teleomorph *Neosartorya* include many important species because they can be pathogenic or allergenic to man, as well as causing food spoilage and producing mycotoxins. Certain species are also found to produce interesting bioactive secondary metabolites that can be considered to have potential for drug development [1]. For this reason, we have investigated the bioactive secondary metabolites produced from the cultures of four *Neosartorya* species collected from soil in Thailand, i.e., *Neosartorya glabra* KUFC 6311 [2], *N. pseudofischeri* KUFC 6422 [3], *N. siamensis* KUFC 6349 [4] and *N. fischeri* KUFC 6344 [5], as well as six marine-derived species of *Neosartorya*, including *N. paulistensis* KUFC 7898 [6], *N. laciniosa* KUFC 7896 [5], *N. spinosa* KUFC 8104, *N. tsunodae* KUFC 9213 [5], *N. siamensis* KUFA 0017 and *N. takakii* KUFC 7898 [7], as well as one marine-derived *Aspergillus* species (*Aspergillus similanensis* KUFA 0013) [8,9]. Recently, we have also reported the antifungal activity of the crude extract of *N. quadricincta* KUFA 0064, isolated from an agricultural soil in Southern Thailand, against plant pathogenic fungi, which are causative agents of diseases of economically-important plants of Thailand [10]. So far, the only report on secondary metabolites of *N. quadricincta* was by Ozoe et al., who described the isolation of dihydroisocoumarin derivative, PF1223, from the culture of *N. quadricincta* strain PF1223 (unidentified source). This compound was shown to inhibit the [^3^H] EBOB binding by 65% [11]. Thus, in our ongoing search for bioactive secondary metabolites from marine-derived fungi from Thai waters, we have investigated the culture of *N. quadricincta* KUFA 0081, isolated from the marine sponge *Clathria reinwardti*, which was collected from the Coral reef at Samae San Island in the Gulf of Thailand. The ethyl acetate extract of the culture of this fungus yielded, besides the previously described 2,3-dihydro-6-hydroxy-2,2-dimethyl-4*H*-1-benzopyran-4-one (**4a**) [12], two new polyketide derivatives (**1**, **5**) and seven new benzoic acid derivatives (**2a**, **2b**, **3**, **4b**, **6a**, **6b** and **7**) (Figure 1). All of the isolated compounds were tested for their antibacterial activity against Gram-positive and Gram-negative bacteria, as well as multidrug-resistant isolates from the environment and for their antifungal activity against yeast (*Candida albicans* ATCC 10231), filamentous fungus (*Aspergillus fumigatus* ATCC 46645) and dermatophyte (*Trichophyton rubrum* FF5). Additionally, these compounds were also evaluated for their in vitro growth inhibitory activity against the MCF-7 (breast adenocarcinoma), NCI-H460 (non-small cell lung cancer) and A375-C5 (melanoma) cell lines by the protein binding dye SRB method.

## 2. Results and Discussion

**Figure 2:**
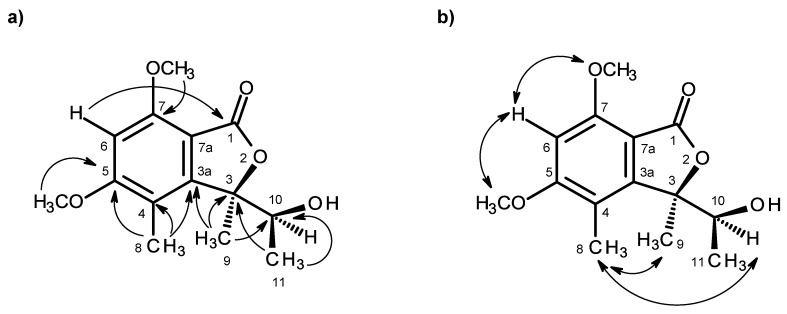
Key HMBC (**→**) (**a**) and NOESY (**↔**) (**b**) correlations for compound **1**.

**Figure 3:**
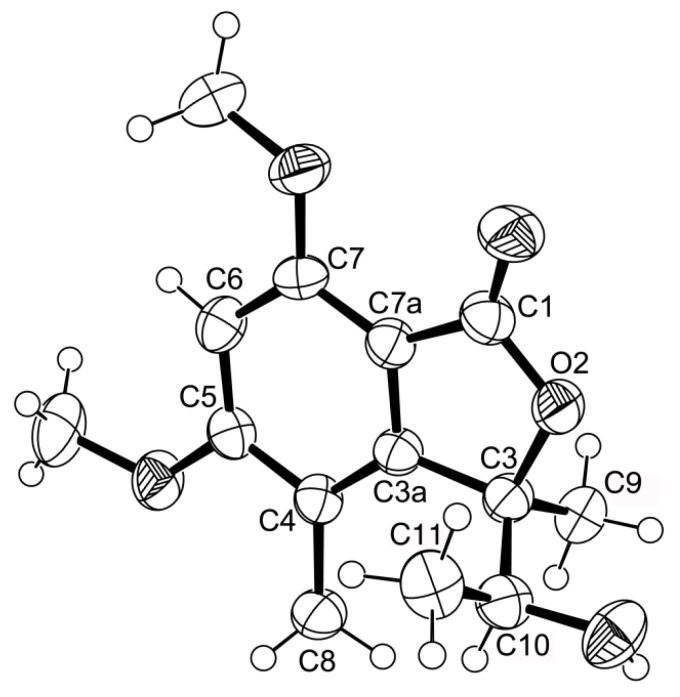
ORTEP diagram of compound **1**.

Compound **1** was isolated as white crystals (mp, 176–177 °C), and its molecular formula C_14_H_18_O_5_ was established on the basis of the (+)-HRESIMS *m/z* 267.1243 [M + H]^+^ (calculated 267.1332), indicating six degrees of unsaturation. The IR spectrum showed absorption bands for hydroxyl (3455 cm^−1^), conjugated ester carbonyl (1723 cm^−1^) and aromatic (1612, 1596 cm^−1^) groups. The ^13^C NMR, DEPT and HSQC spectra (Table 1, Supplementary Information, Figures S2 and S4) exhibited the signals of one conjugated ester carbonyl (δ_C_ 168.2), five quaternary sp^2^ (δ_C_ 164.6, 158.3, 152.8, 111.7, 105.5), one methine sp^2^ (δ_C_ 94.5), one oxygen bearing quaternary sp^3^ (δ_C_ 88.8), one oxygen bearing methine sp^3^ (δ_C_ 70.8), two methoxyl (δ_C_ 56.1 and 56.0) and three methyl (δ_C_ 21.5, 17.8 and 11.2) groups. The ^1^H NMR spectrum (Table 1, Supplementary Information, Figure S1) revealed the presence of, besides a singlet of one aromatic proton at δ_H_ 6.41, a quartet of the oxymethine proton at δ_H_ 4.22 (*J* = 6.4 Hz), two singlets of the methoxyl groups at δ_H_ 3.97 s and 3.92 s, two methyl singlets at δ_H_ 2.12 s and 1.76 s, a methyl doublet at δ_H_ 0.87 (*J* = 6.4 Hz) and a broad band of the hydroxyl proton at δ_H_ 2.15. The ^1^H and ^13^C data (Table 1) revealed the presence of a pentasubstituted benzene ring. That this pentasubstituted benzene ring was part of the 5,7-dimethoxy-3,4-dimethyl-2-benzofuran-1(3*H*)-one ring system was corroborated by the HMBC correlations (Table 1, Figure 2a, Supplementary Information, Figure S5) of H-6 (δ_H_ 6.41, s) to C-7a (δ_C_ 105.3), C-4 (δ_C_ 111.7), C-7 (δ_C_ 158.3), C-5 (δ_C_ 164.6) and C-1 (δ_C_ 168.2), of OMe-5 (δ_H_ 3.92, s) to C-5, of OMe-7 (δ_H_ 3.97, s) to C-7, of H_3_-8 (δ_H_ 2.12, s) to C-4, C-3a (δ_C_ 152.8) and C-5, of H_3_-9 (δ_H_ 1.76, s) to C-3a and of the NOESY correlations of H-6 to OMe-5 and OMe-7. That another substituent of C-3 was a 1-hydroxyethyl group was supported by the COSY correlations of H-10 (δ_H_ 4.22, q, *J* = 6.4 Hz) to H_3_-11 (δ_H_ 0.87, d, *J* = 6.4 Hz), by the HMBC correlations of H_3_-9 to C-10 (δ_C_ 70.8), C-3 (δ_C_ 88.8) and C-3a and of H_3_-11 (δ_H_ 0.87, d, *J* = 6.4 Hz) to C-3 and C-10 (Table 1, Figure 2a), as well as by the NOESY correlations of H_3_-8 to H-10, H_3_-9, of H_3_-11 to H-10, OH-10 and of H_3_-9 to H-10 (Table 1, Figure 2b, Supplementary Information, Figure S6). Final proof of the structure and the stereochemistry assigned to compound **1** was provided by its X-ray analysis (Figure 3), and since the diffraction data were collected with a Gemini PX Ultra equipped with CuKα radiation, it was possible to establish the absolute configurations of C-3 and C-10, respectively, as 3*R* and 10*S*. Since **1** is a new compound, we have named it quadricinctone A.

**Figure 4:**
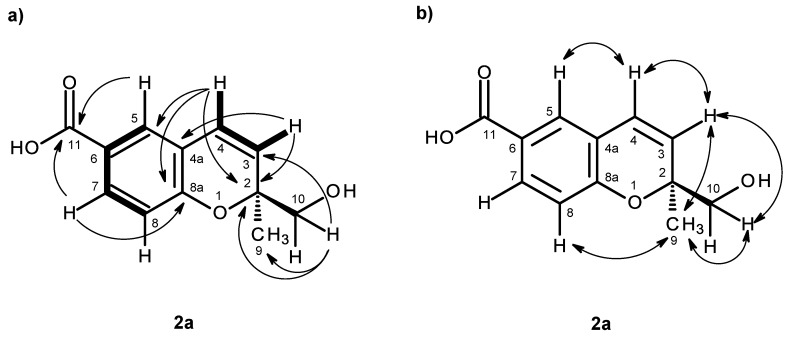
Key COSY (–), HMBC (→) (**a**) and NOESY (↔) (**b**) correlations for compound **2a**.

**Figure 5:**
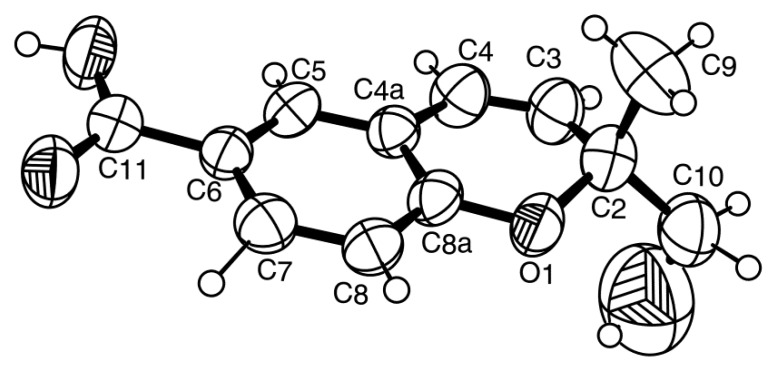
ORTEP diagram of compound **2a**.

Compound **2a** was also isolated as white crystals (mp. 147–148 °C), and its molecular formula C_12_H_12_O_4_ was determined based on the (+)-HRESIMS *m/z* 221.0820 [M + H]^+^ (calculated 221.0814), indicating seven degrees of unsaturation. The IR spectrum showed absorption bands for hydroxyl (3447 cm^−1^), a conjugated carbonyl (1696 cm^−1^), aromatic (1609 cm^−1^) and olefin (1647 cm^−1^) groups. The ^13^C NMR, DEPT and HSQC spectra (Table 2, Supplementary Information, Figures S8 and S10) exhibited the signals of one conjugated carboxyl carbonyl (δ_C_ 167.0), three quaternary sp^2^ (δ_C_ 156.8, 123.0, 120.5), five methine sp^2^ (δ_C_ 130.8, 128.6, 127.9, 122.8, 115.7), one oxy-quaternary sp^3^ (δ_C_ 80.5), one oxymethylene sp^3^ (δ_C_ 67.1) and one methyl (δ_C_ 23.3) groups. The ^1^H NMR spectrum (Table 2, Supplementary Information, Figures S7) revealed, besides the presence of three aromatic protons of the 1,2,4 trisubstituted benzene ring at δ_H_ 6.79, d (*J* = 8.4 Hz), 7.65, d (*J* = 2.1 Hz) and 7.69, dd (*J* = 8.4, 2.1 Hz), two doublets of the protons of a *cis*-double bond at δ_H_ 6.56, d (*J* = 10.0 Hz) and 5.74, d (*J* = 10.0 Hz), a methyl singlet at δ_H_ 1.31, a singlet of two protons at δ_H_ 3.46 and two broad signals of the hydroxyl protons at δ_H_ 12.59 and 5.07, respectively. The COSY spectrum (Table 2, Figure 4a, Supplementary Information, Figures S9) exhibited cross-peaks of H-4 (δ_H_ 6.56, d, *J* = 10.0 Hz) to H-3 (δ_H_ 5.74, d, *J* = 10.0 Hz), of H-7 (δ_H_ 7.69, dd, *J* = 8.4, 2.1 Hz) to H-5 (δ_H_ 7.65, d, *J* = 2.1 Hz) and H-8 (δ_H_ 6.79, d, *J* = 8.4 Hz), confirming the presence of the 1,2,4 trisubstituted benzene ring and the *cis*-double bond. That the 1,2,4-trisubsituted benzene ring and the *cis*-double bond were part of the 2*H*-chromene-6-carboxylic acid moiety was corroborated by the HMBC correlations (Figure 4a, Supplementary Information, Figures S11) of H-5 to C-4 (δ_C_ 122.8), C-7 (δ_C_ 130.8), C-8a (δ_C_ 156.8) and C-11 (δ_C_ 167.0), of H-7 to C-5 (δ_C_ 127.9), C-8a, C-11, of H-8 to C-4a (δ_C_ 120.5), C-6 (δ_C_ 123.0) and C-8a, as well as of H-3 to C-4a and of H-4 to C-4a, C-5 and C-8a. As the HMBC spectrum also exhibited correlations of the methyl singlet at δ_H_ 1.31 (H_3_-9) to C-3, C-2 (δ_C_ 80.5) and C-10 (δ_C_ 67.1) and of the singlet at δ_H_ 3.46 (H-10) to C-2, C-3 and CH_3_-9 (δ_C_ 23.3), the methyl and hydroxymethyl groups were placed on C-2. The NOESY correlations (Figure 4b, Supplementary Information, Figures S12) of H-4 to H-3 and H-5, of H-3 to H-4, H_3_-9 and H_2_-10, of H-8 to H-7, H_2_-10 and H_3_-9 and of H_3_-9 to H-8, H-10 also confirmed this hypothesis. Since compound **2a** was obtained in a suitable crystal, X-ray analysis was carried out, and the ORTEP view shown in Figure 5 revealed that the absolute configuration of C-2 is *S*. A literature search indicated that **2a** has never been previously reported; therefore, it was named quadricinctapyran A.

**Figure 6:**
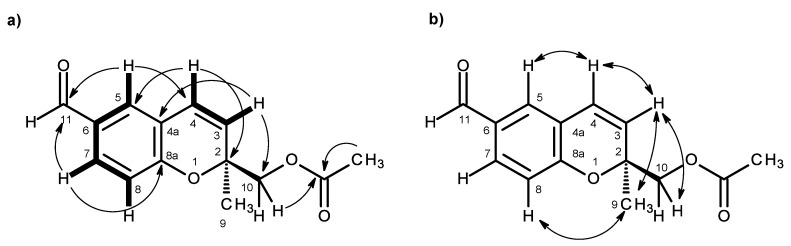
Key COSY (–), HMBC (→) (**a**) and NOESY (↔) (**b**) correlations for compound **2b**.

Compound **2b** was isolated as a white solid (mp. 118–119 °C), and its molecular formula C_14_H_14_O_5_ was established on the basis of the (+)-HRESIMS *m/z* 263.0971 [M + H]^+^ (calculated 263.0919), indicating eight degrees of unsaturation. The ^1^H and ^13^C NMR spectral features (Supplementary Information, Figures S13 and S14) of compound **2b** resembled those of compound **2a**, except for an additional carbonyl carbon at δ_C_ 170.8 and a methyl group at δ_C_ 20.7 (δ_H_ 1.98, s), characteristic of the acetoxyl group (Table 2). Moreover, since the signals of the oxymethylene protons (H_2_-10) of compound **2b** appeared as two doublets at δ_H_ 4.13 (*J* = 11.7 Hz) and 4.24 (*J* = 11.7 Hz), ca. 0.7 ppm higher than that of H_2_-10 in compound **2a**, it was clear that the acetoxyl group was on C-10. This was also corroborated by the HMBC correlations (Table 2, Figure 6a, Supplementary Information, Figure S17) of H_2_-10 and the methyl singlet at δ_H_ 1.98 to the carbonyl of the acetoxyl group (δ_C_ 170.8). Since Compound **2b** could not be obtained as a suitable crystal for X-ray analysis, the absolute configuration of its C-3 could not be determined with certainty. However, as compound **2b** is the acetate derivative of compound **2a**, it was speculated that the stereochemistry of its C-2 should be the same as that of C-2 of compound **2a**, i.e., 2*S*. In order to confirm this hypothesis, the NOESY experiments were carried out. The NOESY spectrum of compound **2b** showed a weak correlation of H-8 to H_3_-9 and not to H_2_-10 (Figure 6b, Supplementary Information, Figure S18), similar to what has been observed for compound **2a**. Acid hydrolysis of **2b** gave the product whose structure was confirmed as **2a** by ^1^H and ^13^C NMR data, as well as the optical rotation. Therefore, the absolute configuration of C-2 of **2b** is assigned as 2*S*. Compound **2b** is also a new compound, thus we named it quadricinctapyran B.

**Figure 7:**
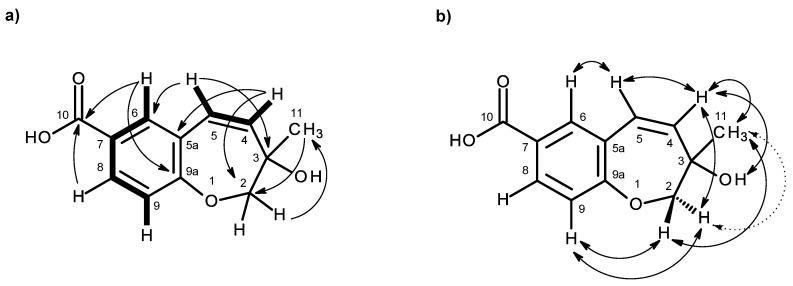
Key COSY (–), HMBC (→) (**a**) and NOESY (↔) (**b**) correlations for compound **3**.

The molecular formula C_12_H_12_O_4_ of compound **3**, a white solid (mp 189–191 °C), was established based on the (+)-HRESIMS *m/z* 221.0819 [M + H]^+^ (calculated 221.0814), and thus, it is an isomer of compound **2a**. Furthermore, the general features of its ^1^H and ^13^C NMR spectra resembled those of compound **2a**. However, the chemical shift values of some of the proton and carbon signals were slightly different from those observed in compound **2a**. The ^13^C NMR, DEPT and HSQC spectra (Table 3, Supplementary Information, Figures S20 and S22) exhibited the signals of one conjugated carboxyl carbonyl (δ_C_ 166.7), three quaternary sp^2^ (δ_C_ 161.2, 125.3, 125.0), five methine sp^2^ (δ_C_ 139.5, 134.6, 129.8, 123.5, 119.8), one oxy-quaternary sp^3^ (δ_C_ 70.7), one oxymethylene sp^3^ (δ_C_ 77.0) and one methyl (δ_C_ 26.1) groups. The ^1^H NMR spectrum (Table 3, Supplementary Information, Figure S19) revealed the existence of three aromatic protons of the 1,2,4-trisubstituted benzene ring, similar to that of compound **2a**, at δ_H_ 7.05, d (*J* = 8.4 Hz), 7.74, dd (*J* = 8.4, 2.1 Hz), 7.89, d (*J* = 2.1 Hz), two olefinic protons of the *cis*-double bond at δ_H_ 5.95, dd (*J* = 12.0, 1.2 Hz) and 6.31, d (*J* = 12.0 Hz), two oxymethylene protons at δ_H_ 3.84, d (*J* = 11.1 Hz) and 4.02, dd (*J* = 11.1, 1.6 Hz), and a methyl singlet at δ_H_ 1.26. That the carboxylic acid functionality was on C-7, and the substituent with a *cis*-double bond was on C-5a was substantiated by the HMBC correlations of H-6 (7.89, d, *J* = 2.1 Hz) to C-10 (δ_C_ 166.7), C-9a (δ_C_ 161.2), C-8 (δ_C_ 129.8) and C-5 (δ_C_ 123.5), of H-5 (δ_H_ 6.31, d, *J* = 12.0 Hz) to C-6 (δ_C_ 134.5) and C-9a and of H-4 (δ_H_ 5.95, dd, d, *J* = 12.0, 1.2 Hz) to C-5a (δ_C_ 125.0) (Table 3 and Figure 7a, Supplementary Information, Figure S23). However, contrary to compound **2a**, compound **3** showed the HMBC correlations of H_2_-2 (δ_H_ 3.84, d, *J* = 11.1 Hz and 4.02, dd, *J* = 11.1, 1.6 Hz) to not only C-4 (δ_C_ 139.5), but also to C-9a (Table 3 and Figure 7a). Consequently, the benzoic acid moiety was fused with the 2,3,6,7-tetrahydro-oxepine ring through C-5a and C-9a. That the methyl group and the hydroxyl group were on C-3 of the oxepin ring was confirmed by the HMBC correlations of the methyl singlet at δ_H_ 1.26 (H-11) to C-3 (δ_C_ 70.7), C-2 (δ_C_ 77.0) and C-4, as well as of H_2_-2 to C-11 (δ_C_ 26.1) and C-3 (Table 3 and Figure 7). This was also corroborated by the NOESY correlations of H_3_-11 to OH-3 (δ_H_ 3.39, br), H_2_-2 and H-4 (Table 3 and Figure 7b, Supplementary Information, Figure S24). Therefore, Compound **3** was identified as 3-hydroxy-3-methyl-2,3-dihydro-1-benzoxepine-7-carboxylic acid.

**Figure 8:**
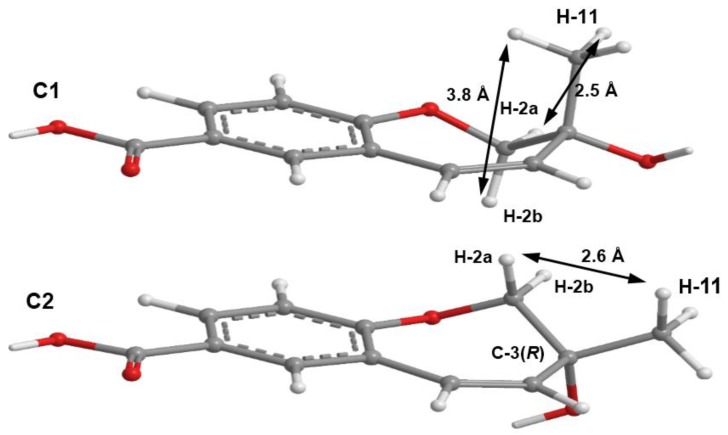
The two minimal energy conformations, C1 and C2, for the structure of **3**, with *R* configuration for C-3. All calculated distances and energies are exactly the same for the pairs 3*R*-C1/3*S*-C2 and 3*R*-C2/3*S*-C1. The shorter predicted inter-hydrogen distances H-2/H-11 are presented; for a discussion of the average, NOE effective distances, please refer to the main text.

As compound **3** could not be obtained as a suitable crystal for X-ray analysis, an effort to tentatively determine the relative configuration of the stereogenic carbon (C-3) by molecular mechanics conformation analysis and the NOESY experiments was carried out. Stochastic conformational search on the computational models of the structure of **3** with C-3 in *R* configuration, followed by energy minimization, converged to two half-chair conformations for the seven-membered ring C1 and C2, as depicted in Figure 8, regardless of the modelling level of the theory used (MP2/6-311G, PM3, MMFF and MM2). All methods, except for PM3, also agree that conformation C2, with the methyl group in the equatorial position, is more stable by ca. 2 kcal/mol. However, this difference can be attributed to a weak intramolecular hydrogen bond in conformation C2, between HO-3 and O-1, which is not possible in conformation C1. The semi-empirical PM3 method gives less weight to non-ideal intramolecular hydrogen bonds, as compared to the other methods, and assigns virtually the same energy to both conformations of **3**, while still orienting HO-3 towards the seven-membered ring. Since DMSO solvent molecules compete for HO-3 hydrogen bonding, it is more likely that the intramolecular bond is not an important feature of ring conformation C2 and that, in reality, both conformations have approximately the same energy.

Both model conformations of **3** predict hydrogen-hydrogen distances that are similar to within 0.2 Å, with the exception of some distances to the methyl group (H_3_-11). The most notable are to the diastereotopic hydrogens (H_2_-2), partially presented in Figure 8. While both conformations show almost the same distance between H-2a and H-11, a difference is predicted between H-2b and H-11 if the conformation C1 predominates, which should be apparent in the build-up rate of NOESY cross-peaks for small mixing times. Alternatively, the predominance of the conformation C2 would be indicated by two equal strength cross-peaks for H-2a and H-2b in cross-relaxation with H-11. It is observed that the H-2b (δ_H_ 3.84, d, *J* = 11.1 Hz)/H-11 NOESY cross-peak is weak while the H-2a (δ_H_ 4.02, dd, *J* = 11.1, 1.6 Hz)/H-11 is medium, suggesting that the conformation C1 predominates. NOE effective distances, r_eff_, are calculated by [13] :

$$r_{eff}={\left(\frac{1}{3}\sum_{i}r_{H-2/H-10_{i}}^{-6}\right)}^{-1/6}$$

The average effective positions of the three methyl H-11_i_ protons are relative to H-2a or H-2b. The predicted ratio *r*_eff_ (H-2a/H-11)/*r*_eff_ (H-2b/H-11) is 1.40 (1.36, if *r*^−3^ averages are used instead of *r*^−6^). Assuming that the cross-relaxation rate is similar in both cases, the NOE intensities should also have a similar ratio [14]. Since the observed intensities ratio is actually closer to two, the evidence points towards the predominance of the conformation C1. Since, as stated previously, both conformations have similar conformational energy, the higher stability of 3*R*-C1 of **3** (or of its stereoisomer 3*S*-C2) must originate from the ready interaction of the equatorial HO-3 with the hydrogen-bonding solvent and also from a higher entropic rotational freedom of the hydroxyl and methyl groups. The NOESY spectrum also revealed the correlations of both H_2_-2 to H-9. However, only one of the H_2_-2, i.e., the doublet at δ_H_ 3.84 (*J* = 11.1 Hz), showed a weak cross-peak to H-4, while the double doublet at δ_H_ 4.02 (*J* = 11.1, 1.6 Hz) did not give any cross-peak to H-4. This observation led to the conclusion that the plucked oxepin ring should adopt the conformation in which H-2 at δ_H_ 3.84 is near H-4, i.e., in the α (axial), while H-2 at δ_H_ 4.02 is in β (equatorial) positions, which is in agreement with our conformational analysis. A literature search revealed that compound **3** is also a new compound, so we named it quadricinctoxepine.

**Figure 9:**
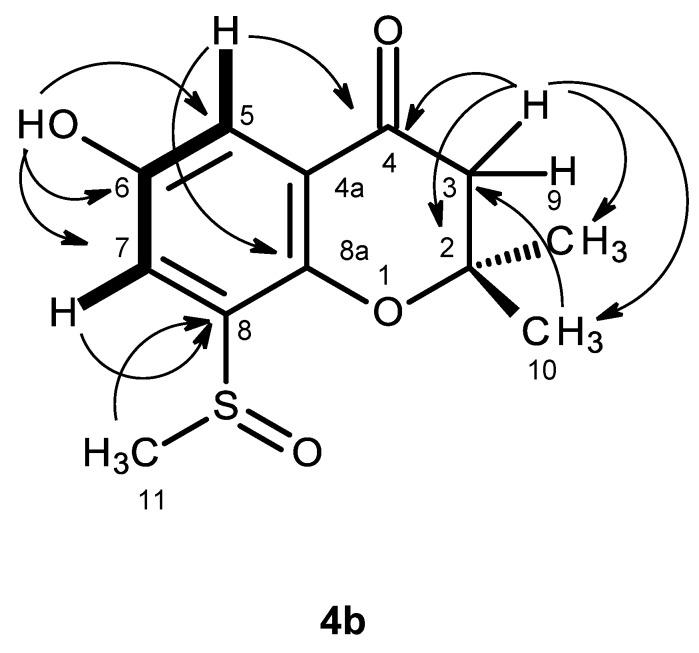
Key COSY (**–**) and HMBC (**→**) correlations for compound **4b**.

**Figure 10:**
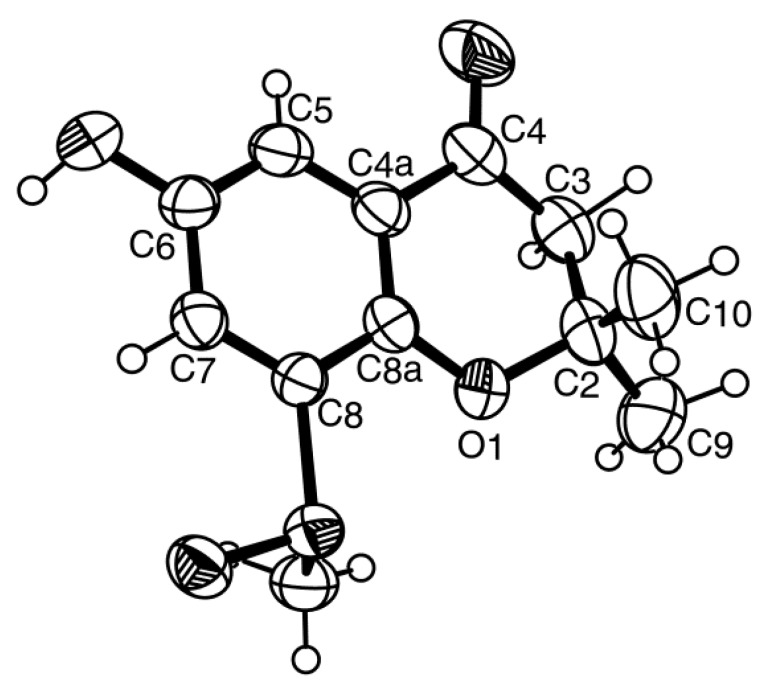
ORTEP diagram of compound **4b**.

Compound **4b** was isolated as white crystals (mp, 227–228 °C), and its molecular formula C_12_H_14_O_4_S was established on the basis of the (+)-HRESIMS *m/z* 255.0694 [M + H]^+^ (calculated 255.0691). The IR spectrum showed absorption bands for hydroxyl (3442 cm^−1^), conjugated ketone carbonyl (1690 cm^−1^) and aromatic (1622 cm^−1^) groups. The ^13^C NMR, DEPT and HSQC spectra (Table 4, Supplementary Information, Figures S26 and S28) exhibited the signals of one conjugated ketone carbonyl (δ_C_ 191.2), four quaternary sp^2^ (δ_C_ 151.6, 147.4, 135.7, 120.8), two methine sp^2^ (δ_C_ 118.0, 112.4), one oxy-quaternary sp^3^ (δ_C_ 80.9), one methylene sp^2^ (δ_C_ 48.0) and three methyl (δ_C_ 40.8, 26.1 and 25.7) groups. The ^1^H NMR spectrum (Table 4, Supplementary Information, Figure S25) exhibited, besides a broad singlet of the phenolic hydroxyl group at δ_H_ 9.85, the signals of two *meta*-coupled aromatic protons at δ_H_ 7.17, d (*J* = 3.1 Hz) and δ_H_ 7.35, d (*J* = 3.1 Hz), two geminally-coupled methylene protons at δ_H_ 2.80, d (*J* = 16.6 Hz) and 2.82, d (*J* = 16.6 Hz) and three methyl singlets at δ_H_ 1.38, s, 1.39, s, and 2.77, s. The chemical shift value of the methyl singlet at δ_H_ 2.77, s (δ_C_ 40.8), indicated that it was on the electron-withdrawing moiety. That compound **4b** was a 2,2,6,8-tetrasubstituted 2,3-dihydro-4*H*-chromen-4-one derivative was supported by the HMBC correlations of H-5 (δ_H_ 7.17, d, *J* = 3.1 Hz) to C-4 (δ_C_ 191.2), C-7 (δ_C_ 118.0) and C-8a (δ_C_ 147.4), of H-7 (δ_H_ 7.35, d, *J* = 3.1 Hz) to C-5 (δ_C_ 112.4), C-8 (δ_C_135.7), C-8a, as well as of H-2 (δ_H_ 2.80, d, *J* = 16.6 Hz and 2.82, d, *J* = 16.6 Hz) to C-3 (δ_C_ 80.9) and C-4 (Table 4, Figure 9, Supplementary Information, Figure S28). As the HMBC spectrum also exhibited cross-peaks of a broad singlet of the phenolic hydroxyl proton at δ_H_ 9.85 to C-5, C-6 (δ_C_ 151.6) and C-7 and of the singlets of the methyl groups at δ_H_ 1.38 and 1.39 to C-2 (δ_C_ 48.0) and C-3, the hydroxyl group was placed on C-6, and the two methyl groups were placed on C-2. Since this partial structure accounted for only C_11_H_11_O_3_, another portion of the molecule must contain CH_3_SO. That the methyl sulfoxide group was on C-8 was supported by the presence of the deshielded methyl group at δ_H_ 2.77, s (δ_C_ 40.8), as well as by the HBMC cross-peak of H_3_-11 (δ_H_ 2.77, s) to C-8. Final proof of the structure of compound **4b** was provided by its X-ray analysis, and its ORTEP view is shown in Figure 10. Moreover, the ORTEP view also revealed that the absolute configuration of the sulfoxide group in compound **4b** is *R*. A literature search showed that compound **4b** is a new compound, and thus, we have named it quadricinctone B.

**Figure 11:**
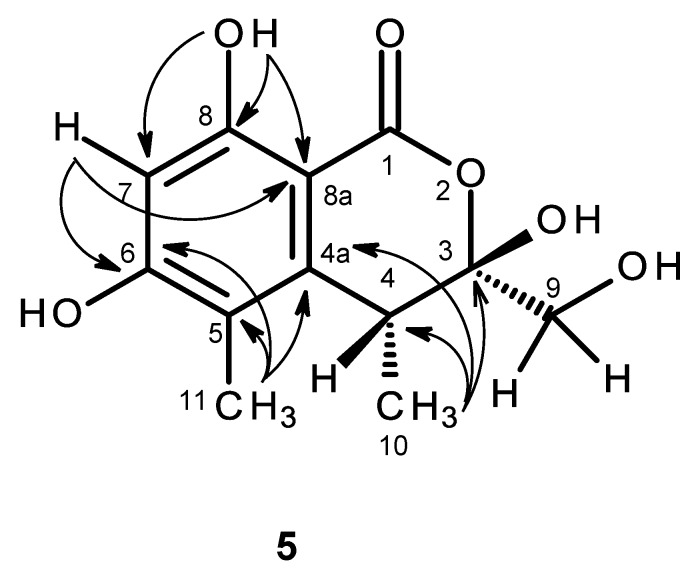
Key HMBC (→) correlations for compound **5**.

**Figure 12:**
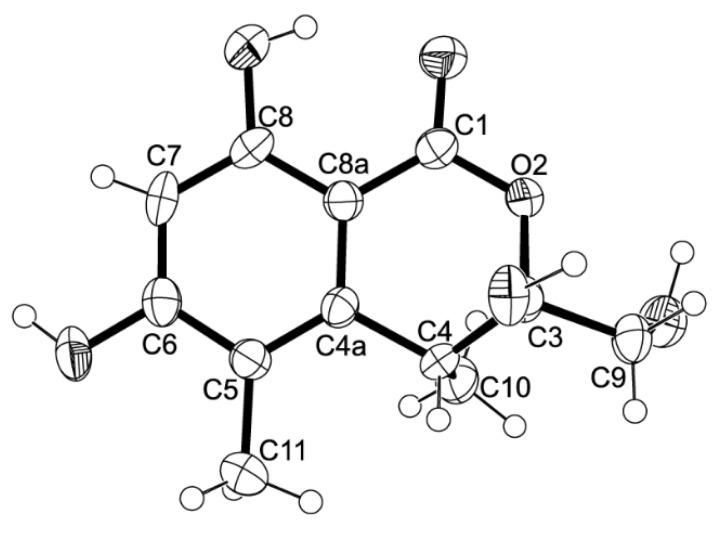
ORTEP diagram of compound **5**.

The molecular formula C_12_H_14_O_6_ of compound **5** was determined based on the (+)-HRESIMS *m/z* 255.0875 (calculated 255.0869), indicating six degrees of unsaturation. The IR spectrum exhibited absorption bands for hydroxyl (3439 cm^−1^), conjugated ester carbonyl (1660 cm^−1^) and aromatic (1643 cm^−1^) groups. The ^13^C NMR, DEPT and HSQC spectra (Table 5, Supplementary Information, Figures S31 and S33) exhibited the signals of one conjugated ester carbonyl (δ_C_ 168.8), five quaternary sp^2^ (δ_C_ 162.9, 161.3, 144.6, 113.5, 98.9), one methine sp^2^ (δ_C_ 99.9), one quaternary sp^3^ of a hemiketal (δ_C_ 104.8), one methine sp^3^ (δ_C_ 34.9), one oxymethylene sp^3^ (δ_C_ 63.7) and two methyl (δ_C_ 16.3 and 9.8) carbons. The ^1^H NMR spectrum (Table 5, Supplementary Information, Figure S30) exhibited, besides a singlet of one aromatic proton at δ_H_ 6.27, a singlet of a hydrogen-bonded phenolic hydroxyl at δ_H_ 11.24 and a broad signal of another phenolic hydroxyl at δ_H_ 10.62, two broad signals of the hydroxyl groups at δ_H_ 5.26 and 7.07, one broad doublet of two methylene protons at δ_H_ 3.65 (*J* = 16.6 Hz), a broad signal of one methine proton at δ_H_ 3.26, one methyl doublet at δ_H_ 1.06 (*J* = 7.2 Hz) and one methyl singlet at δ_H_ 1.98. That compound **5** was a 3,3,4,5,6,8-hexasubstituted 3,4-dihydro-1*H*-isochromen-1-one was supported by the HBMC correlations of OH-8 (δ_H_ 11.24, s) to C-8 (δ_C_ 161.3), C-7 (δ_C_ 99.9), C-8a (δ_C_ 98.9) and of H-7 (δ_H_ 6.27, s) to C-5 (δ_C_ 113.5), C-6 (δ_C_ 162.9), C-8 and C-8a (Table 5, Figure 11, Supplementary Information, Figure S34). Since the methyl singlet at δ_H_ 1.98 gave HMBC cross-peaks to C-5 and the carbons at δ_C_ 144.6 and 162.9 (Table 5, Figure 11), the methyl group (δ_H_ 1.98; δ_C_ 9.8) and another hydroxyl group (δ_H_ 10.62, br) were placed on C-5 and C-6, respectively, and the carbon signals at δ_C_ 144.6 and 162.9 were assigned for C-4a and C-6, respectively. On the other hand, the COSY spectrum (Table 5, Supplementary Information, Figure S32) showed cross-peaks of the broad signal at δ_H_ 3.26 to the methyl doublet at δ_H_ 1.06 (*J* = 7.2 Hz) and of the broad signal at δ_H_ 5.26 to the broad doublet at δ_H_ 3.65 (*J* = 16.6 Hz), while the HMBC spectrum (Table 5, Figure 11) gave cross-peaks of the methyl doublet at δ_H_ 1.06 (*J* = 7.2 Hz) to C-4a, C-4 (δ_C_ 34.9), and the signal of the quaternary carbon at δ_C_ 104.8 (C-3), the methyl and the hydroxymethyl substituents were placed on C-4 and C-3, respectively. Therefore, another hydroxyl group (δ_H_ 7.07, br) was on C-3. This was supported by the chemical shift value of C-3, which is typical for a hemiketal carbon. Since compound **5** could be obtained as a suitable crystal (mp. 223–224 °C), its X-ray analysis was performed. The ORTEP view of compound **5**, shown in Figure 12, not only confirmed the proposed structure, but also determined the absolute configuration of C-3 and C-4 as 3*S*, 4*R*. Since compound **5** is a new compound, it was named quadricinctone C.

**Figure 13:**
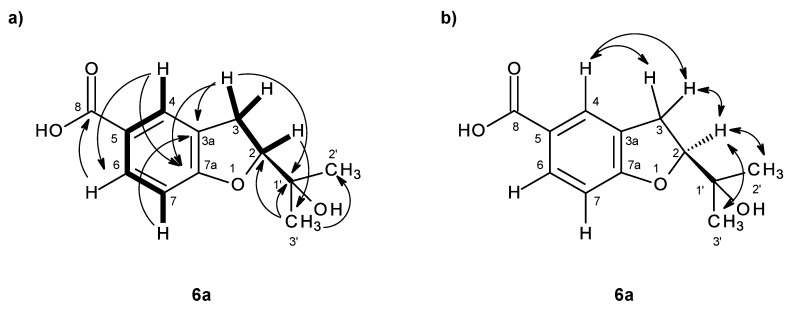
Key COSY (–), HMBC (→) (**a**) and NOESY (↔) (**b**) correlations for compound **6a**.

**Figure 14:**
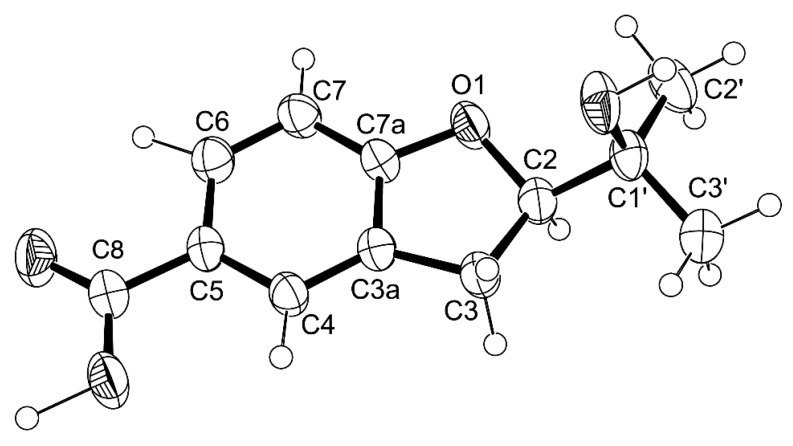
ORTEP diagram of **6a**.

Compound **6a** was also isolated as white crystals (mp. 149–150 °C), and its molecular formula C_12_H_14_O_4_ was determined based on the (+)-HRESIMS *m/z* 223.9067 [M + H]^+^ (calculated 223.9070), indicating six degrees of unsaturation. The IR spectrum showed absorption bands for hydroxyl (3417 cm^−1^), a conjugated carbonyl (1681 cm^−1^) and aromatic (1634 cm^−1^) groups. The ^13^C NMR, DEPT and HSQC spectra (Table 6, Supplementary Information, Figures S36 and S38) exhibited the signals of one conjugated carboxyl carbonyl (δ_C_ 167.2), two quaternary sp^2^ (δ_C_ 128.2, 122.6), one oxy-quaternary sp^2^ (δ_C_ 163.7), three methine sp^2^ (δ_C_ 130.4, 126.4, 108.4), one oxy-quaternary sp^3^ (δ_C_ 70.0), one oxymethine sp^3^ (δ_C_ 90.2), one methylene sp^2^ (δ_C_ 29.3) and two methyl (δ_C_ 25.9 and 24.9) carbons. The ^1^H NMR spectrum (Table 6, Supplementary Information, Figure S35) showed signals of three aromatic protons of the 1,2,4-trisubstituted benzene ring at δ_H_ 7.75, d (*J* = 1.8 Hz), 7.72, dd (*J* = 8.3, 1.8 Hz), 6.80, d (*J* = 8.3 Hz), in addition to a broad signal of the hydroxyl proton at δ_H_ 12.49, a triplet of one proton at δ_H_ 4.64 (*J* = 8.9 Hz), a doublet of two protons at δ_H_ 3.17 (*J* = 8.9 Hz) and two methyl singlets at δ_H_ 1.13 and 1.14. That the 1,2,4-trisubstituted benzene ring was part of 2,5-disubstituted 2,3-dihydro-1-benzofuran was supported by the COSY correlations of H-6 (δ_H_ 7.72, dd, *J* = 8.3, 1.8 Hz) to H-4 (δ_H_ 7.75, d, *J* = 1.8 Hz) and H-7 (δ_H_ 6.80, d, *J* = 8.3 Hz) and of H-2 (δ_H_ 3.17, *J* = 8.9 Hz) to H-3 (δ_H_ 4.64, *J* = 8.9 Hz)(Table 6, Figure 13a, Supplementary Information, Figure S37), as well as of the HMBC correlations of H-4 to C-6 (δ_C_ 130.4) and C-7a (δ_C_ 163.7), of H-6 to C-4 (δ_C_ 126.4), of H-3 to C-2 (δ_C_ 90.2), C-3a (δ_C_ 128.2) and C-7a and of H-7 to C-3a, C-7a and C-5 (δ_C_ 122.6) (Table 6, Figure 13a, Supplementary Information, Figure S39). That the carboxyl group was on C-5 was supported by the HMBC correlations of H-4 and H-6 to the conjugated carbonyl at δ_C_ 167.2 (Table 6, Figure 13a), along with the presence of a broad signal at δ_H_ 12.49, which is characteristic of a hydroxyl group of carboxylic acid. In the same manner, that the 2-hydroxypropyl substituent was placed on C-2 was evidenced by the HMBC correlations of H-2 (δ_H_ 4.64, *J* = 8.9 Hz) to C-3 (δ_C_ 29.3), C-1′ (δ_C_ 70.0), C-2′ (δ_C_ 24.9), C-3′ (δ_C_ 25.9) (Table 6, Figure 13a), as well as by the NOESY correlations of H-2 to H-3, H_3_-2′ (δ_H_ 1.14, s) and H_3_-3′ (δ_H_ 1.13, s)(Figure 13b). Therefore, compound **6a** was identified as 2-(2-hydroxypropan-2-yl)-2,3-dihydro-1-benzofuran-5-carboxylic acid. A literature search revealed that compound **6a** has the same flat structure as anodendroic acid (2-(1-hydroxy-1-methylethyl)-2,3-dihydrobenzofuran-5-carboxylic acid), which was first isolated from the stem of *Anodendron affine* Durce [15]. Anodendroic acid was later synthesized by Yamaguchi et al. [16]; however, its ^1^H NMR data (DMSO-*d*_6_) were slightly different from those reported by Shima et al. [15]. Anodendroic acid was also obtained by basic hydrolysis of its methyl ester, a constituent of *Eriodictyon sessilifolium* [17], and also by biotransformation of 3-(γ,γ-dimethylallyl)-*p*-coumaric acid [18]. It is interesting to note that, in all of these reports, the identity of anodendroic acid was achieved by comparing its melting point, ^1^H NMR and MS data with those reported by Shima et al. [15], while no stereochemistry of C-2 was indicated. However, when compared to the ^1^H NMR and other physical data of compound **6a** with those reported for anodendroic acid by Shima et al. [15], it was found that the ^1^H NMR data of anodendroic acid (in pyridine-*d*_6_) [15] were slightly different from those of compound **6a** (in DMSO-*d*_6_). Therefore, it was not possible to compare these ^1^H NMR data, since they were obtained in different solvents. Interestingly, the ^1^H NMR data, obtained in DMSO-*d*_6_ by Yamaguchi et al. [16], were also slightly different from those of compound **6a**. The obvious differences between anodendroic acid [15] and compound **6a** are their melting points and optical rotations. While anodendroic acid (mp. 212–214 °C) is laevorotatory (${\left[\alpha \right]}_{D}^{26}$ −19°, *c* 0.7, EtOH) [15], compound **6a** (mp. 149–150 °C) is dextrorotatory (${\left[\alpha \right]}_{D}^{20}$ +74°, *c* 0.03, MeOH). Consequently, we concluded that the structure of compound **6a** is different from that of anodendroic acid. This fact has prompted us to investigate the stereochemistry of compound **6a**. Since Compound **6a** was obtained as a suitable crystal for X-ray diffraction, its X-ray analysis was carried out. The ORTEP view of compound **6a** shown in Figure 14 not only confirmed its proposed structure, but also revealed the absolute configuration for C-2 as 2*R*. Therefore, compound **6a** is a new compound, and we have named it quadricinctafuran A. Since anodendroic acid exhibited the opposite sign of rotation to that of compound **6a**, it is probable that the absolute configuration of its C-2 is 2*S*. This is not unusual, since fomannoxin, another 2,3-dihydrobenzofuran derivative, also showed the opposite optical rotation and has the opposite absolute configuration to tremetone, even though they have very similar structures [16].

**Figure 15:**
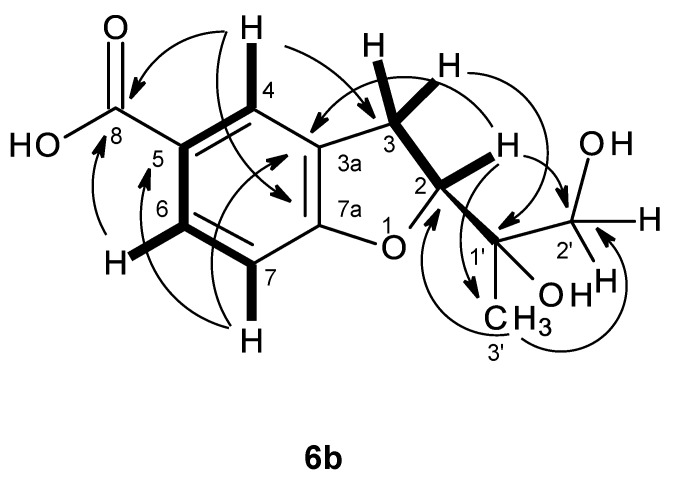
Key COSY (**–**) and HMBC (**→**) correlations for compound **6b**.

The (+)-HRESIMS of compound **6b** gave the [M + H]^+^ at *m/z* 239.0919 (calculated 239.0919), indicating its molecular formula C_12_H_14_O_5_ and, therefore, six degrees of unsaturation. The general features of its ^1^H and ^13^C NMR spectra resembled those of compound **6a**. The ^13^C NMR, DEPT and HSQC spectra (Table 6, Supplementary Information, Figures S42 and S44) exhibited the signals of one conjugated carboxyl carbonyl (δ_C_ 167.2), two quaternary sp^2^ (δ_C_ 128.4, 122.6), one oxy-quaternary sp^2^ (δ_C_ 163.6), three methine sp^2^ (δ_C_ 130.8, 126.4, 108.4), one oxy-quaternary sp^3^ (δ_C_ 72.5), one oxymethine sp^3^ (δ_C_ 86.5), one methylene sp^2^ (δ_C_ 28.7), one oxymethylene sp^2^ (δ_C_ 66.7) and one methyl (δ_C_ 20.0) carbons. The ^1^H NMR and COSY spectra (Table 6, Supplementary Information, Figures S41 and S43) showed similar proton signals of the 2,3-dihydro-1-benzofuran-5-carboxylic acid nucleus to those of **6a**. However, it exhibited only one methyl singlet at δ_H_ 1.09 and another singlet of oxymethylene protons at δ_H_ 3.33, instead of two methyl singlets as in compound **6a**. Therefore, the difference between the structures of compounds **6a** and **6b** resides in the substitutions on C-2. That the substituent on C-2 of compound **6b** was 1,2-dihydroxypropyl was evidenced by not only its molecular formula (C_12_H_14_O_5_), which has one more oxygen atom than that of compound **6a** (C_12_H_14_O_4_), but also the presence of the oxymethylene carbon at δ_C_ 66.7 (C-2′; δ_H_ 3.33, s). Moreover, the chemical shift value of C-2 (δ_C_ 86.5) was nearly 4 ppm lower than that of compound **6a** (δ_C_ 90.2). Thus, compound **6b** was identified as 2-(1,2-dihydroxypropan-2-yl)-2,3-dihydro-1-benzofuran-5-carboxylic acid. This was confirmed by the HMBC correlations of H_3_-3′ (δ_H_ 1.09) to C-1′ (δ_C_ 72.5) and C-2′ (δ_C_ 66.7), as well of H_2_-2′ (δ_H_ 3.33, s) to C-1′ (δ_C_ 72.5) and C-3′ (δ_C_ 20.0) (Figure 15, Supplementary Information, Figure S45). Since compound **6b** could not be obtained as suitable crystals for X-ray crystallography, it was not possible to determine the absolute configuration of C-2 by X-ray analysis. Moreover, as compound **6b** also has a tertiary hydroxyl group on C-1′ and a primary hydroxyl group on C-2′, it was not possible to determine the absolute configurations of C-2 and C-1′ by Mosher’s method. On the other hand, since the methyl group on C-1 in compound **6a** was replaced by the hydroxymethyl group incompound **6b**, it is legitimate to postulate that compounds **6b** is derived from compound **6a**. Consequently, the absolute configuration of C-2 of compound **6b** should be the same as that of compound **6a**, i.e., 2*R*. However, it is not yet possible to determine the absolute configuration of C-1′. Compound **6b** is also a new compound, and we have named it quadricinctafuran B.

**Figure 16:**
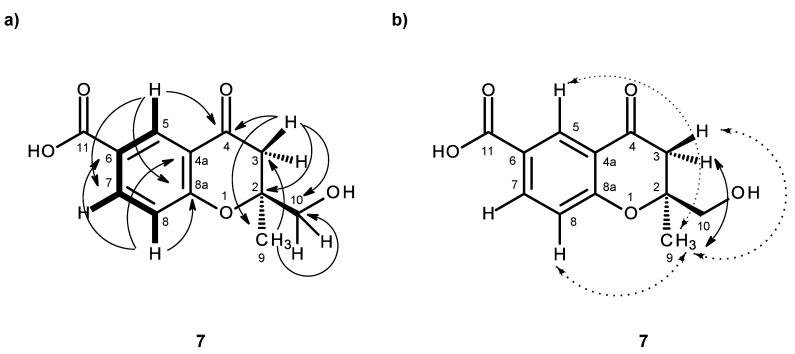
Key COSY (–), HMBC (→) (**a**) and NOESY C-2 (↔) (**b**) C-2 for compound **7**.

**Figure 17:**
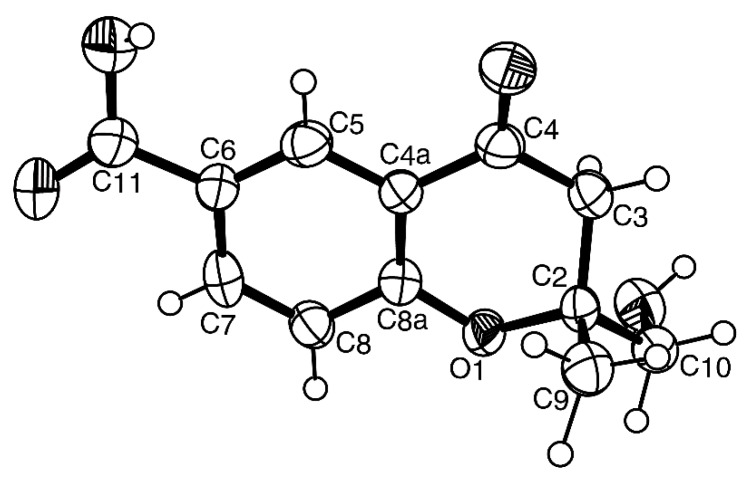
ORTEP diagram of compound **7**.

Compound **7** was also isolated as white crystals (mp. 196–197 °C), and its molecular formula C_14_H_12_O_5_ was established on the basis of the (+)-HRESIMS *m/z* 237.0792 [M + H]^+^ (calculated 237.0763), indicating seven degrees of unsaturation. The IR spectrum showed absorption bands for hydroxyl (3404 cm^−1^), conjugated ketone carbonyl (1708 cm^−1^), conjugated carboxyl carbonyl (1670 cm^−1^) and aromatic (1558, 1540 cm^−1^) groups. The ^13^C NMR, DEPT and HSQC spectra (Table 7, Supplementary Information, Figures S48 and S50) exhibited the signals of one conjugated ketone carbonyl (δ_C_ 191.1), one conjugated carboxyl carbonyl (δ_C_ 166.4), one oxy-quaternary sp^2^ (δ_C_ 162.9), two quaternary sp^2^ (δ_C_ 123.0, 119.6), three methine sp^2^ (δ_C_ 136.4, 127.5, 118.5), one oxy-quaternary sp^3^ (δ_C_ 83.0), one oxymethylene sp^2^ (δ_C_ 66.8), one methylene sp^2^ (δ_C_ 43.4) and one methyl (δ_C_ 21.2) carbons. The ^1^H NMR spectra (Table 7, Supplementary Information, Figure S47) exhibited, besides the signals of three aromatic protons of the 1,2,4-trisubstituted benzene ring at δ_H_ 8.26, d (*J* = 2.3 Hz), 8.04, dd (*J* = 8.7, 2.3 Hz), 7.07, d (*J* = 8.7 Hz), a methyl singlet at δ_H_ 1.30, two pairs of geminally-coupled methylene protons at δ_H_ 2.74, d (*J* = 16.7 Hz)/3.00, d (*J* = 16.7 Hz) and δ_H_ 3.49, dd (*J* = 11.6, 4.5 Hz)/3.59, dd (*J* = 11.6, 4.2 Hz); the latter showed COSY correlations with the broad triplet of the hydroxyl proton at δ_H_ 5.26 (Table 7, Figure 16a, Supplementary Information, Figure S49). As the HMBC spectrum exhibited correlations of the aromatic proton signal at δ_H_ 8.26, d (*J* = 2.3 Hz, H-5) to the oxy-quaternary sp^2^ carbon at δ_C_ 162.9 (C-8a), as well as to the conjugated ketone carbonyl carbon at δ_C_ 191.1 (C-4) and the methine sp^2^ at δ_C_ 136.4 (C-7) (Table 7, Figure 16a, Supplementary Information, Figure S51), the ketone moiety was placed on C-4a. Additionally, the HMBC spectrum also showed correlations of H-8 (δ_H_ 7.07, d, *J* = 8.7 Hz) to C-8a and the quaternary sp^2^ carbons at δ_C_ 123.0 and 119.6; they were therefore assigned to C-4a and C-6, respectively (Table 7, Figure 16a). The presence of the 1-hydroxy-2-methyl-2-oxypropyl moiety was supported by the HMBC correlations of the methyl singlet at δ_H_ 1.30 (CH_3_-9) to the oxy-quaternary sp^3^ carbon at δ_C_ 83.0 (C-2), the oxymethylene sp^3^ carbon at δ_C_ 66.8 (C-10) and the methylene sp^3^ carbon at δ_C_ 43.4 (C-3), as well as of the H_2_-3 (δ_H_ 2.74, d, *J* = 16.7 Hz/3.00, d, *J* = 16.7 Hz) to C-2, C-9 (δ_C_ 21.2) and C-10 (Table 7, Figure 16a). Since H_2_-3 also gave a HMBC cross-peak to C-4, the 1-hydroxy-2-methyl-2-oxypropyl moiety was linked to C-4. Due to the fact that these two moieties accounted for only C_11_H_12_O_4_, it was concluded that the carboxyl group (δ_C_ 166.4, δ_H_ 12.69 br) was on C-6. Therefore, compound **7** was identified as 2-(hydroxymethyl)-2-methyl-4-oxo-3,4-dihydro-2*H*-chromene-6-carboxylic acid. As compound **7** was obtained as a suitable crystal for an X-ray diffraction, its X-ray analysis was carried out. The ORTEP view of compound **7**, shown in Figure 17, revealed the absolute configuration for C-2 as 2*S*. A literature search revealed that compound **7** is also a new compound, so we named it quadricinctone D.

In order to establish the conformation of the 2,3-dihydro-*4H*-pyran-4-one, analysis of the NOESY correlations was carried out. The NOESY spectrum (Table 7, Figure 16b, Supplementary Information, Figure S52) exhibited not only a strong correlation of H-7 to H-8, but also week correlations of H_3_-9 to H-5 and H-8. Therefore, CH_3_-9 is in the α-axial position. Additionally, since H_3_-9 also exhibited a strong cross-peak with the signal of the methylene proton at δ_H_ 2.74, d (*J* = 16.7 Hz), and a weak cross-peak with the proton signal at δ_H_ 3.00, d (*J* = 16.7 Hz), the former was assigned to H-3α and the latter to H-3β. It is interesting to observe that the structure of compound **7** is analogous to the structure of compound **3** in that it also exhibits the same methyl-methylene bridge structural feature (at C-9 and C-3), with the same relative intensity of NOESY cross-peaks between the protons of the two groups and with similar conformational energies for the two half-chair conformations of the non-aromatic ring. Therefore, the conclusions drawn for compound **3** are also valid for compound **7**, whose stereochemistry is unequivocally defined by X-ray analysis.

**Figure 18:**
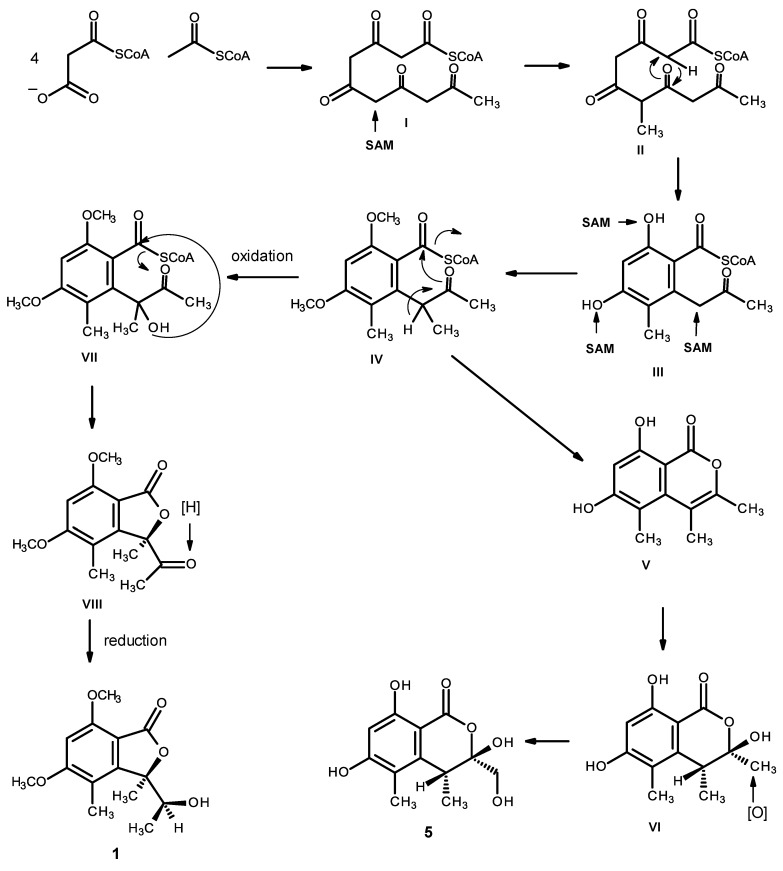
Proposed biosynthetic pathways for compounds **1** and **5**.

Compounds **1** and **5** can be hypothesized as originating from the pentaketide intermediate (I). Methylation (by SAM) gives II, which undergoes cyclization and enolization to give the intermediate III. Methylation of the phenolic hydroxyl groups and the α-carbon of the carbonyl ketone leads to the intermediate IV. Enolization, followed by a lactonization, originates V, which undergoes hydration to give VI, and oxidation of one of the methyl groups gives rise to compound **5**. Alternatively, oxidation of the α-carbon of the side chain of IV leads to the intermediate VII, which, after lactonization and reduction of the ketone carbonyl, gives rise to compound **1** (Figure 18).

**Figure 19:**
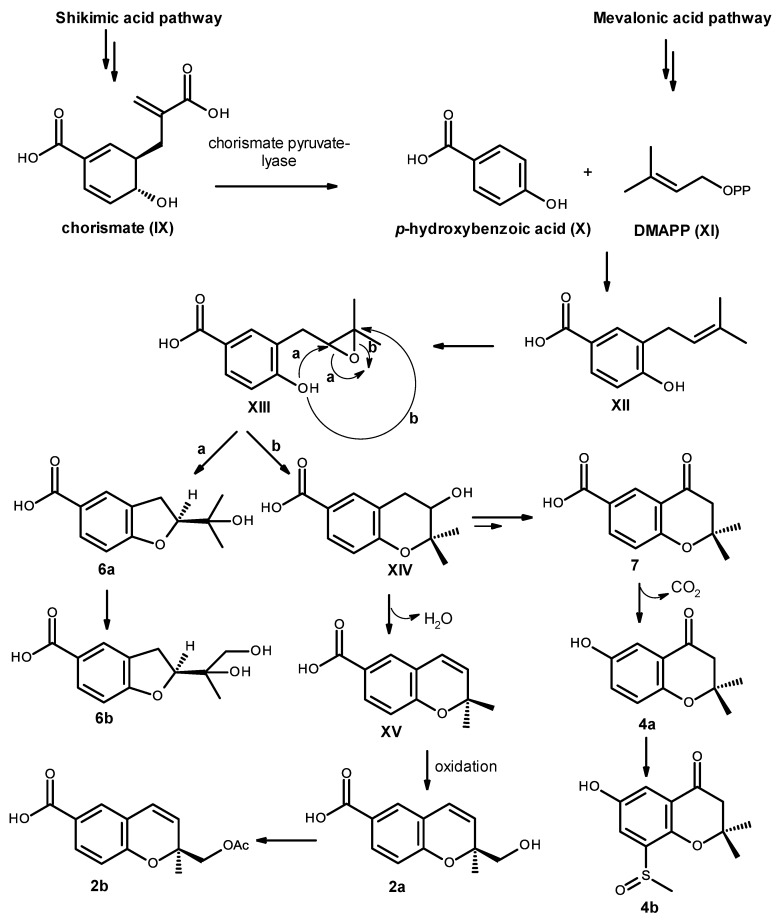
Proposed biosynthetic pathway for compounds **2a**, **2b**, **4a**, **4b**, **6a**, **6b** and **7**.

Biosynthetically, compounds **2a**, **2b**, **4a**, **4b**, **6a**, **6b** and **7** are of mixed origin, i.e., shikimic acid and mevalonic acid pathways, similar to that proposed for fomannoxin [19], as depicted in Figure 19. Elimination of pyruvate from chorismate (IX) by chorismate pyruvate lyase leads to the formation of *p*-hydroxybenzoic acid (X), which after prenylation by DMAPP (XI), originates the intermediate XII. Epoxidation and cyclization of XII, via Route *a*, leads to a formation of the furan ring in compound **6a** and after oxidation of one of the methyl groups leads to compound **6b**. On the other hand, cyclization via Route *b* leads to the formation of the pyran ring in XIV. Dehydration and oxidation of one of the methyl groups originates compound **2a**, which after acetylation of the primary alcohol function of the side chain will originate compound **2b**. Alternatively, the intermediate XIV can also undergo dehydration, reduction and oxidation to give the ketone function in compound **7**. Oxidative decarboxylation of compound **7** leads to the formation of compound **4a**, which after sulfinylation of the benzene ring originates compound **4b**. However, it is possible that the introduction of the methyl sulfoxide group to the aromatic ring could happen before cyclization.

**Figure 20:**
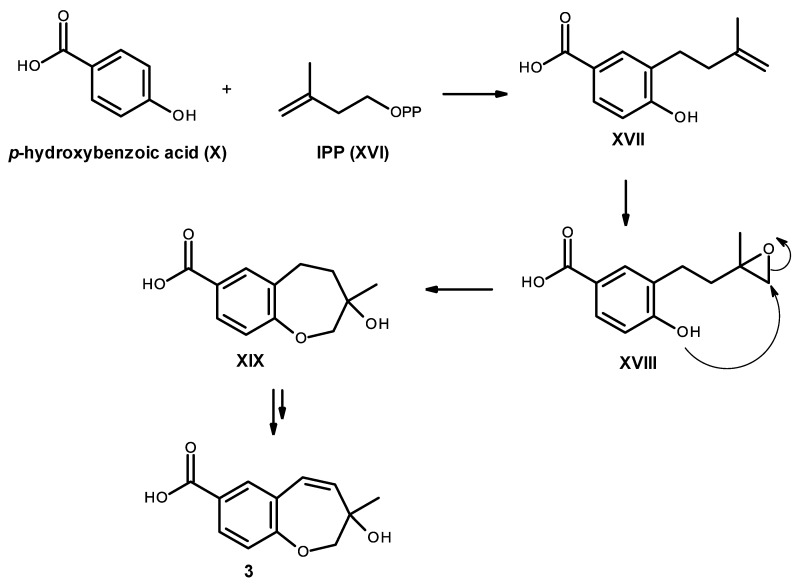
Proposed biosynthetic pathway for compound **3**.

Compound **3** is also derived from a prenylation of *p*-hydroxybenzoic acid (X); however, it can occur with IPP (XVI) instead of DMAPP (XI). Epoxidation of the double bond of the side chain of XVII, followed by cyclization of XVIII leads to the formation of an oxepin ring in XIX. Oxidation and dehydration of the oxepin ring will lead to the formation of compound **3**, as depicted in Figure 20.

Compounds **1**–**7** were evaluated for their antibacterial activity against Gram-positive and Gram-negative bacteria, as well as multidrug-resistant isolates from the environment, according to the previously described protocol [6], as well as for their antifungal activity against yeast (*Candida albicans* ATCC 10231), filamentous fungus (*Aspergillus fumigatus* ATCC 46645) and dermatophyte (*Trichophyton rubrum* FF5) in the antifungal assay [20]. The results showed that none of the tested compounds exhibited significant antibacterial activity (MIC > 256 μg/mL) or antifungal activity (MIC > 512 μg/mL). These compounds were also evaluated for their in vitro growth inhibitory activity against the MCF-7 (breast adenocarcinoma), NCI-H460 (non-small cell lung cancer) and A375-C5 (melanoma) cell lines by the protein binding dye SRB method [21], and they did not show any activity in this assay (GI_50_ > 150 mM).

## 3. Experimental Section

### 3.1. General Procedure

Melting points were determined on a Bock monoscope and are uncorrected. Optical rotations were measured on an ADP410 Polarimeter (Bellingham + Stanley Ltd., Tunbridge Wells, Kent, U.K.). Infrared spectra were recorded in a KBr microplate in a FTIR spectrometer Nicolet iS10 from Thermo Scientific (Waltham, MA, USA) with the Smart OMNI-Transmission accessory (Software 188 OMNIC 8.3). UV spectra were taken in CHCl_3_ and were recorded on a Varian CARY 100 spectrophotometer. ^1^H and ^13^C NMR spectra were recorded at ambient temperature on a Bruker AMC instrument (Bruker Biosciences Corporation, Billerica, MA, USA) operating at 300.13 and 75.4 MHz, respectively. High resolution mass spectra were measured with a Waters Xevo QToF mass spectrometer (Waters Corporations, Milford, MA, USA) coupled to a Waters Acquity UPLC system. A Merck (Darmstadt, Germany) silica gel GF_254_ was used for preparative TLC, and a Merck Si gel 60 (0.2–0.5 mm) was used for column chromatography.

### 3.2. Extraction and Isolation

The strain KUFA 0081 was isolated from the marine sponge *Clathria reinwardti*, which was collected, by scuba diving at a depth of 15–20 m, from the coral reef at Samae San Island (12°34′ 36.64″ N 100°56′ 59.69″ E) in the Gulf of Thailand, Chonburi Province, in July 2013. The sponge was washed with 0.06% sodium hypochlorite solution for 1 min, followed by sterilized seawater 3 times and then dried on sterile filter paper, cut into small pieces (5 × 5 mm) and placed on a malt extract agar (MEA) medium containing 70% seawater and 300 mg/L of streptomycin sulphate, then incubated at 28 °C for 7 days, after which the hyphal tips were transferred onto a slant MEA and maintained as pure culture for further identification. The fungus was identified as *Neosartorya quadricincta* (E. Yuill) Malloch & Cain by one of us (T.D.), based on morphological characteristics, such as colony growth rate and growth pattern on standard media, namely Czapek’s agar, Czapek yeast autolysate agar and malt extract agar. Microscopic characteristics, including size, shape and the ornamentation of ascospores, were examined under light and scanning electron microscopes. This identification was supported by sequence analysis of the β-tubulin, calmodulin and actin genes as described in the previous report [22]. *Neosartorya quadricincta* was also confirmed by the analysis sequence of the internal transcribed spacer (ITS) gene according the procedure previously described by us [7]. Its gene sequences were deposited in GenBank with Accession Numbers KM095492 and KT201525, respectively. The pure cultures were deposited as KUFA0081 at Kasetsart University Fungal Collection, Department of Plant Pathology, Faculty of Agriculture, Kasetsart University, Bangkok, Thailand.

The fungus was cultured for one week at 28 °C in 10 Petri dishes (i.d. 90 mm) containing 25 mL of MEA. In order to obtain the mycelial suspension, the mycelial plugs were transferred to two 500-mL Erlenmeyer flasks containing 200 mL of potato dextrose broth and then incubated on a rotary shaker at 120 rpm at 28 °C for 4 days. Fifty 1000-mL Erlenmeyer flasks, each containing 300 g of cooked rice, were autoclaved at 121 °C for 15 min, then inoculated with 20 mL of mycelial suspension of *N. quadricincta* and incubated at 28 °C for 30 days, after which the mouldy rice was macerated in ethyl acetate (25 L total) for 7 days and then filtered. The ethyl acetate solution was concentrated under reduced pressure to yield 176.5 g of crude ethyl acetate extract, which was dissolved in 500 mL of CHCl_3_ and then washed with H_2_O (3 × 500 mL). The organic layers were combined and dried with anhydrous Na_2_SO_4_, filtered and evaporated under reduced pressure to give 31.1 g of the crude chloroform extract, which was applied on a column of silica gel (440 g) and eluted with mixtures of petrol-CHCl_3_ and CHCl_3_-Me_2_CO; 250-mL fractions were collected as follows: Frs 1–15 (petrol-CHCl_3_, 1:1), 16–55 (petrol-CHCl_3_, 3:7), 56–118 (petrol-CHCl_3_, 1:9), 119–284 (CHCl_3_-Me_2_CO, 9:1), 285–329 (CHCl_3_-Me_2_CO, 7:1), 330–359 (CHCl_3_-Me_2_CO, 1:1). Frs 175–181 were combined (250 mg) and purified by TLC (silica gel G254, CHCl_3_-Me_2_CO-HCO_2_H, 3:2:0.01) to give 86.8 mg of **1** and 15.0 mg of **2b**. Frs 182–197 were combined (565.1 mg) and purified by TLC (silica gel G254, CHCl_3_-Me_2_CO-HCO_2_H, 17:3:0.02) to give 35.1 mg of **1** and 14.3 mg of **2b**. Frs 224–235 were combined (249.3 mg) and purified by TLC (silica gel G254, CHCl_3_-Me_2_CO-HCO_2_H, 4:1:0.01) to give 11.4 mg of **4a** [12]. Frs 236–285 were combined (1.2 g), applied over a column chromatography of silica gel (42 g) and eluted with mixtures of petrol-CHCl_3_ and CHCl_3_-Me_2_CO, wherein 100-mL subfractions were collected as follows: sfrs 1–17 (petrol-CHCl_3_, 1:9), 18–61 (CHCl_3_-Me_2_CO,9:1). Sfrs 24–33 were combined (423.4 mg) and precipitated in a mixture of CHCl_3_ and Me_2_CO to give a white solid (46.2 mg), which was further recrystallized in a mixture of CHCl_3_ and Me_2_CO to give 9.4 mg of white crystals of **6a**, and the mother liquor was purified by TLC (silica gel G254, CHCl_3_-Me_2_CO-HCO_2_H, 9:1:0.01) to give 23.2 mg of **6a** and 4.9 mg of **2a**. The mother liquor of sfrs 24–33 (133.0 mg) was purified by TLC (silica gel G254, CHCl_3_-Me_2_CO-HCO_2_H, 7: 3:0.01) to give 41.1 mg of **2a** and 12.4 mg of **3**. Sfrs 34–39 were combined (77.7 mg) and purified with TLC (silica gel G254, CHCl_3_-Me_2_CO-HCO_2_H, 3: 1:0.01) to give 7.9 mg of **4b** and 17.1 mg of **2a**. Sfrs 40–61 were combined (496.4 mg) and purified by TLC (silica gel G254, CHCl_3_-Me_2_CO-HCO_2_H, 4: 1:0.01) to give 22.7 mg of **5**. Frs 307–315 were combined (291.0 mg), applied over a column chromatography of Sephadex LH-20 (10 g) and eluted with a 1:1 mixture of CHCl_3_: MeOH to give twelve 1-mL sub-fractions. Sfrs 9–12 were combined and purified by TLC (silica gel G254, CHCl_3_-Me_2_CO-HCO_2_H, 3: 2:0.01) to give 13.4 mg of **6b** and 9.4 mg of **7**. Frs 316–359 were combined (1.2 g), applied over a column chromatography of Sephadex LH-20 (10 g) and eluted with a 1:1 mixture of CHCl_3_: MeOH to give twenty-one 1-mL sub-fractions. Sfrs 12–21 were combined (645.7 mg), applied over a column chromatography of Sephadex LH-20 (10 g) and eluted with a 1:1 mixture of CHCl_3_: MeOH to give sixteen 1-mL fractions. Ssfrs 5–16 were combined (597.5), applied over a column chromatography of silica gel (15 g) and eluted with a mixture of petrol-CHCl_3_ and CHCl_3_-Me_2_CO, wherein 100-mL fractions were collected as follows: frs 1–8 (petrol-CHCl_3_, 1:9), 9–28 (CHCl_3_-Me_2_CO, 9:1), 29–41 ((CHCl_3_-Me_2_CO, 7:3). Frs 17–18 were combined (47.4 mg) and purified by TLC (silica gel G254, CHCl_3_-Me_2_CO-HCO_2_H, 3: 2:0.01) to give 20.8 mg of **6b**.

#### 3.2.1. Quadricinctone A: 3-(1-Hydroxyethyl)-5,7-dimethoxy-3,4-dimethyl-2-benzofuran-1(3*H*)-one (**1**)

White crystals; mp 176–177 °C (petrol/CHCl_3_); ${\left[\alpha \right]}_{D}^{20}$ −59° (*c* 0.05, CHCl_3_); UV (MeOH) λ_max_ (log ε) 233 (4.39), 260 (4.03), 298 (3.78) nm; IR (KBr) υ_max_ 3455, 2981, 2490, 1723, 1612, 1596, 1499, 1467, 1432, 1228 cm^−1^; ^1^H and ^13^C NMR, see Table 1; HRESIMS *m/z* 267.1243 [M + H]^+^ (calculated for C_14_H_19_O_5_, 267.1332).

#### 3.2.2. Quadricinctapyran A: 2-(Hydroxymethyl)-2-methyl-2*H*-chromene-6-carboxylic acid (**2a**)

White crystals; mp 147–148 °C (CHCl_3_/Me_2_CO); ${\left[\alpha \right]}_{D}^{20}$ +30° (*c* 0.03, MeOH); UV (MeOH) λ_max_ (log ε) 237 (4.61) nm; IR (KBr) υ_max_ 3447, 2922, 2359, 2341, 1696, 1647, 1609, 1578, 1490, 143, 1301, 1267 cm^−1^; ^1^H and ^13^C NMR, see Table 2; HRESIMS *m/z* 221.0820 [M + H]^+^ (calculated for C_12_H_13_O_4_, 221.0814).

#### 3.2.3. Quadricinctapyran B: 2-[(Acetyloxy)methyl]-2-methyl-2*H*-chromene-6-carboxylic acid (**2b**).

White solid; mp 118–119 °C (petrol/CHCl_3_); ${\left[\alpha \right]}_{D}^{20}$ +58° (*c* 0.07, CHCl_3_); UV (MeOH) λ_max_ (log ε) 201 (4.13), 237 (4.47) nm; IR (KBr) υ_max_ 3441, 2986, 2943, 1745, 1678, 1640, 1607, 1574, 1453, 1419, 1297, 1264, 1228 cm^−1^; ^1^H and ^13^C NMR, see Table 2; HRESIMS *m/z* 263.0971 [M + H]^+^ (calculated for C_14_H_15_O_5_, 263.0919).

#### 3.2.4. Quadricinctoxepine: 3-Hydroxy-3-methyl-2,3-dihydro-1-benzoxepine-7-carboxylic acid (**3**)

White solid; mp 189–191 °C (CHCl_3_/Me_2_CO); ${\left[\alpha \right]}_{D}^{20}$ +21° (*c* 0.07, MeOH); UV (MeOH) λ_max_ (log ε) 234 (4.50) nm; IR (KBr) υ_max_ 3404, 2921, 2359, 2342, 1701, 1606, 1574, 1497, 1384, 1297, 1259, 1117 cm^−1^; ^1^H and ^13^C NMR, see Table 3; HRESIMS *m/z* 221.0819 [M + H]^+^ (calculated for C_12_H_13_O_4_, 221.0814).

#### 3.2.5. Quadricinctone B: 6-Hydroxy-2,2-dimethyl-8-(methylsulfinyl)-2,3-dihydro-4*H*-chromen-4-one (**4b**)

White crystals; mp 227–228 °C (CHCl_3_/Me_2_CO); ${\left[\alpha \right]}_{D}^{20}$ +30° (*c* 0.03, MeOH); UV (MeOH) λ_max_ (log ε) 205 (4.27), 234 (4.22), 358 (3.64) nm; IR (KBr) υ_max_ 3442, 2975, 2922, 1690, 1622, 1484, 1441, 1417, 1326, 1251, 1177 cm^−1^; ^1^H and ^13^C NMR, see Table 4; HRESIMS *m/z* 255.0694 [M + H]^+^ (calculated for C_12_H_15_O_4_S, 255.0691).

#### 3.2.6. Quadricinctone C: 3,6,8-Trihydroxy-3-(hydroxymethyl)-4,5-dimethyl-3,4-dihydro-1*H*-isochromen-1-one (**5**)

White crystals; mp 223–224 °C (CHCl_3_/Me_2_CO); ${\left[\alpha \right]}_{D}^{20}$ +64° (*c* 0.06, MeOH); UV (MeOH) λ_max_ (log ε) 214 (4.23), 271 (3.96), 312 (3.70) nm; IR (KBr) υ_max_ 3439, 3006, 2976, 2360, 2342, 1660, 1644, 1600, 1494, 1472, 1448, 1385, 1157 cm^−1^; ^1^H and ^13^C NMR, see Table 5; HRESIMS *m/z* 255.0875 [M + H]^+^ (calculated for C_12_H_15_O_6_, 255.0869).

#### 3.2.7. Quadricinctafuran A: (2*R*)-(2-Hydroxypropan-2-yl)-2,3-dihydro-1-benzofuran-5-carboxylic acid (**6a**)

White crystals; mp 149–150 °C (CHCl_3_/Me_2_CO); ${\left[\alpha \right]}_{D}^{20}$ +74° (*c* 0.03, MeOH); UV (MeOH) λ_max_ (log ε) 203 (3.40), 254 (3.49) nm; IR (KBr) υ_max_ 3417, 2920, 1681, 1634, 1491, 1261 cm^−1^; ^1^H and ^13^C NMR, see Table 6; HRESIMS *m/z* 223.9067[M + H]^+^ (calculated for C_12_H_15_O_4_, 223.0970).

#### 3.2.8. Quadricinctafuran B: (2R)-2-(1,2-Hihydroxypropan-2-yl)-2,3-dihydro-1-benzofuran-5-carboxylic acid (**6b**)

White solid; mp 209–210 °C (CHCl_3_/Me_2_CO); ${\left[\alpha \right]}_{D}^{20}$ +20° (*c* 0.05, MeOH); UV (MeOH) λ_max_ (log ε) 205 (4.27), 261 (4.03) nm; IR (KBr) υ_max_ 3328, 2984, 2941, 1673, 1610, 1599, 1495, 1362, 1290, 1247 cm^−1^; ^1^H and ^13^C NMR, see Table 6; HRESIMS *m/z* 239.0919[M + H]^+^ (calculated for C_12_H_15_O_5_, 239.0919).

#### 3.2.9. Quadricinctone D: 7-Hydroxy-3-(hydroxymethyl)-3-methyl-3,4-dihydro-2-benzoxepine-1, 5-dione (**7**)

White crystals; mp 196–197 °C (CHCl_3_/Me_2_CO); ${\left[\alpha \right]}_{D}^{20}$ +19° (*c* 0.05, MeOH); UV (MeOH) λ_max_ (log ε) 232 (4.21), 254 (3.84) nm; IR (KBr) υ_max_ 3404, 2934, 2360, 2341, 1708, 1670, 1614, 1492, 1466, 1250 cm^−1^; ^1^H and ^13^C NMR, see Table 7; HRESIMS *m/z* 237.0792 [M + H]^+^ (calculated for C_12_H_15_O_5_, 237.0763).

### 3.3. X-Ray Crystal Structure of Compounds **1**, **2a**, **4b**, **5**, **6a** and **7**

Diffraction data were collected at 293 K with a Gemini PX Ultra equipped with CuK_α_ radiation (λ = 1.54184 Å). The structures were solved by direct methods using SHELXS-97 and refined with SHELXL-97 [23]. Carbon, oxygen and sulphur atoms were refined anisotropically. Hydrogen atoms were either placed at their idealized positions using appropriate HFIX instructions in SHELXL and included in subsequent refinement cycles or were directly found from difference Fourier maps and were refined freely with isotropic displacement parameters. Full details of the data collection and refinement and tables of atomic coordinates, bond lengths and angles and torsion angles have been deposited with the Cambridge Crystallographic Data Centre.

Quadricinctone A (**1**): Crystals were triclinic, space group P1, cell volume 663.27(7) Å^3^ and unit cell dimensions *a* = 7.3973(5)Å, *b* =8.4830(6)Å and *c* = 11.2438(6) Å and angles *α* = 107.541(5)°, *β* = 92.005(5)° and *γ* = 98.365(6)° (uncertainties in parentheses). The refinement converged to *R* (all data) = 3.68% and *wR*_2_ (all data) = 9.54%. The absolute structure was established with confidence (flack *x* parameter 0.028(18)). CCDC 1465376.

Quadricinctapyran A (**2a**): Crystals were orthorhombic, space group P*bcn*, cell volume 2189.45(9) Å^3^ and unit cell dimensions *a* = 21.3981(5)Å, *b* = 14.5017(3)Å and *c* = 7.05561(18)Å. The refinement converged to *R* (all data) = 15.06% and *wR*_2_ (all data) = 40.39%. CCDC 1468869.

Quadricinctone B (**4b**): Crystals were orthorhombic, space group I*ba2*, cell volume 2469.66(11) Å^3^ and unit cell dimensions *a* = 8.52510(19)Å, *b* = 30.2340(7)Å and *c* = 9.5817(3)Å. The refinement converged to *R* (all data) = 5.90% and *wR*_2_ (all data) = 15.62%. The absolute structure was established with confidence (flack *x* parameter −0.04(4)). CCDC 1468868.

Quadricinctone C (**5**): Crystals were monoclinic, space group P*2_1_*, cell volume 1116.25(5) Å^3^ and unit cell dimensions *a* = 12.1131(3)Å, *b* = 7.0501(2)Å and *c* = 13.2091(3)Å and *β* = 98.291(2)°. The refinement converged to *R* (all data) = 6.85% and *wR*_2_ (all data) = 17.04%. CCDC 1468170.

Quadricinctafuran A (**6a**): Crystals were monoclinic, space group P*2_1_/n*, cell volume 1066.67(10)Å^3^ and unit cell dimensions *a* = 11.6064(7)Å, *b* = 5.9593(2)Å and *c* = 15.8588(8)Å and *β* = 103.481(6)°. The refinement converged to *R* (all data) = 11.08% and *wR*_2_ (all data) = 24.01%. CCDC 1468171.

Quadricinctone D (**7**): Crystals were orthorhombic, space group P*ca2_1_*, cell volume 1081.35(13) Å^3^ and unit cell dimensions *a* = 19.5607(13)Å, *b* = 7.4646(5)Å and *c* = 7.4058(5)Å. The refinement converged to *R* (all data) = 3.81% and *wR*_2_ (all data) = 8.57%. CCDC 1468166.

### 3.4. Molecular Mechanics Conformation Analysis of **3** and **7**

Molecular simulations for Structures **3** and **7** were carried out in ChemBio3D Ultra 14 (Perkin-Elmer). Molecular mechanics energy minimizations used the MM2 and MMFF force fields with the ChemBio3D most recent default parameters and implementation, adequate for small to medium carbon-based models. Ab initio MP2/6-311G and semi-empirical PM3 molecular modelling was done using CS GAMESS interfaced by ChemBio3D. The conformational search was done by stochastic, dihedral driver and molecular dynamics methods. Around 60 conformations were stochastically created and then minimized using the PM3, MMFF and PM3 methods. The resulting models were then grouped under the resultant two seven-membered ring conformations (exemplified in Figure 8 for **3**). A minimal energy for each model set was found by driving by 360° the dihedral angles of the single bonds that attach substituent groups to the rings. Molecular dynamics runs were also applied to the models to confirm the convergence to a minimal energy conformation for each model set.

## 4. Conclusions

Although Ozoe et al. had first reported the isolation of an isocoumarin derivative from the culture of the fungus *N. quadricincta* strain PF1223, using a GABA receptor ligand as a screening target, the source of the fungus was not revealed. Therefore, this is the first report on the secondary metabolites of the culture of a marine-derived *N. quadricincta* (KUFA 0081). Among the nine new isolated compounds, two are polyketide derivatives, and seven are benzoic acid derivatives. The isolation of these structurally-unique, but related, metabolites from this fungus has allowed us to propose the biosynthetic pathways leading to these metabolites. Interestingly, the unprecedented structure of quadricinctone B (**4b**), which possesses a methyl sulfinyl group in the benzopyran nucleus, reflects the capacity of this marine-derived strain to introduce sulphur into the aromatic ring. Therefore, this marine strain of *N. quadricincta* (KUFA 0081) can have potential for biotechnological transformation. Even though the isolated metabolites did not exhibit either antifungal/antibacterial activities or growth inhibitory activity against the three cancer cell lines in our assay protocols, it does not mean that they are devoid of other interesting biological activities. Therefore, it is necessary to investigate these metabolites in other target-based assay protocols.

## Figures and Tables

**Table 1:** ^1^H and ^13^C NMR (CDCl_3_, 300.13 MHz and 75.4 MHz), HMBC and NOESY assignments for **1**.

Position	δ_C_, type	δ_H_, (*J* in Hz)	HMBC	NOESY
1	168.2, CO	-		
3	88.8, C	-		
3a	152.8, C	-		
4	111.7, C	-		
5	164.6, C	-		
6	94.5, CH	6.41, s	C-1, 4, 5, 7, 7a	OMe-5, 7
7	158.3, C	-		
7a	105.3, C	-		
8	11.2, CH_3_	2.12, s	C-3a, 4, 5, 7	H-9, 10
9	21.5, CH_3_	1.76, s	C-3, 3a, 10	H-8, 9
10	70.8, CH	4.22, d (6.4)	C-11	H-8, 9, 10
11	17.8, CH_3_	0.87, d (6.4)	C-3, 10	H-10
OMe-5	56.1, CH_3_	3.92, s	C-5	H-6
OMe-7	56.0, CH_3_	3.97, s	C-7	H-6
OH-10	-	2.15, br	-	H-11

**Table 2:** ^1^H and ^13^C NMR (DMSO, 300.13 MHz and 75.4 MHz) for **2a** and **2b**.

Position	2a					2b			
	δ_C_, type	δ_H_, (*J* in Hz)	COSY	HMBC		δ_C_, type	δ_H_, (*J* in Hz)	COSY	HMBC
2	80.5, C	-				78.5, C	-		
3	128.6, CH	5.74, d (10.0)	H-4	C-2, 4a, 5, 9, 10		126.2, CH	5.59, d (10.0)	H-4	C-2, 4a, 10
4	122.8, CH	6.56, d (10.0)	H-3	C-2, 5, 8a		124.3, CH	6.51, d (10.0)	H-3	C-2, 5, 8a
4a	120.5, C	-				120.2, C	-		
5	127.9, CH	7.65, d (2.1)	H-7	C-4, 8a, 11		128.9, CH	7.75, d (2.1)	H-7	C-4, 7, 8a, 11
6	123.0, C	-				122.0, C	-		
7	139.8, CH	7.69, dd (8.4, 2.1)	H-5, 8	C-5, 8a, 11		132.2, CH	7.90, dd (8.5, 2.1)	H-5, 8	C-5, 8a, 11
8	115.7, CH	6.79, d (8.4)	H-7	C-4a, 6, 8a		116.1, CH	6.83, d (8.5)	H-7	C-4a, 6, 8a
8a	156.8, C	-				157.8, C	-		
9	23.3, CH_3_	1.31, s	-	C-2, 3, 10		24.0, CH_3_	1.48, s	-	C-2, 3, 10
10	67.1, CH_2_	3.45, s	-	C-2, 3, 9		68.8, CH_2_	4.13, d (11.7)	H-10	C-2, 3, 9
							4.24, d (11.7)	H-10	C-2, 3, 9
11	167.0, CO	-				171.7, CO	-		
OH-10	-	5.07, br	-			-	-		
OH-11	-	12.59, br	-			-	12.79, br	-	
OAc	-					170.8, CO	-		
						20.7, CH_3_	1.98, s	-	CO (Ac)

**Table 3:** ^1^H and ^13^C NMR (DMSO, 300.13 MHz and 75.4 MHz) and HMBC assignment for **3**.

Position	δ_C_, type	δ_H_, (*J* in Hz)	COSY	HMBC	NOESY
2α	77.0, CH_2_	3.84, d (11.1)	H-2β	C-3, 4, 9a, 11	H-2β, 4 (w), 11 (w)
β		4.02, dd (11.1, 1.6)	H-2α	C-3, 4, 9a, 11	H-2α, 4 (w), 11 (str)
3	70.7, C	-			
4	139.5, CH	5.95, dd (12.0, 1.2)	H-5	C-2, 5a	H-2α (w), 5, 9 (w), 11
5	123.5, CH	6.31, d (12.0)	H-4	C-3, 5a, 6, 9a	H-4, 6 (str)
5a	125.0, C	-			
6	134.5, CH	7.89, d (2.1)	H-8	C-5, 8, 9a, 10	H-5
7	125.3, C	-			
8	129.8, CH	7.74, dd (8.4, 2.1)	H-6, 9	C-6, 9a	H-9
9	119.8, CH	7.05, d (8.4)	H-8	C-5a, 7, 9a	H-8
9a	161.2, C	-			
10	166.7, C	-			
11	26.1, CH_3_	1.26, s	-	C-2, 3, 4	H-2α (w), 2β (str)
OH-3	-	3.39, br	-		H-4, 11
COOH	-	12.79, br	-		

**Table 4:** ^1^H and ^13^C NMR (DMSO, 300.13 MHz and 75.4 MHz) and HMBC assignment for **4b**.

Position	δ_C_, type	δ_H_, (*J* in Hz)	COSY	HMBC
2	80.9, C	-		
3	48.0, CH_2_	2.80, d (16.6)	H-3	C-2, 4, 9, 10
		2.85, d (16.6)	H-3	C-2, 4, 9, 10
4	191.2, CO	-		
4a	120.8, C	-		
5	112.4, CH	7.17, d (3.1)	H-7	C-4, 6, 7, 8a
6	151.6, C	-		
7	118.7, CH	7.35, d (3.1)	H-5	C-5, 6, 8, 8a
8	135.7, C	-		
8a	147.4, C	-		
9	26.1, CH_3_	1.39, s	-	C-2, 3, 10
10	25.7, CH_3_	1.38, s	-	C-2, 3, 9
11	40.8, CH_3_	2.77, s	-	C-8
OH-6	-	9.85, s		C-5, 6, 7

**Table 5:** ^1^H and ^13^C NMR (DMSO, 300.13 MHz and 75.4 MHz) and HMBC assignment for **5**.

Position	δ_C_, type	δ_H_, (*J* in Hz)	COSY	HMBC
1	168.8, C	-		
3	104.8, C	-		
4	34.9, CH	3.26, m	H_3_-10	
4a	144.6, C	-		
5	113.5, C	-		
6	162.9, C	-		
7	99.9, CH	6.27, s	-	C-1 (w), 5, 6, 8, 8a
8	161.3, C	-		
8a	98.9, C	-		
9	63.7, CH_2_	3.65, brd (16.6)	OH-9	
10	16.3, CH_3_	1.06, d (7.2)	H-4	C-3, 4, 4a
11	9.8, CH_3_	1.98, s	-	C-4a, 5, 6
OH-3	-	7.07, br	-	
OH-5	-	10.62, br	-	
OH-8	-	11.24, s	-	C-7, 8, 8a
OH-9	-	5.26, br	-	

**Table 6:** ^1^H and ^13^C NMR (DMSO, 300.13 MHz and 75.4 MHz) and HMBC assignment for **6a** and **6b**.

Position	6a					6b			
	δ_C_, type	δ_H_, (*J* in Hz)	COSY	HMBC		δ_C_, type	δ_H_, (*J* in Hz)	COSY	HMBC
2	90.2, CH	4.64, t (8.9)	H-3	C-1′, 2′, 3′		86.5, CH	4.83, dd (9.7, 8.1)	H-3	
3	29.3, CH_2_	3.17, d (8.9)	H-2	C-1′, 2, 3a, 7a		28.7, CH_2_	3.19, m	H-2	C-1′, 3a
3a	128.2, C	-				128.4, C	-		
4	126.4, CH	7.75, d (1.8)	H-6	C-6, 7a, 8		126.4, CH	7.76, d (1.8)	H-6	C-3, 6, 7a
5	122.6, C	-				122.6, C	-		
6	130.4, CH	7.72, dd (8.3, 1.8)	H-4, 7	C-4, 7a, 8		130.8, CH	7.71, dd (8.3, 1.8)	H-4, 7	C-4, 7a, 8
7	108.4, CH	6.80, d (8.3)	H-6	C-3a, 5, 7a		108.4, CH	6.79, d (8.3)	H-6	C-3a, 5, 7a
7a	163.7, C	-				163.6, C	-		
8	167.2, C	-				167.2, C	-		
1′	70.0, C	-				72.5, C	-		
2′	24.9, CH_3_	1.14, s	-	C-1′, 2, 3′		66.7, CH_2_	3.33, s	-	C-1′, 2, 3′
3′	25.9, CH_3_	1.13, s	-	C-1′, 2, 2′		20.0, CH_3_	1.09, s		C-1′, 2, 2′
OH-8	-	12.49, br	-			-	12.47, br	-	

**Table 7:** ^1^H and ^13^C NMR (CDCl_3_, 300.13 MHz and 75.47 MHz) and HMBC assignment for **7**.

Position	δ_C_, Type	δ_H_, (*J* in Hz)	COSY	HMBC	NOESY
2	80.3, C	-			
3α	434, CH_2_	2.74, d (16.7)	H-3	C-2, 4, 10, 11	H_3_-11 (str)
β		3.00, d (16.7)	H-3	C-2, 4, 10, 11	H_3_-11 (w)
4	191.1, CO	-			
4a	123.0, C	-			
5	127.5, CH	8.26, d (2.3)	H-7	C-4, 7, 8a	
6	119.6, C	-			
7	136.4, CH	8.04, dd (8.7, 2.3)	H-5, 8	C-5, 8a	
8	118.5, CH	7.07, d (8.7)	H-7	C-4a, 6, 8	
8a	162.9, C	-			
9	21.2, CH_3_	1.30, s	-	C-2, 3, 10	H-3α (str), H3β (w), H-5, 8 (w)
10	66.8, CH_2_	3.49, dd (11.6, 4.5)	OH-10		
		3.59, dd (11.6, 4.2)			
11	166.4, CO	-			
OH-10	-	5.26, brt (5.4)	-		
OH-11	-	12.69, br	-		

## Supplementary Materials

The supplementary materials are available online at www.mdpi.com/1660-3397/14/7/134/s1.
