# Supplementary material for: New Polyketides and New Benzoic Acid Derivatives from the Marine Sponge-Associated Fungus Neosartorya quadricincta KUFA 0081
Source: Mar Drugs. 2016 Jul 16;14(7):134. doi: 10.3390/md14070134 (PMC4962024; doi:10.3390/md14070134)
Supplement: Supplementary file 1 [file marinedrugs-14-00134-s001.pdf]

# Supplementary Materials: New Polyketides and New Benzoic Acid Derivatives from the Marine Sponge-Associated Fungus *Neosartorya quadricincta* KUFA 0081

Chadaporn Prompanya [1,2], Tida Dethoup [3], Luís Gales [1,4], Michael Lee [5], José A. C. Pereira [1], Artur M. S. Silva [6], Madalena M. M. Pinto [2,7], Anake Kijjoa [1,2,*]

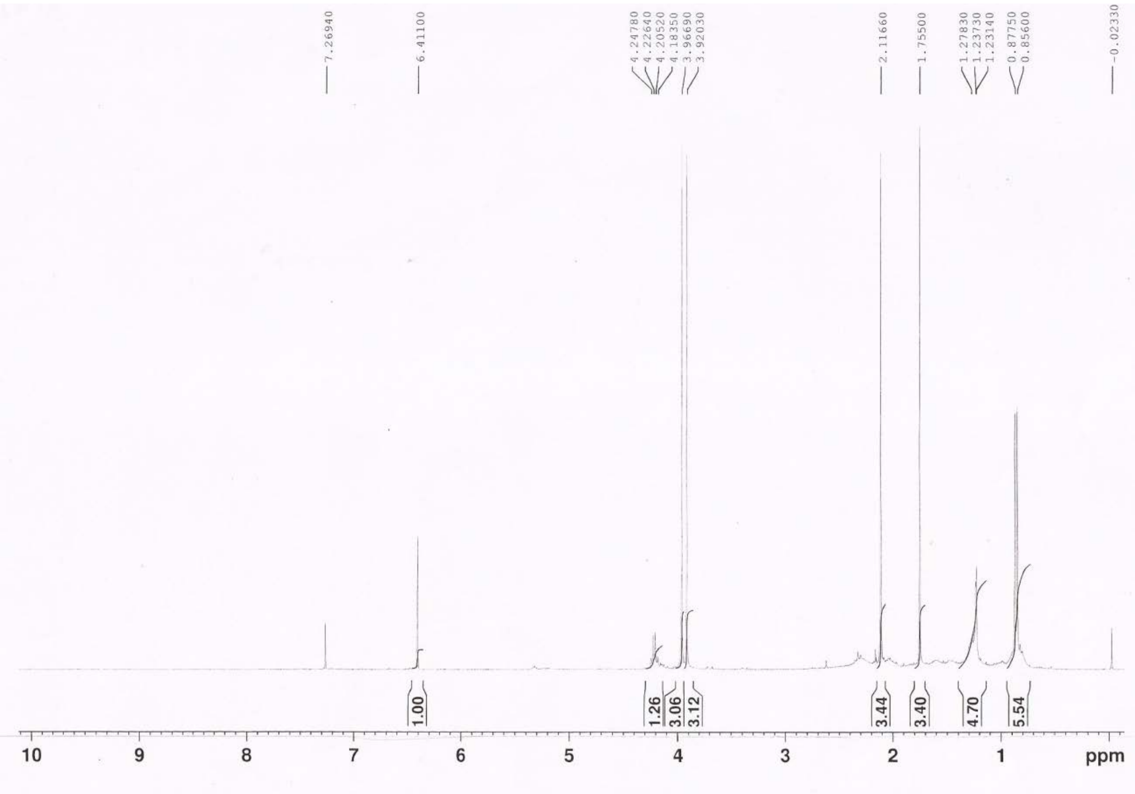


**Figure S1**. $^{1}$H NMR spectrum of compound **1** ($CDCl_3$, 300.13 MHz).

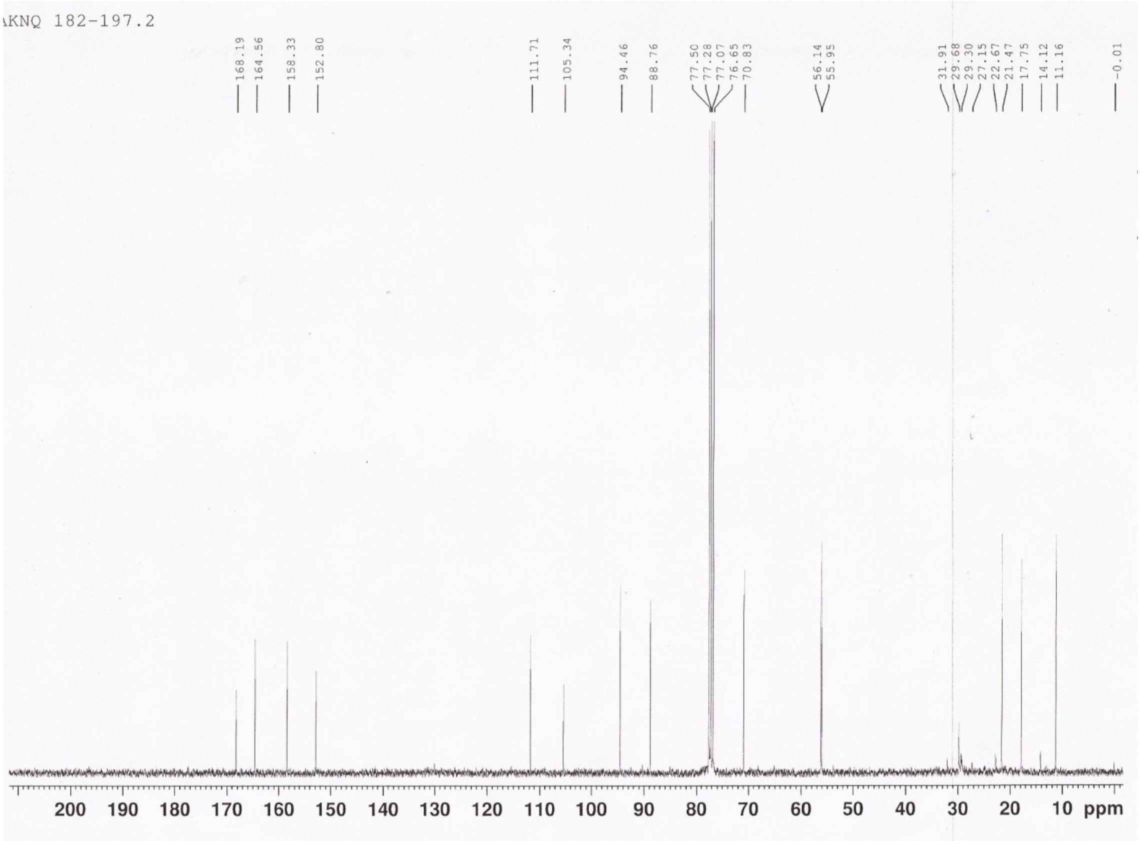


**Figure S2.** $^{13}$C NMR spectrum of compound **1** ($CDCl_3$, 75.47 MHz).

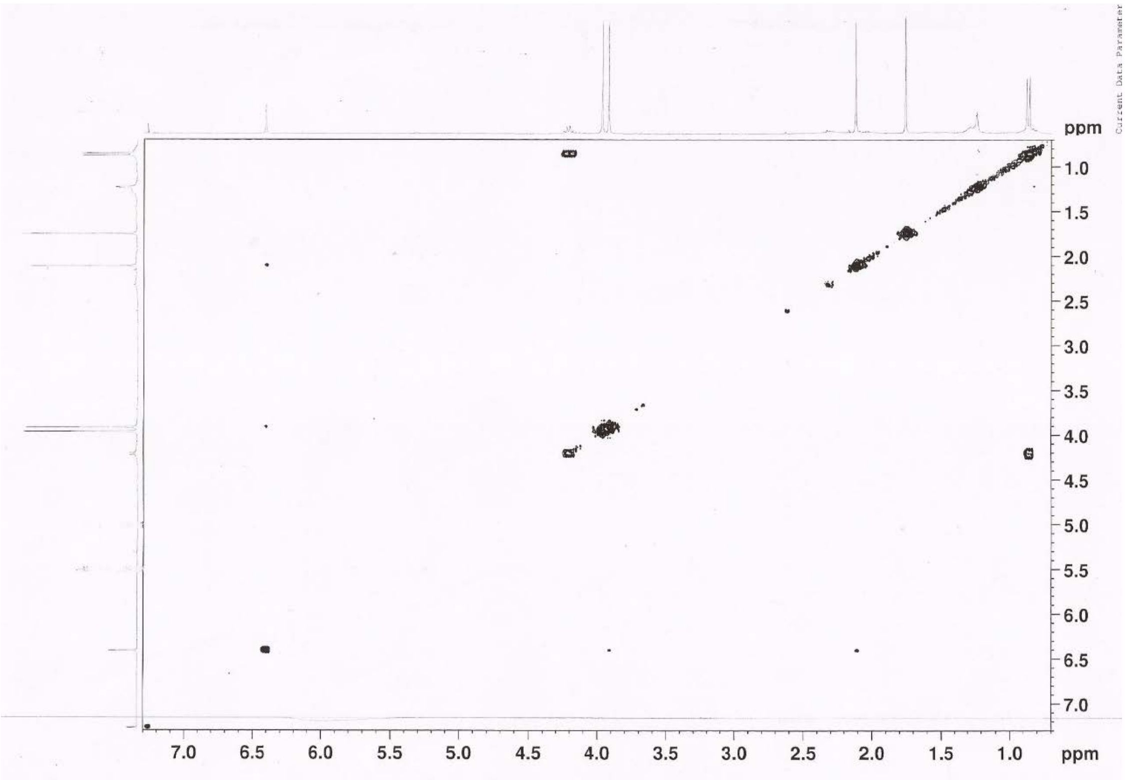


**Figure S3.** COSY spectrum of compound **1** ($CDCl_3$, 300.13 MHz).

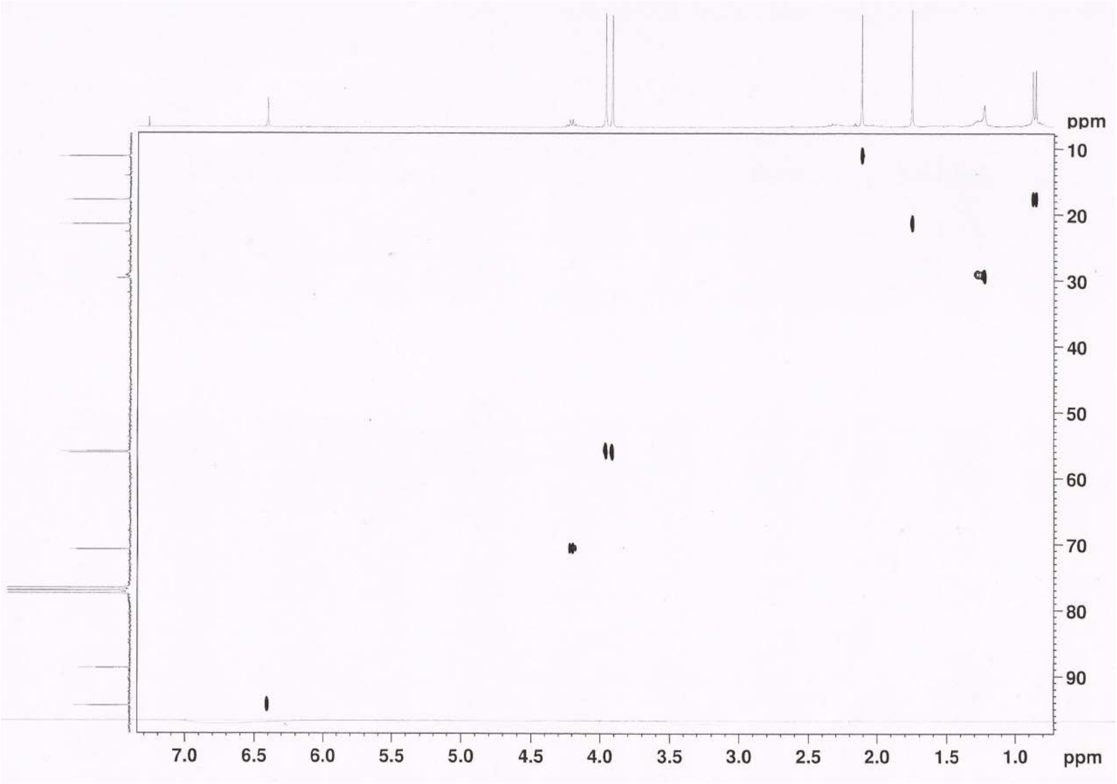


**Figure S4.** HSQC spectrum of compound **1** ($CDCl_3$, 300.13 MHz).

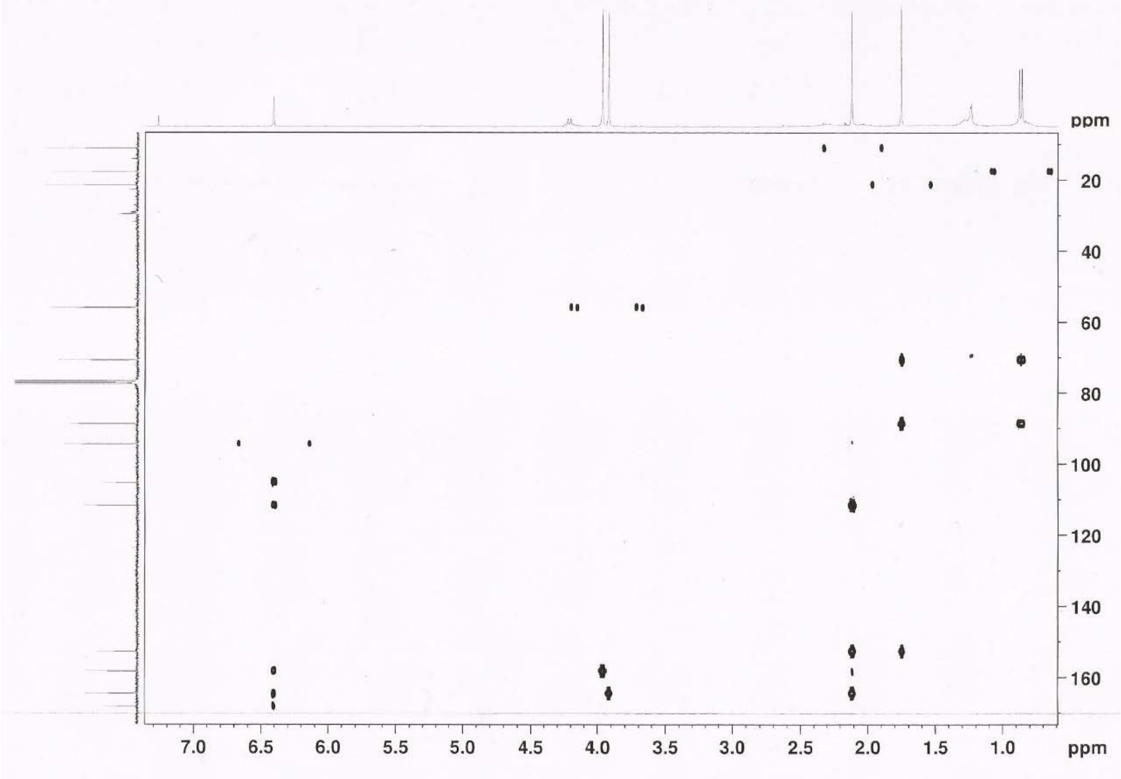


**Figure S5.** HMBC spectrum of compound **1** ($CDCl_3$, 300.13 MHz).

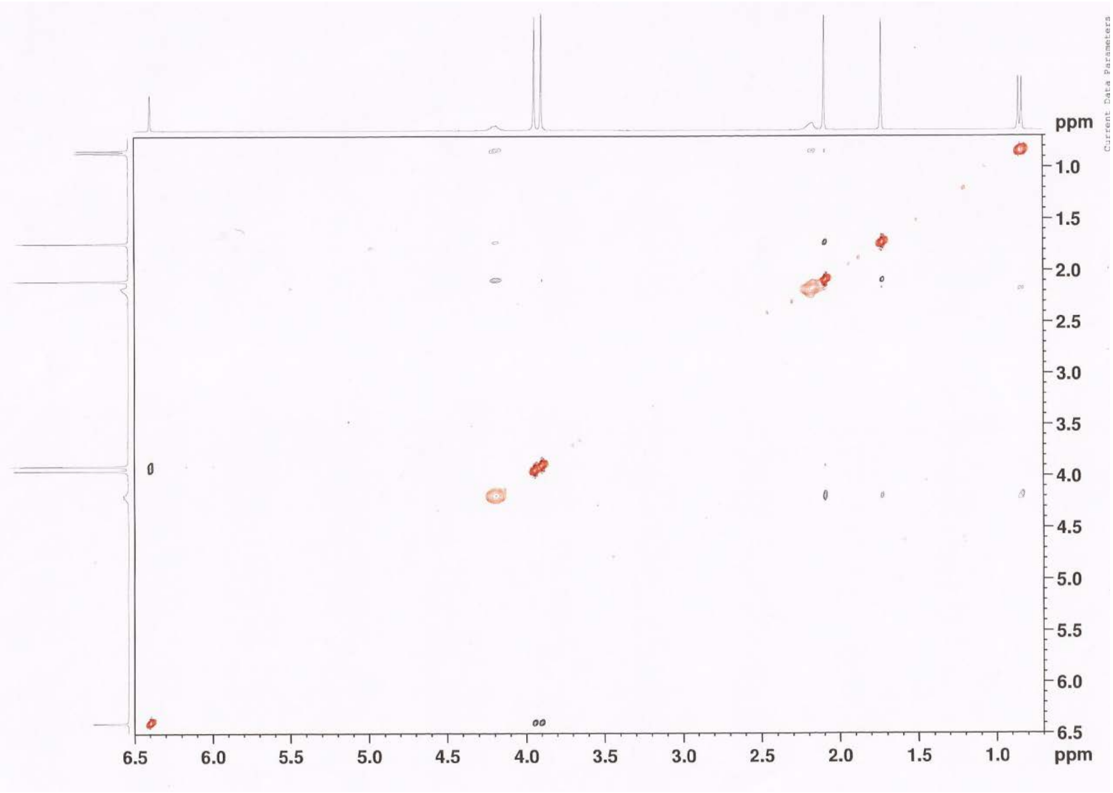


**Figure S6.** NOESY spectrum of compound **1** ($CDCl_3$, 300.13 MHz).

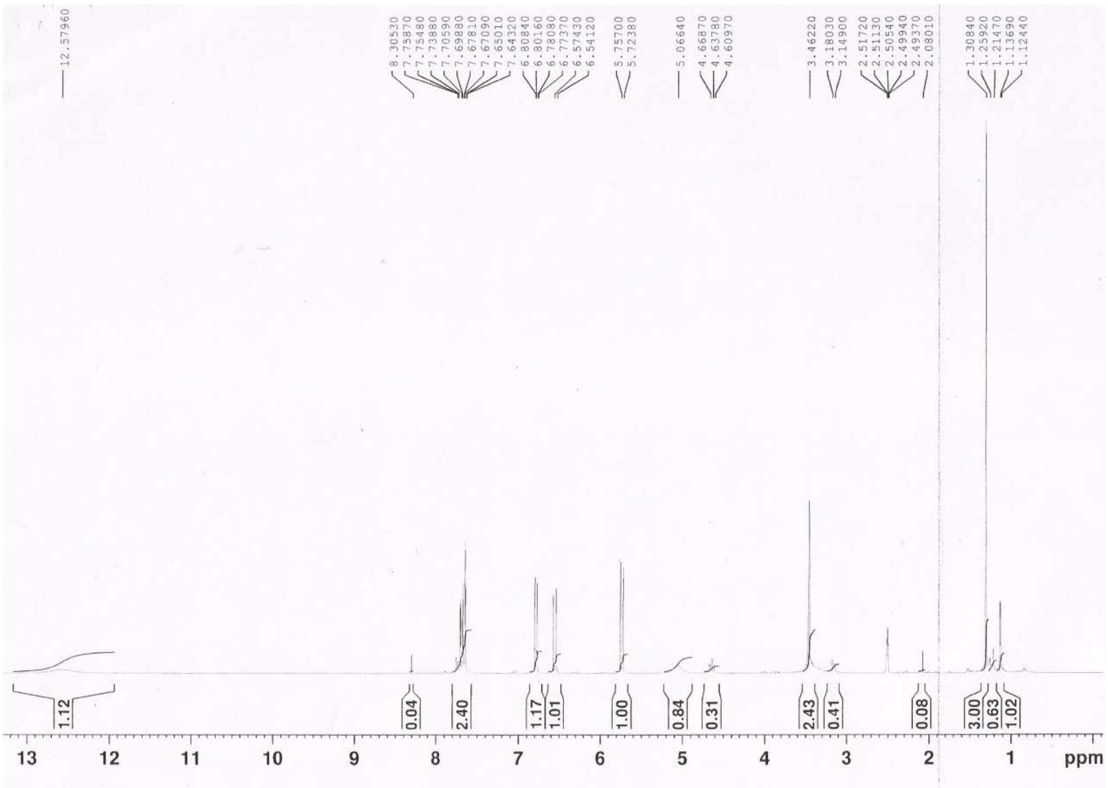


**Figure S7.** $^{1}$H NMR spectrum of compound **2a** (DMSO, 300.13 MHz).

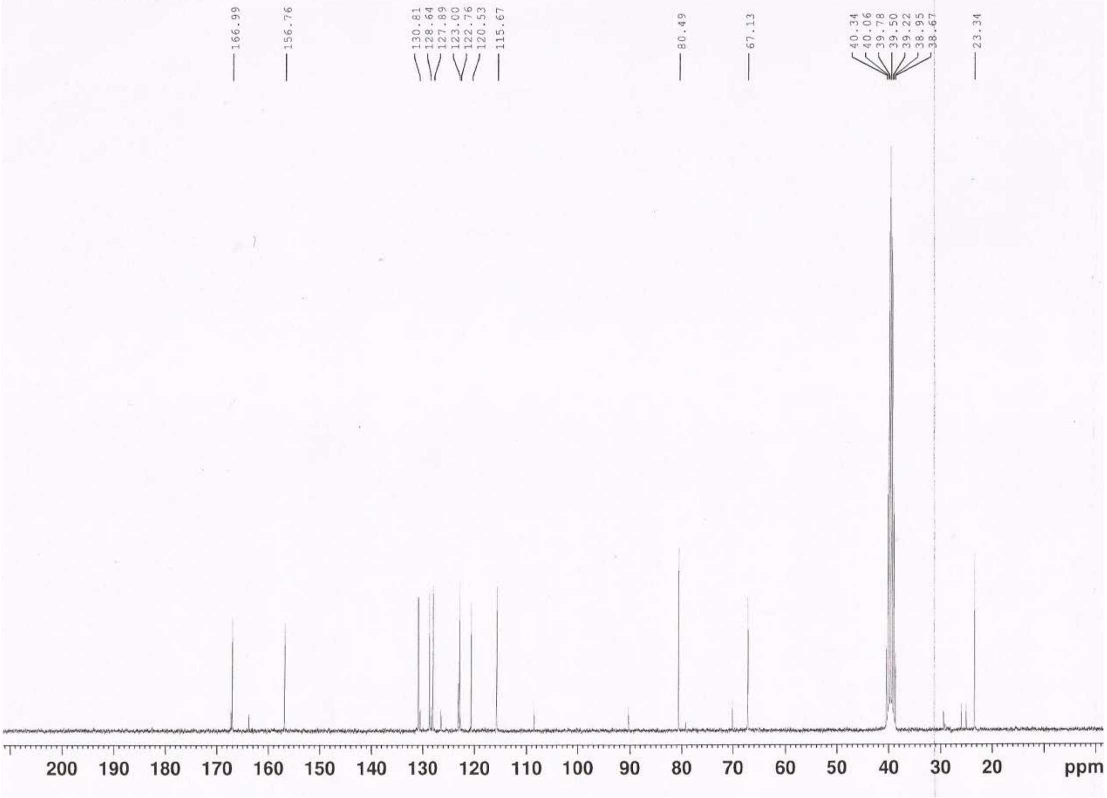


**Figure S8.** $^{13}C$ NMR spectrum of compound **2a** (DMSO, 75.4 MHz).

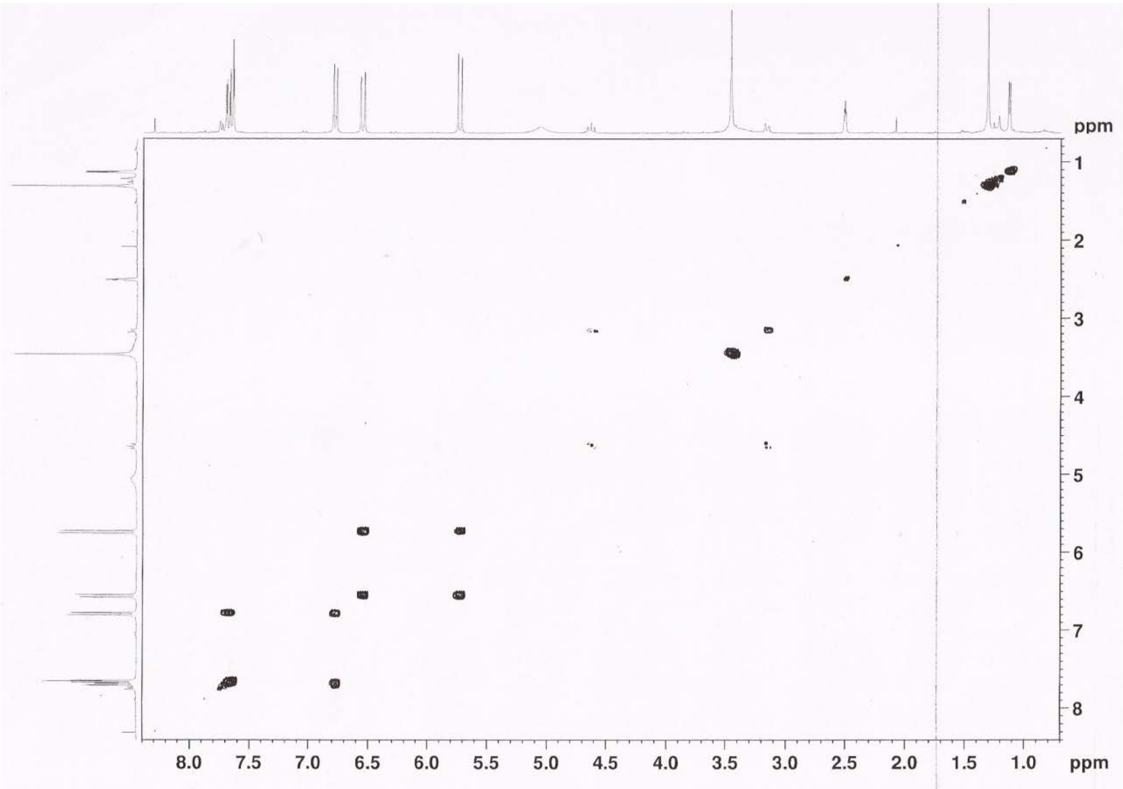


**Figure S9.** COSY spectrum of compound **2a** (DMSO, 300.13 MHz).

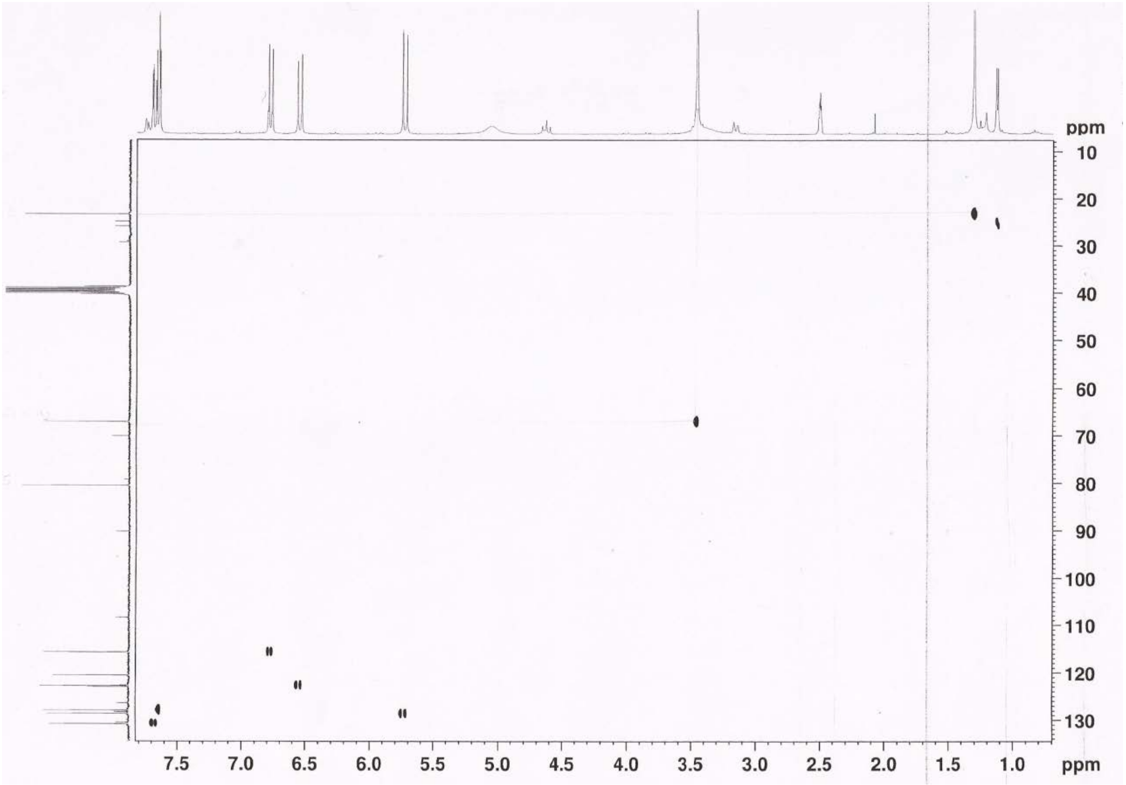


**Figure S10.** HSQC spectrum of compound **2a** (DMSO, 300.13 MHz).

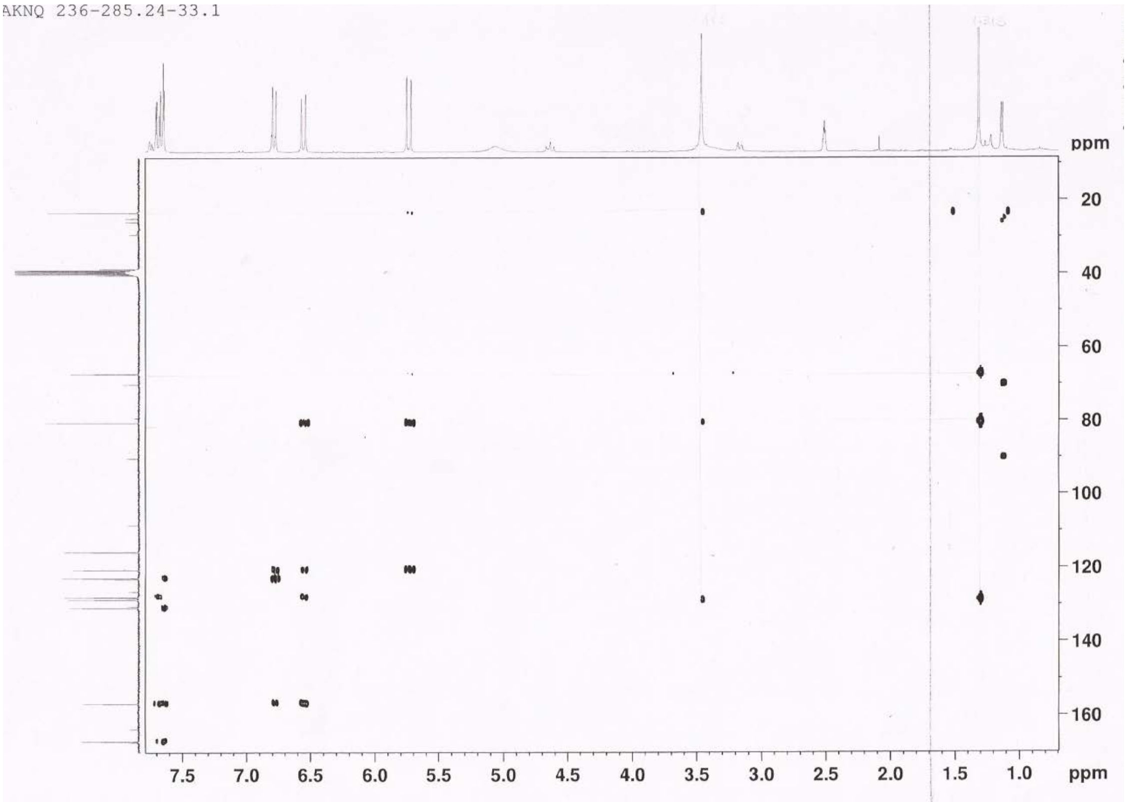


**Figure S11.** HMBC spectrum of compound **2a** (DMSO, 300.13 MHz).

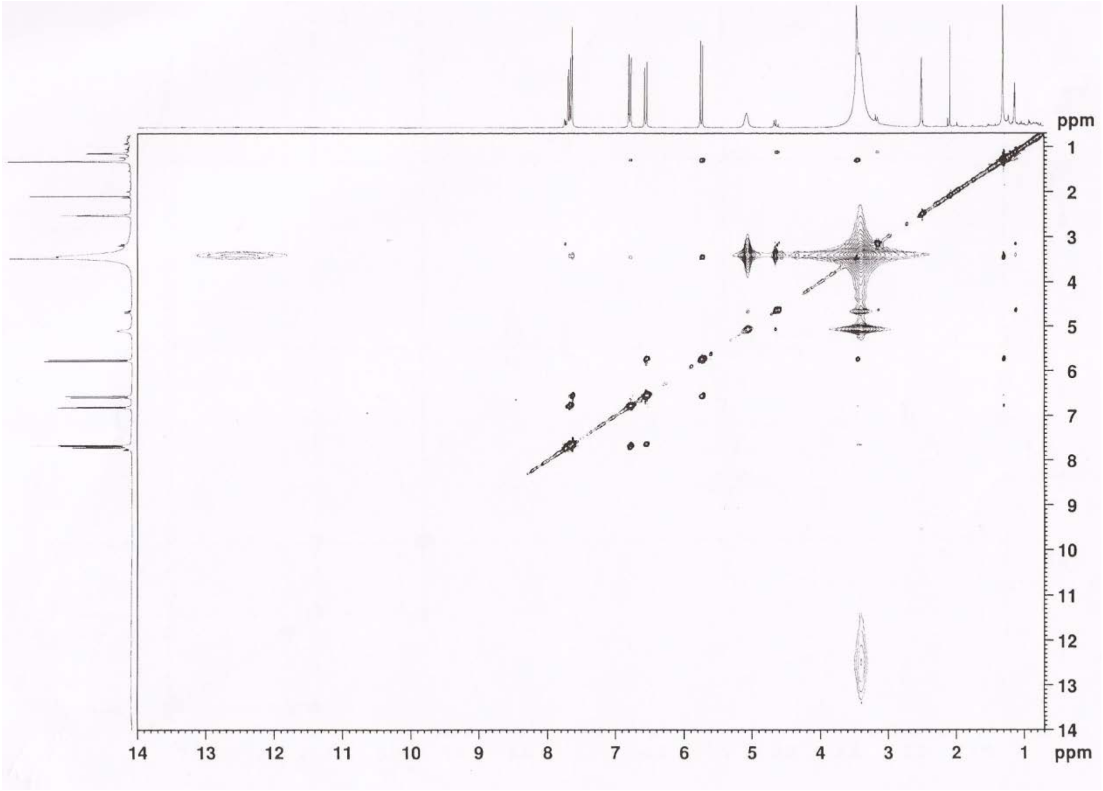


**Figure S12.** NOESY spectrum of compound **2a** (DMSO, 300.13 MHz).

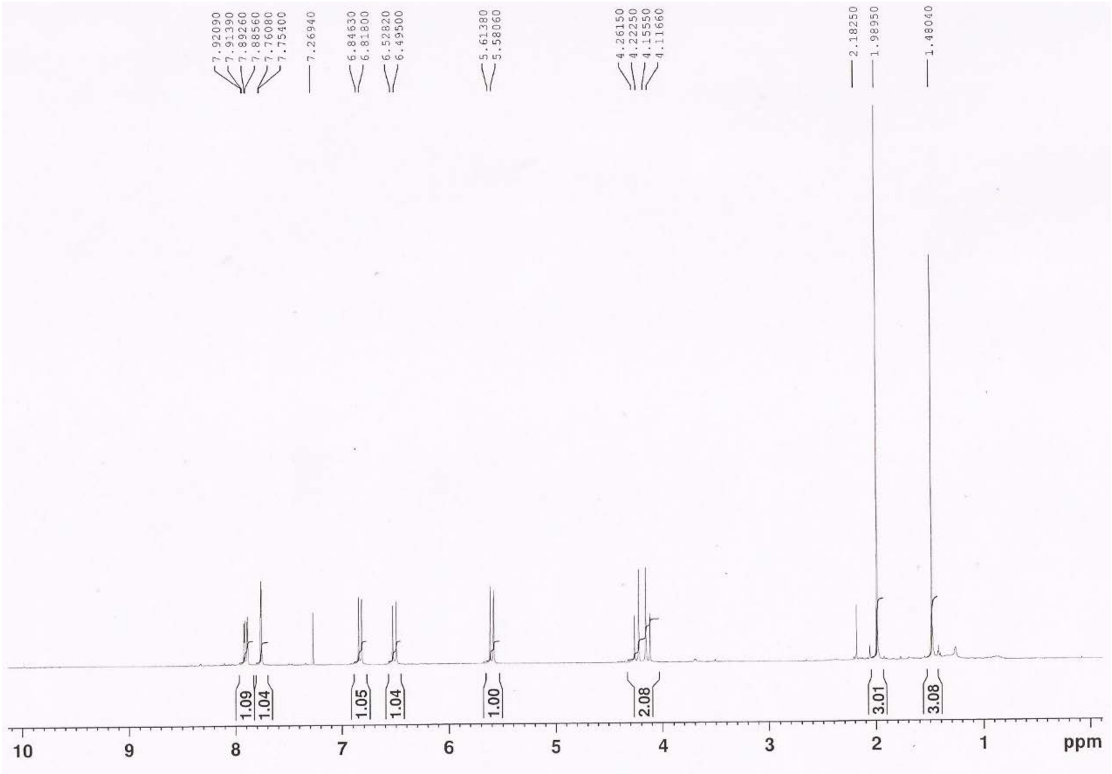


**Figure S13.** $^{1}$H NMR spectrum of compound **2b** (DMSO, 300.13 MHz).

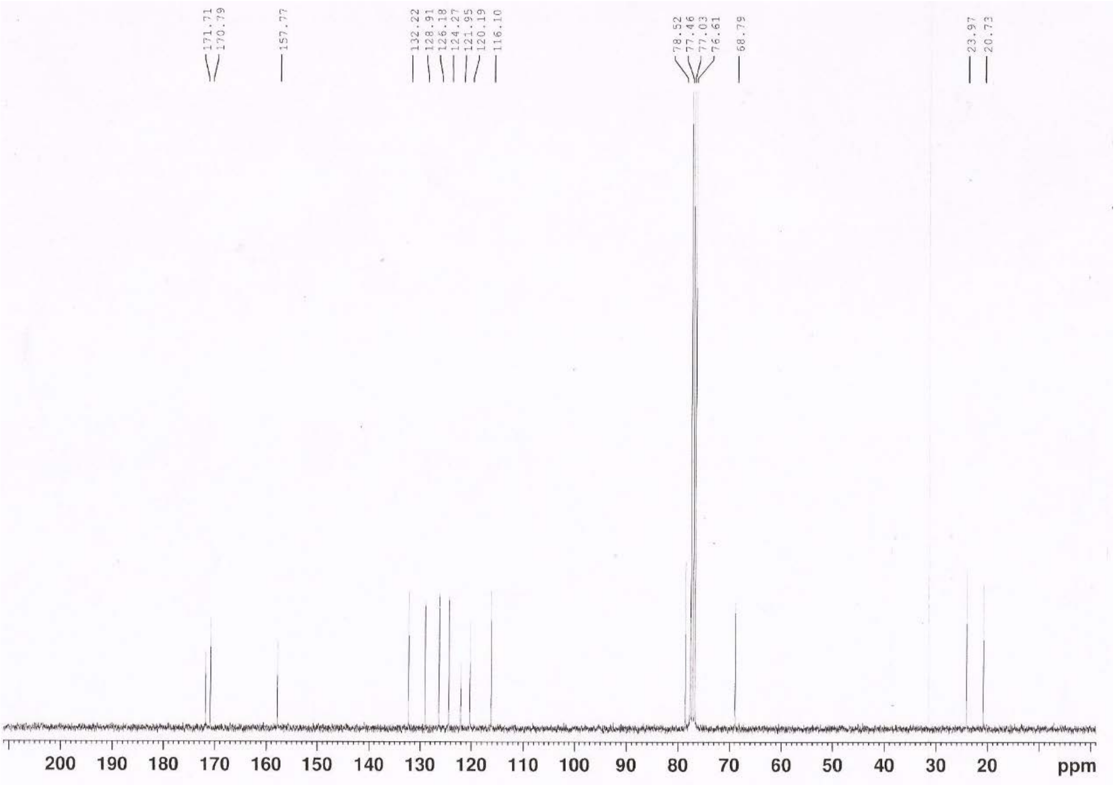


**Figure S14.** $^{13}C$ NMR spectrum of compound **2a** (DMSO, 75.4 MHz).

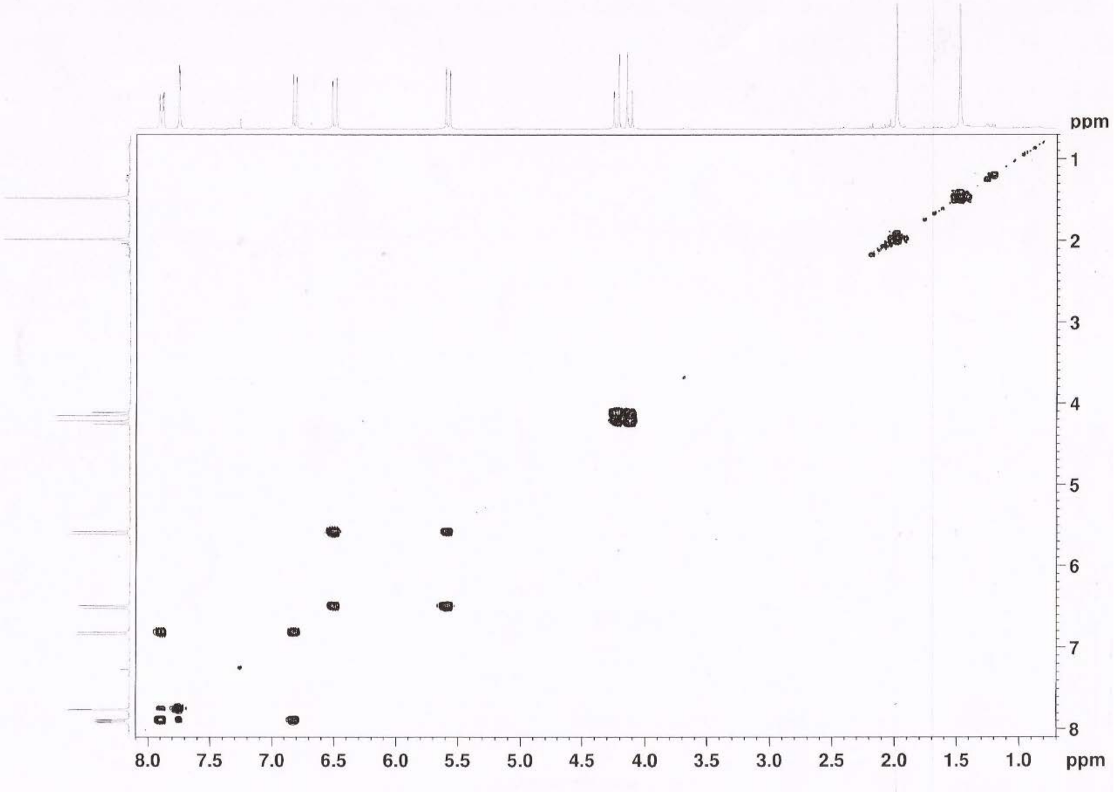


**Figure S15.** COSY spectrum of compound **2b** (DMSO, 300.13 MHz).

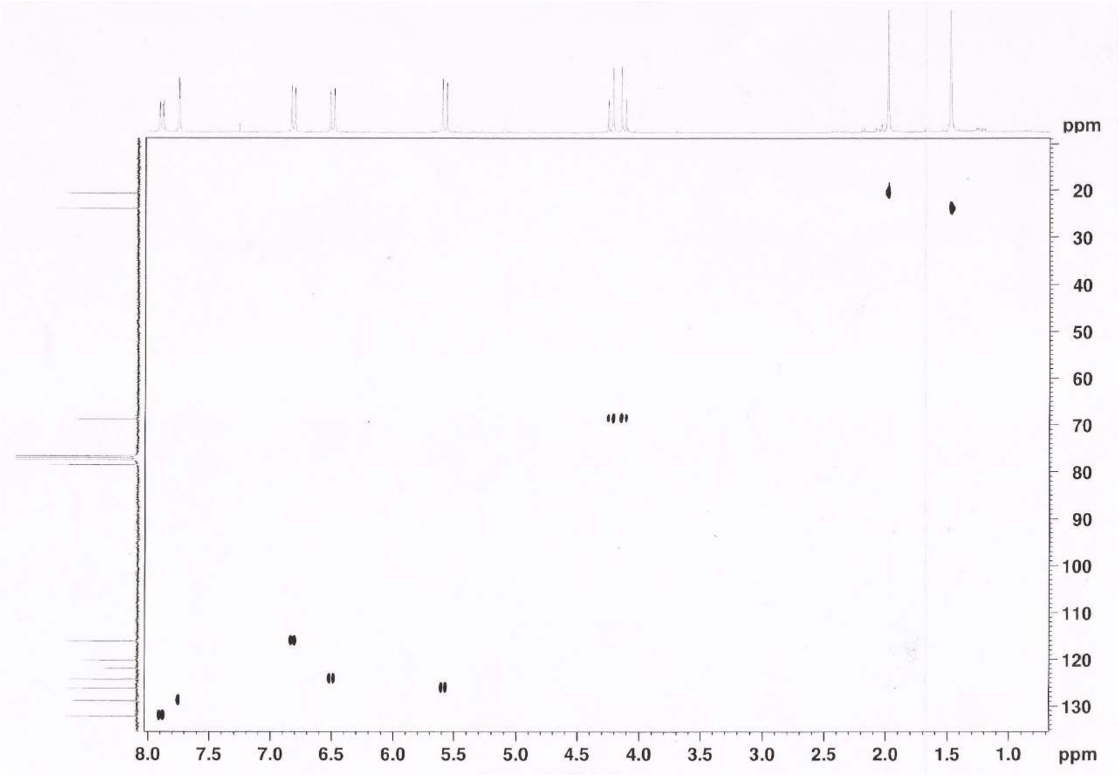


**Figure S16.** HSQC spectrum of compound **2b** (DMSO, 300.13 MHz).

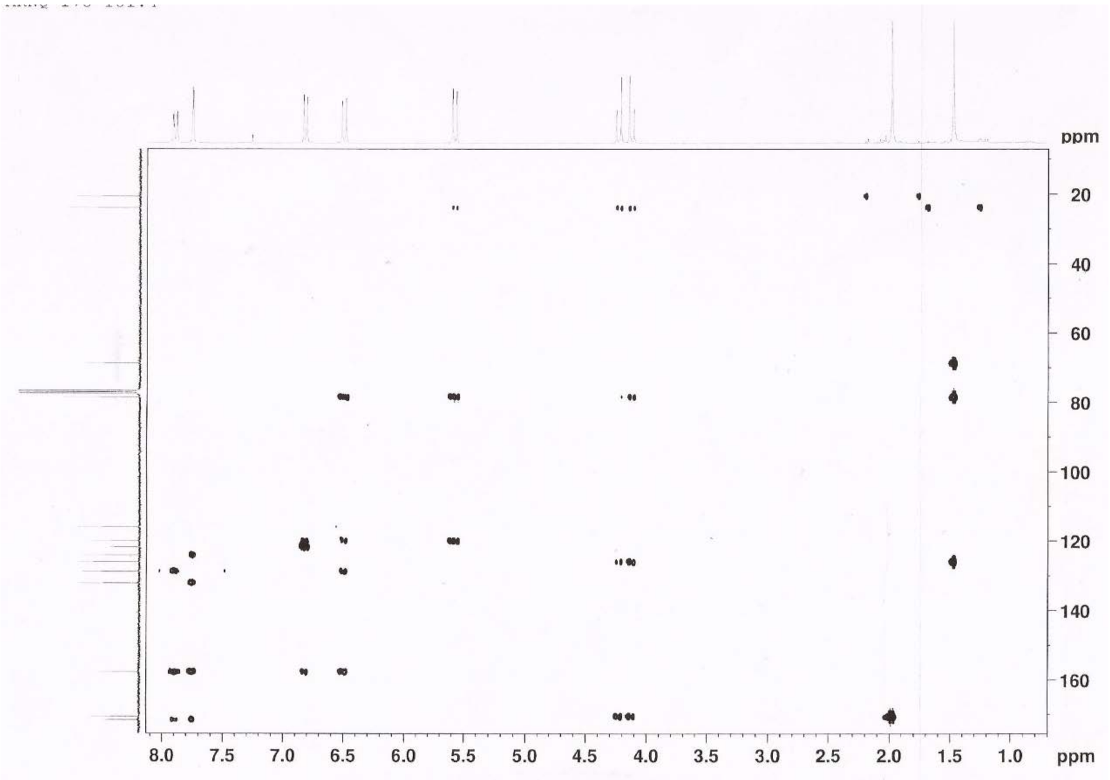


**Figure S17.** HMBC spectrum of compound **2b** (DMSO, 300.13 MHz).

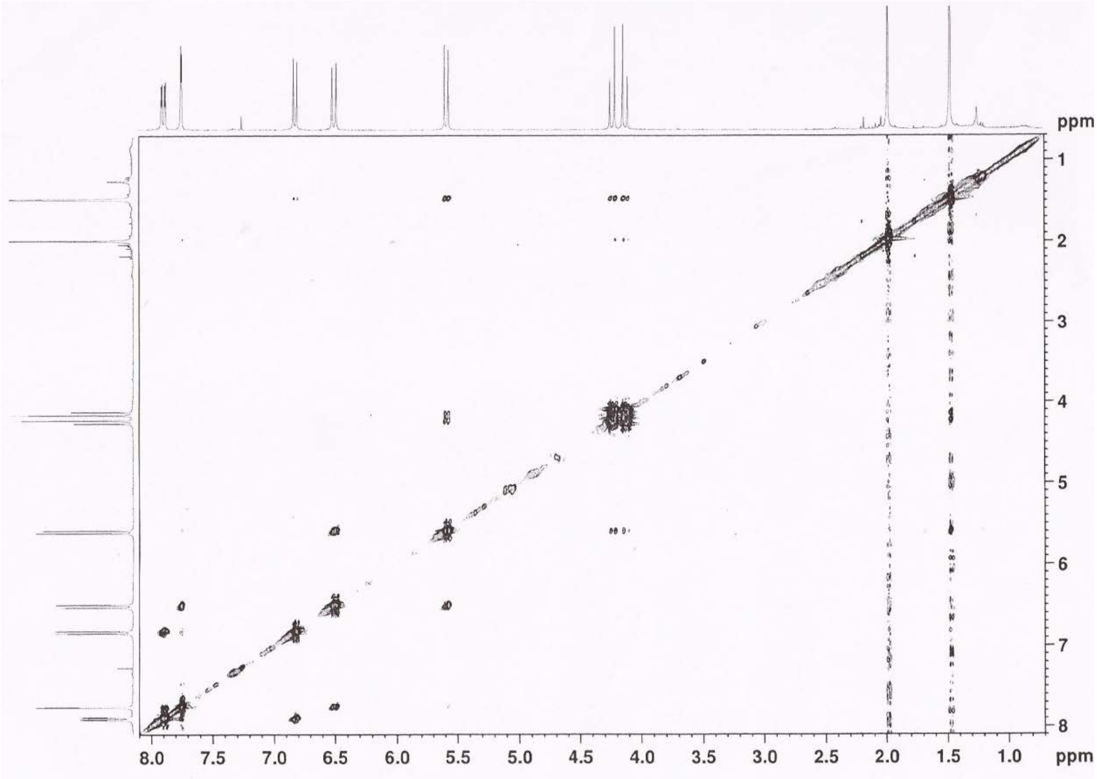


**Figure S18.** NOESY spectrum of compound **2b** (DMSO, 300.13 MHz).

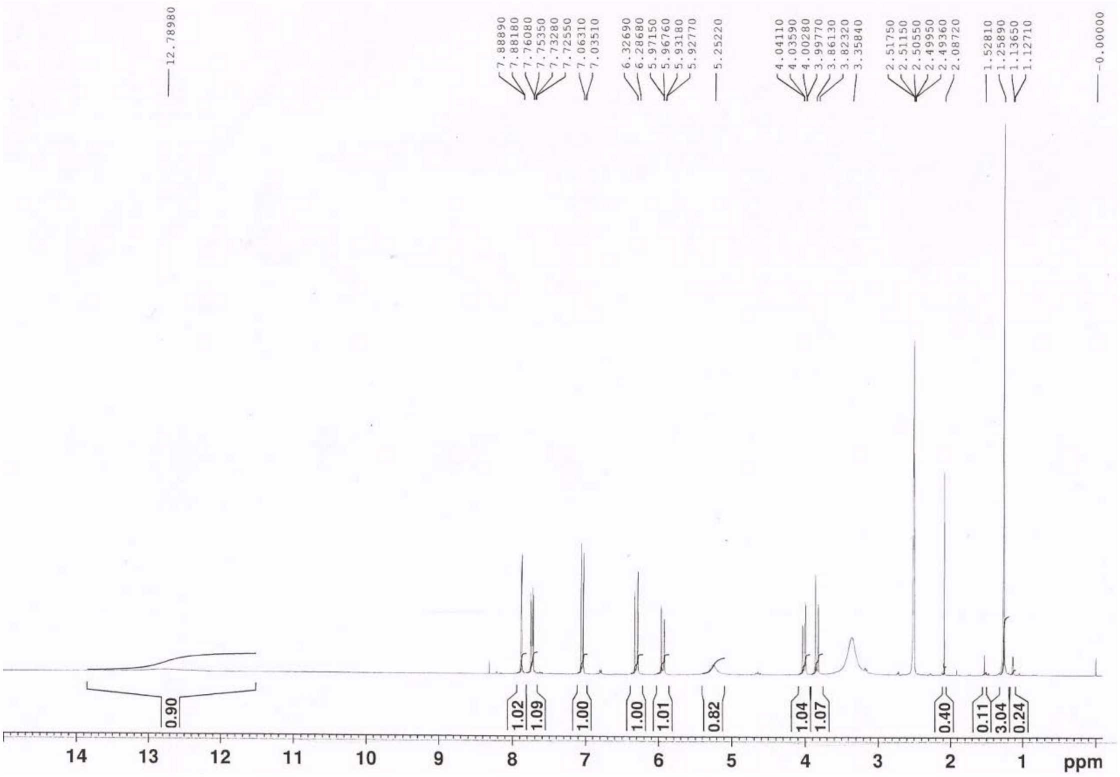


**Figure S19.** $^{1}$H NMR spectrum of compound **3** (DMSO, 300.13 MHz).

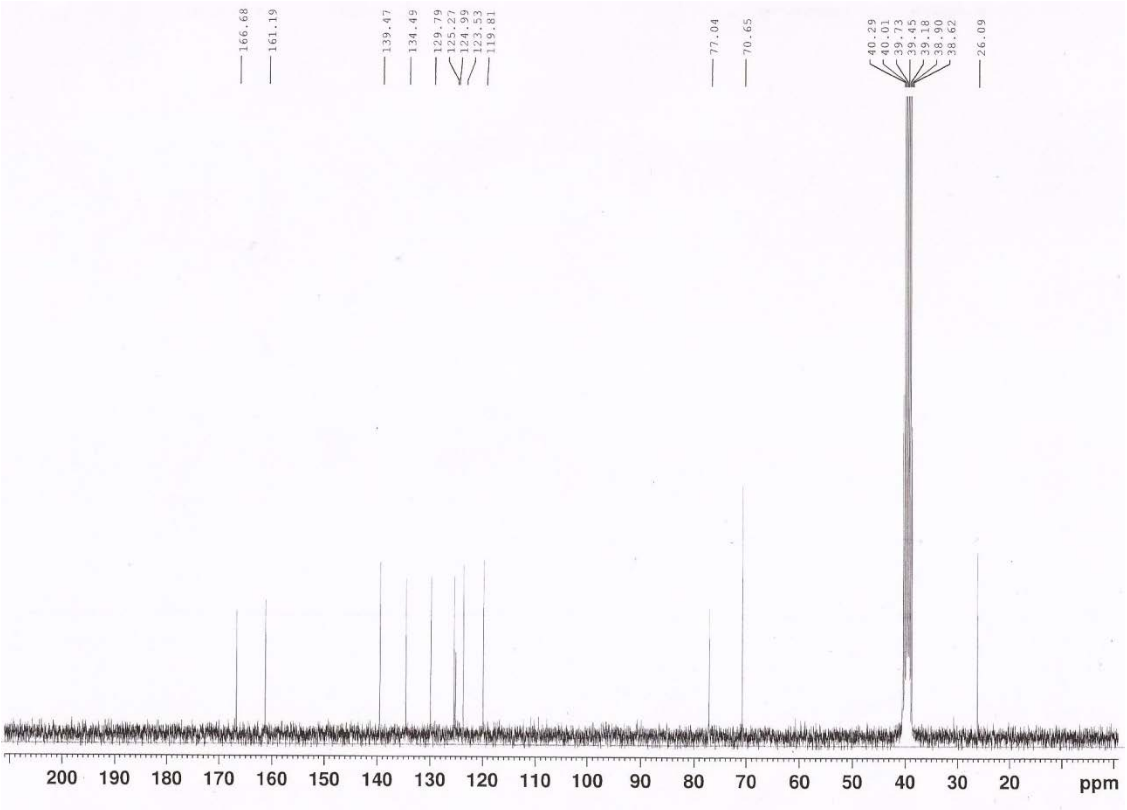


**Figure S20.** $^{13}C$ NMR spectrum of compound **3** (DMSO, 75.4 MHz).

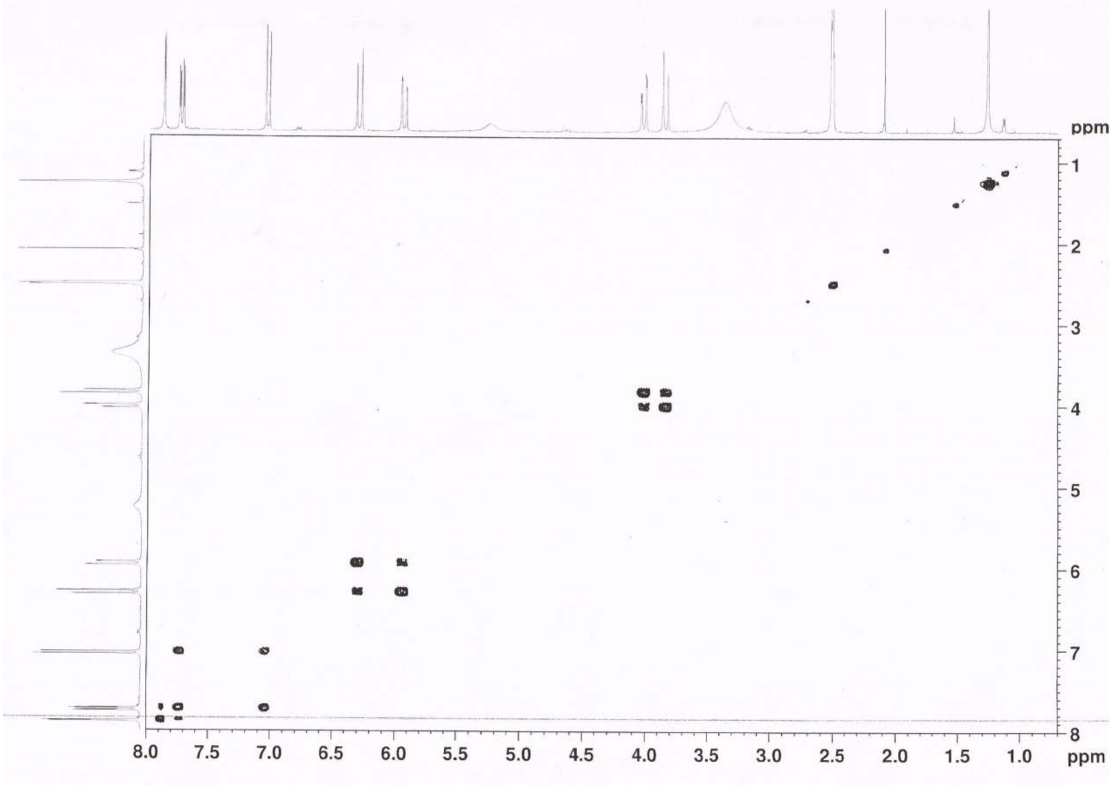


**Figure S21.** COSY spectrum of compound **3** (DMSO, 300.13 MHz).

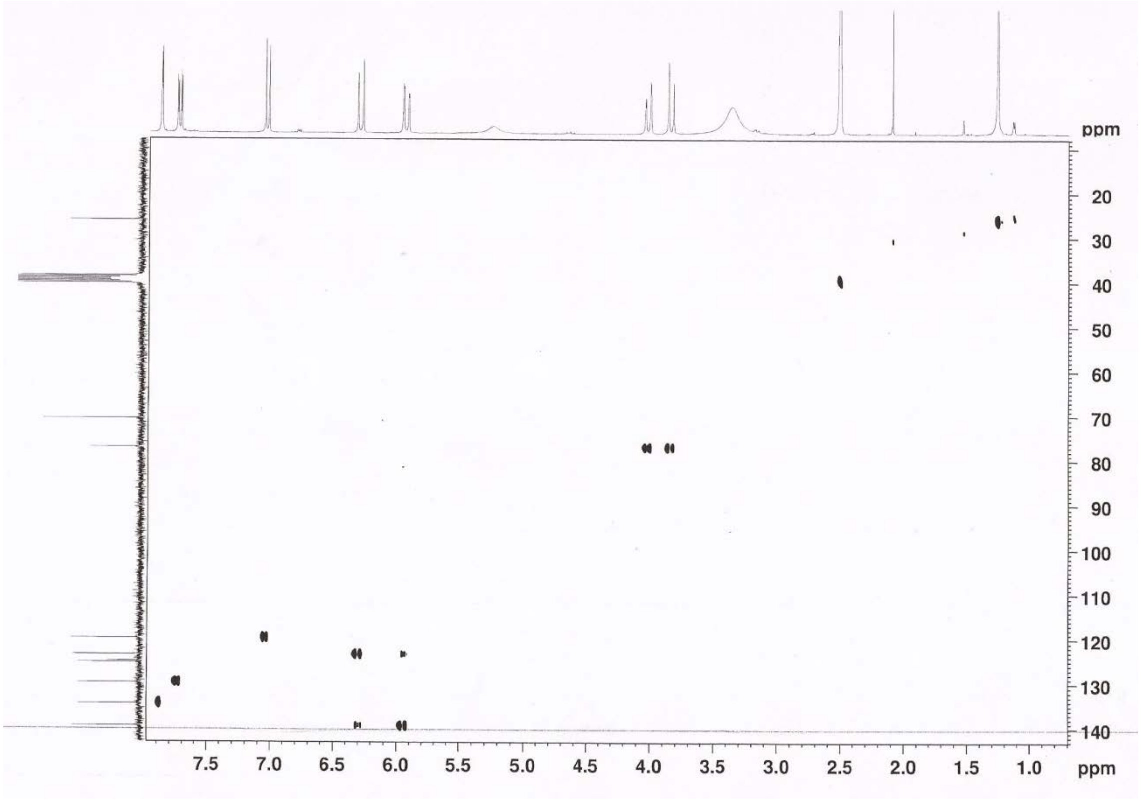


**Figure S22.** HSQC spectrum of compound **3** (DMSO, 300.13 MHz).

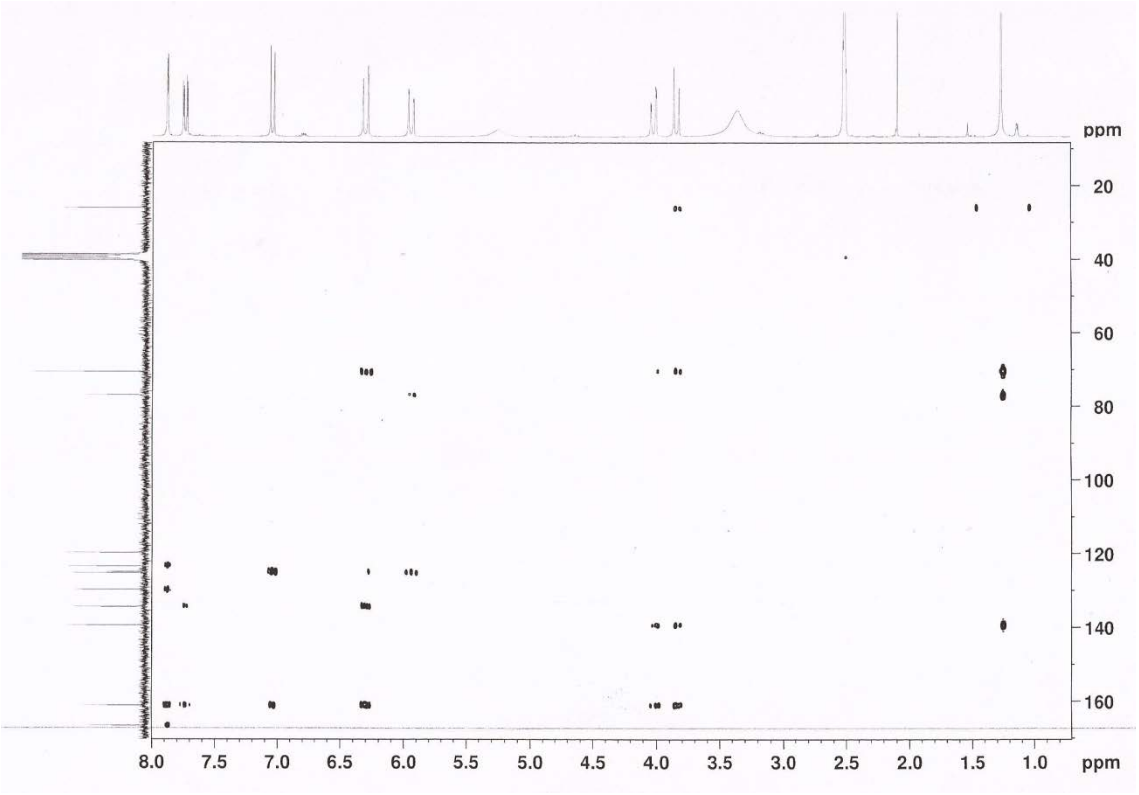


**Figure S23.** HMBC spectrum of compound **3** (DMSO, 300.13 MHz).

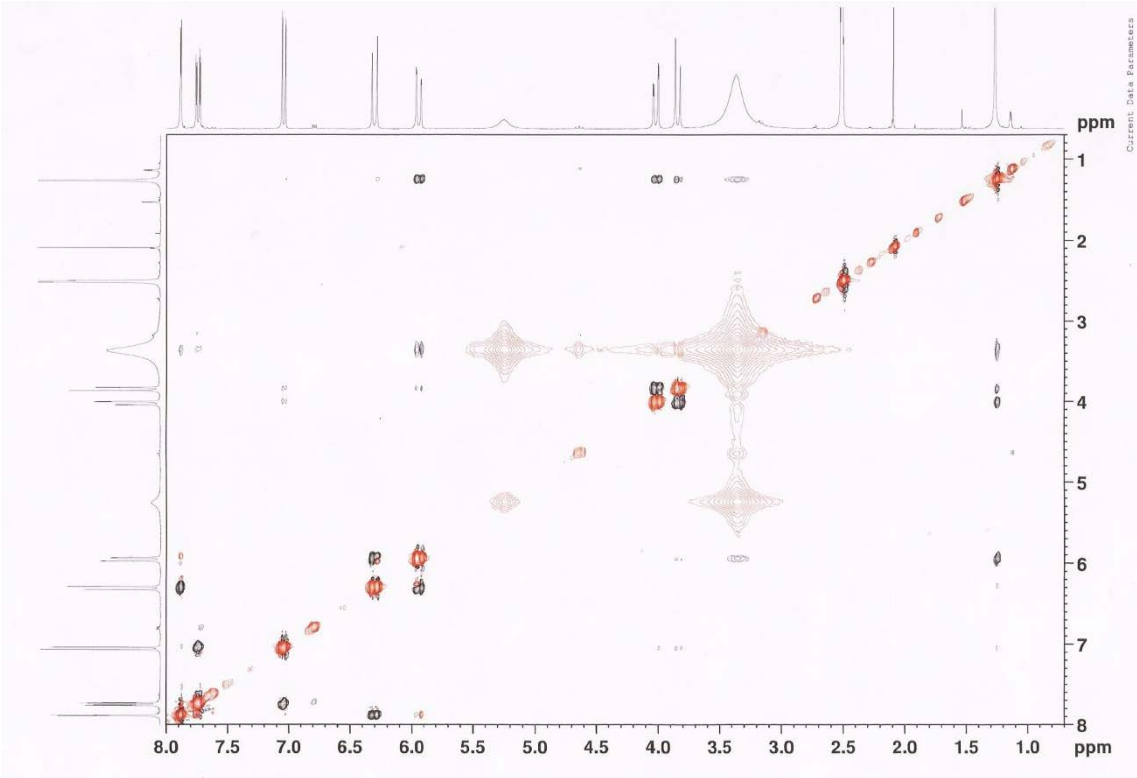


**Figure S24.** NOESY spectrum of compound **3** (DMSO, 300.13 MHz).

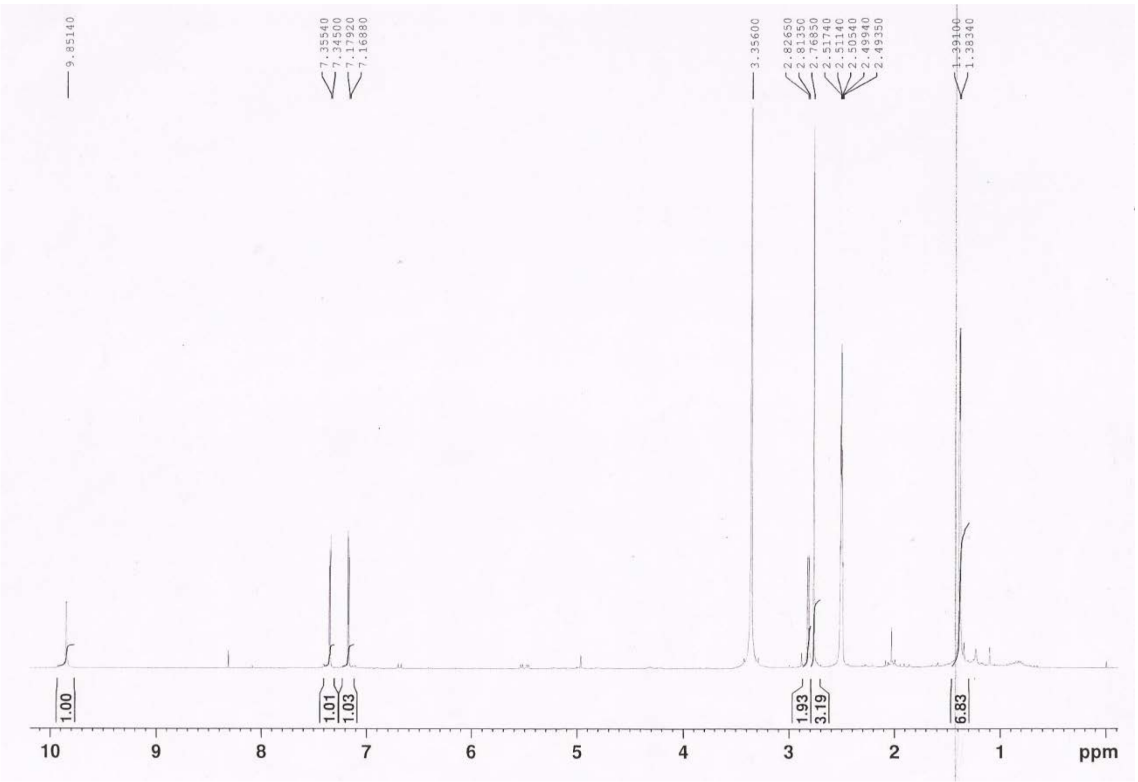


**Figure S25.** $^{1}$H NMR spectrum of compound **4b** (DMSO, 300.13 MHz).

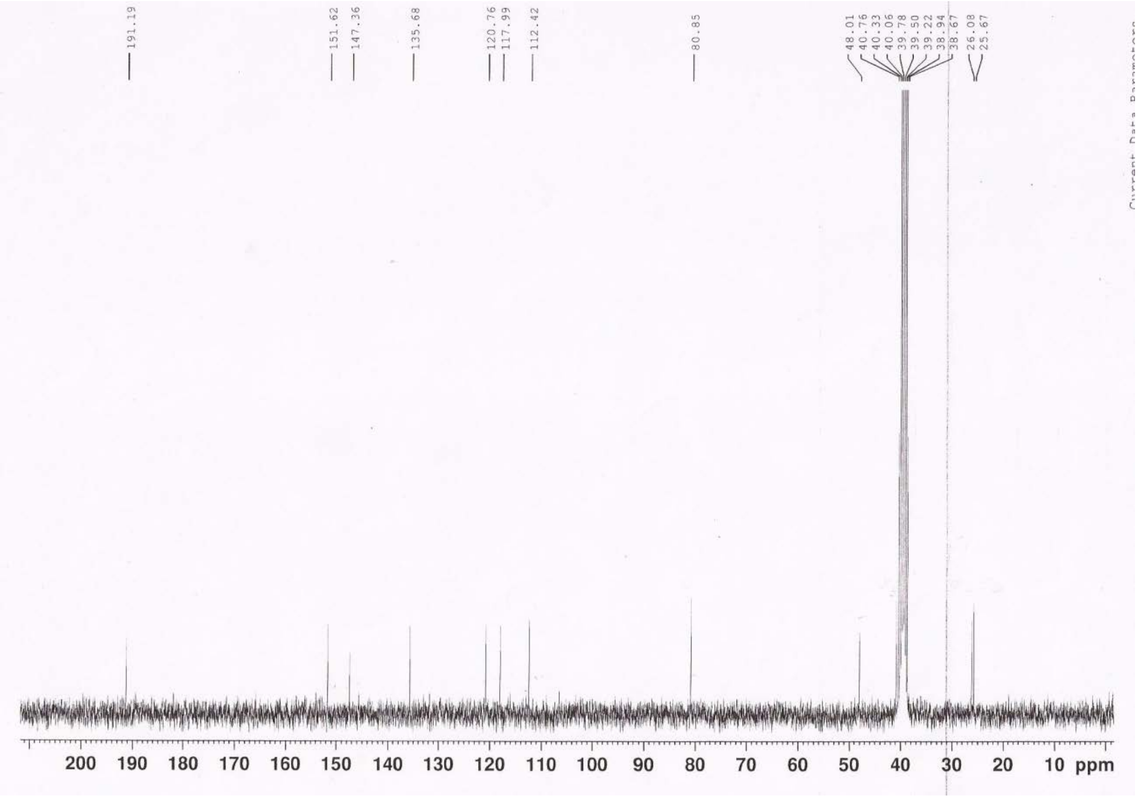


**Figure S26.** $^{13}C$ NMR spectrum of compound **4b** (DMSO, 75.4 MHz).

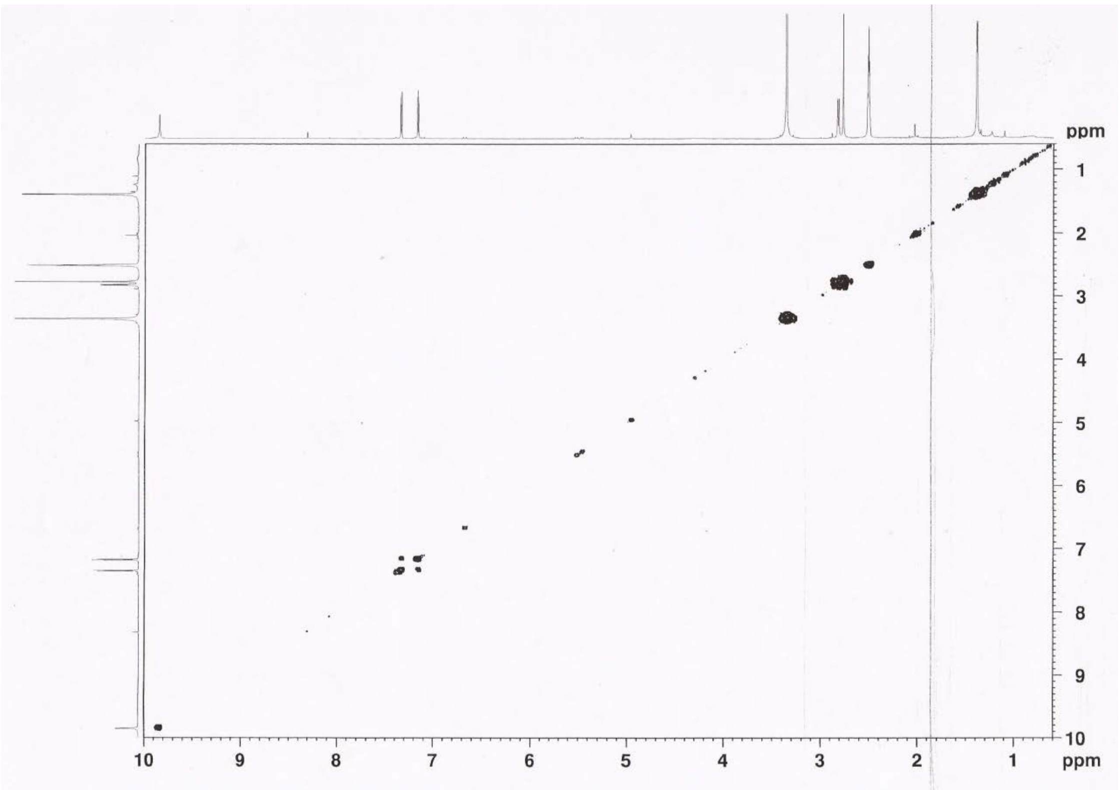


**Figure S27.** COSY spectrum of compound **4b** (DMSO, 300.13 MHz).

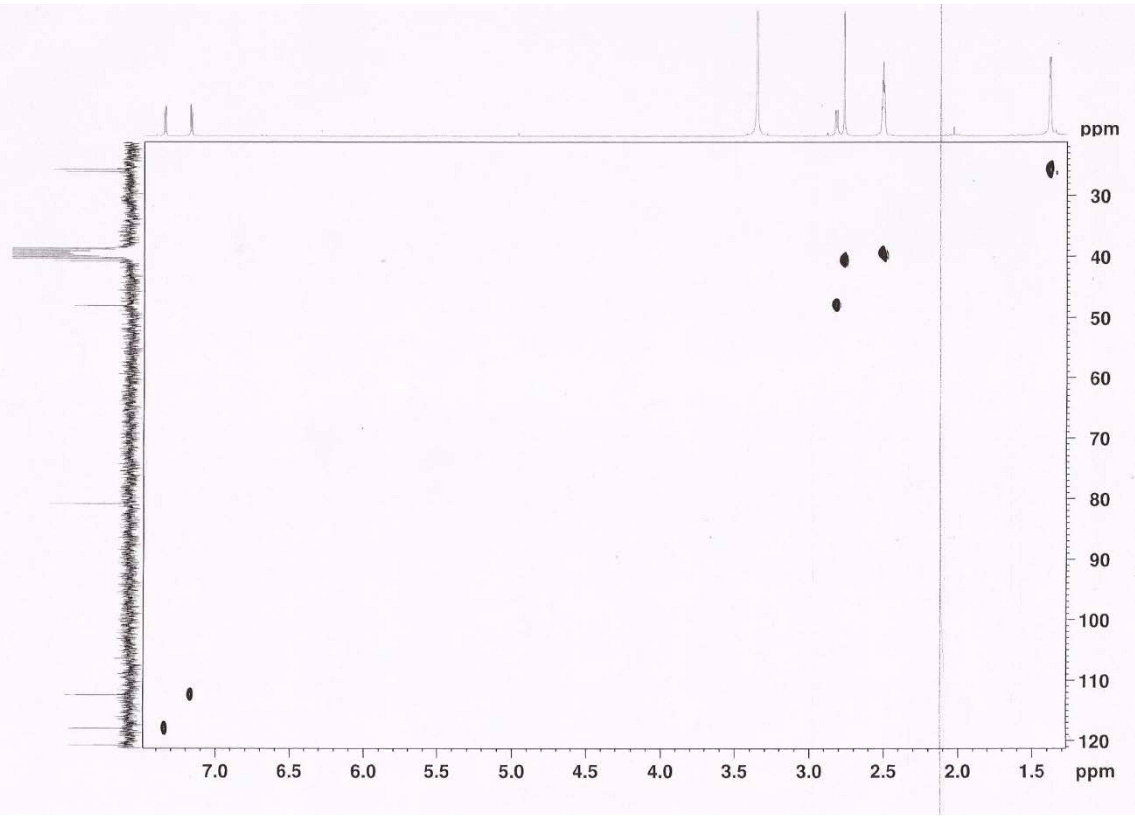


**Figure S28.** HSQC spectrum of compound **4b** (DMSO, 300.13 MHz).

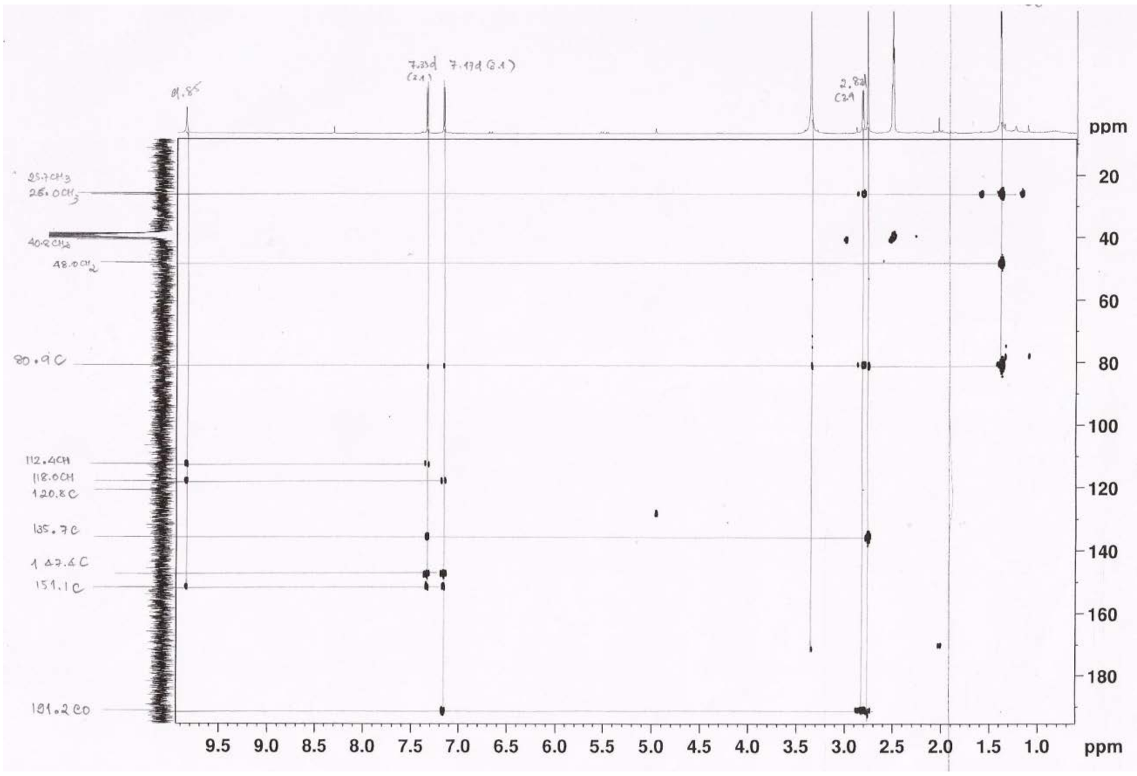


**Figure S29.** HMBC spectrum of compound **4b** (DMSO, 300.13 MHz).

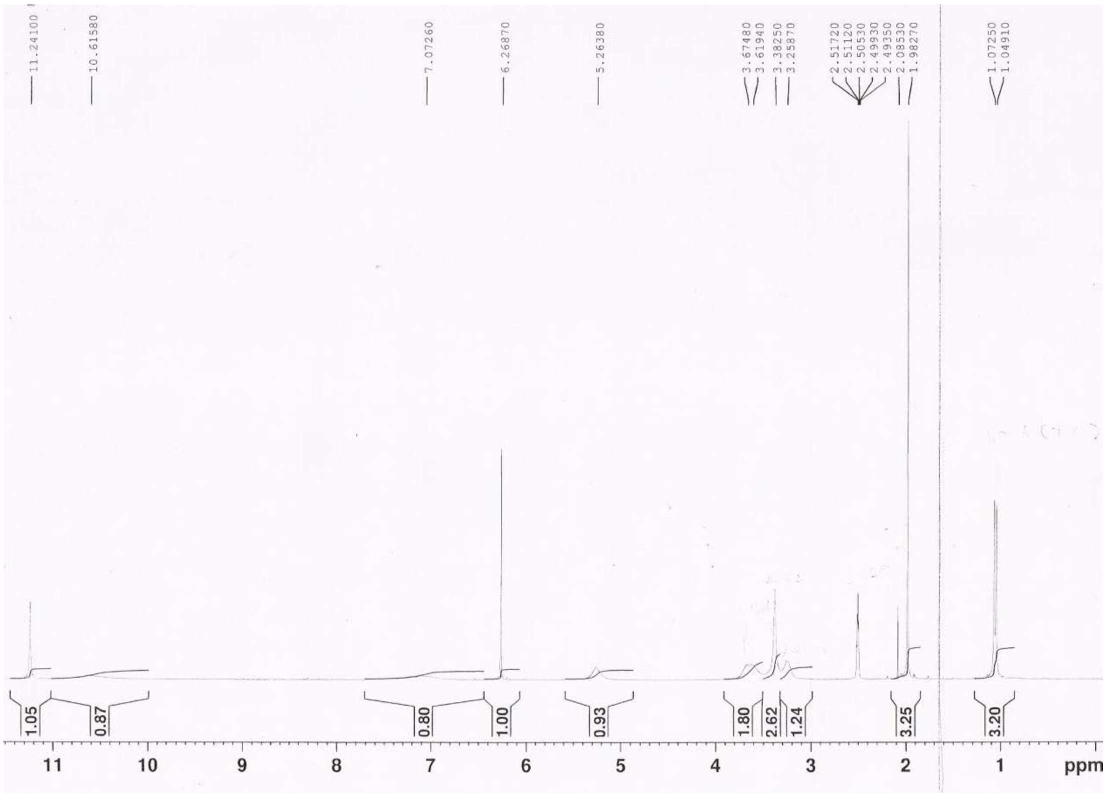


**Figure S30.** $^{1}H$ spectrum of compound **5** (DMSO, 300.13 MHz).

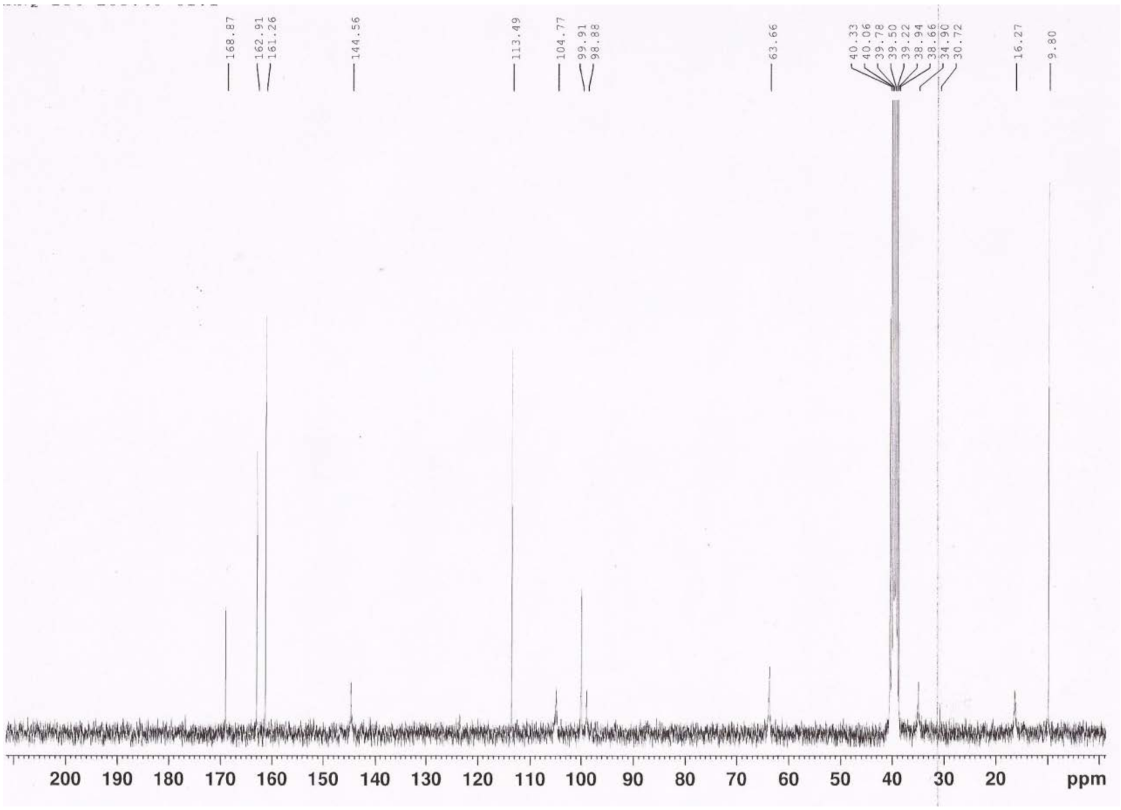


**Figure S31.** $^{13}C$ spectrum of compound **5** (DMSO, 75.4 MHz).

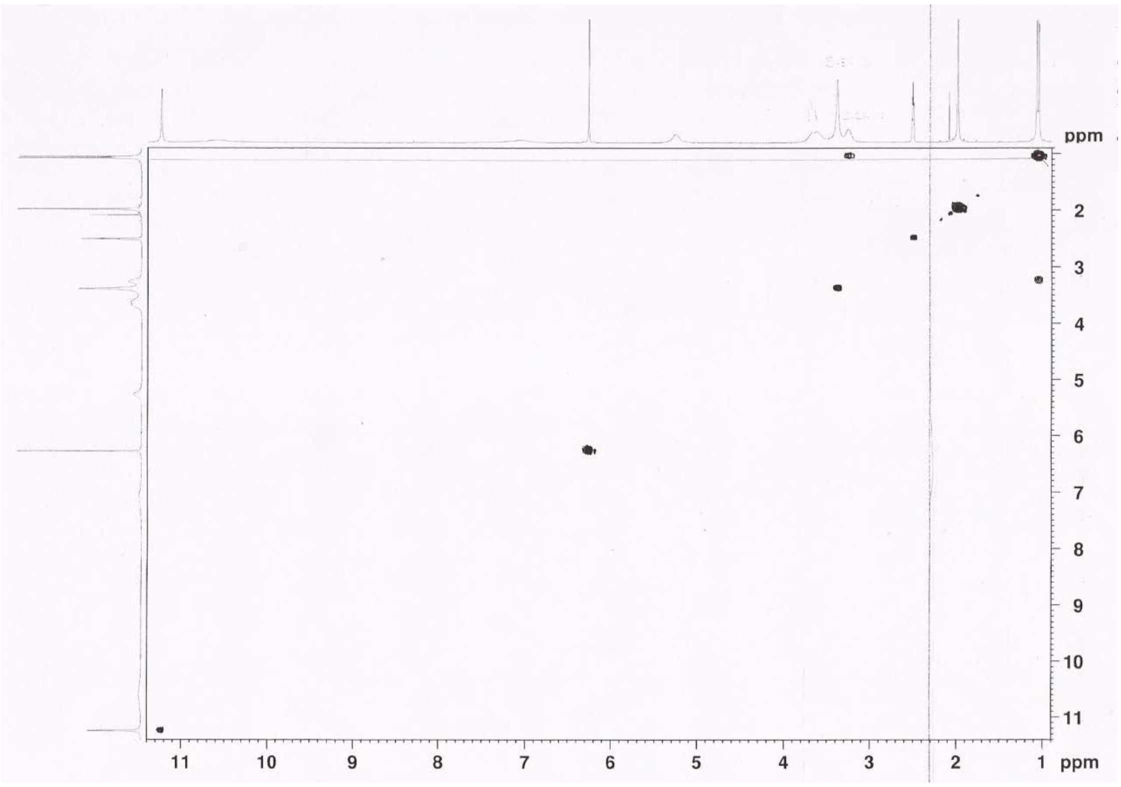


**Figure S32.** COSY spectrum of compound **5** (DMSO, 300.13 MHz).

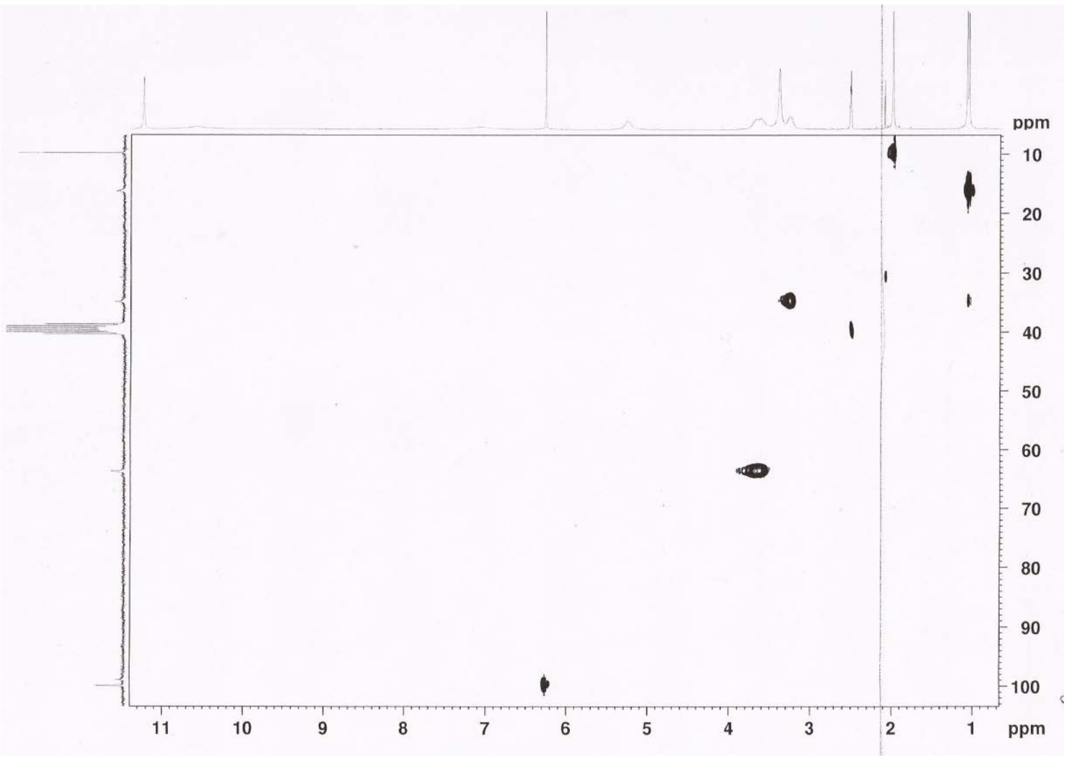


**Figure S33.** HSQC spectrum of compound **5** (DMSO, 300.13 MHz).

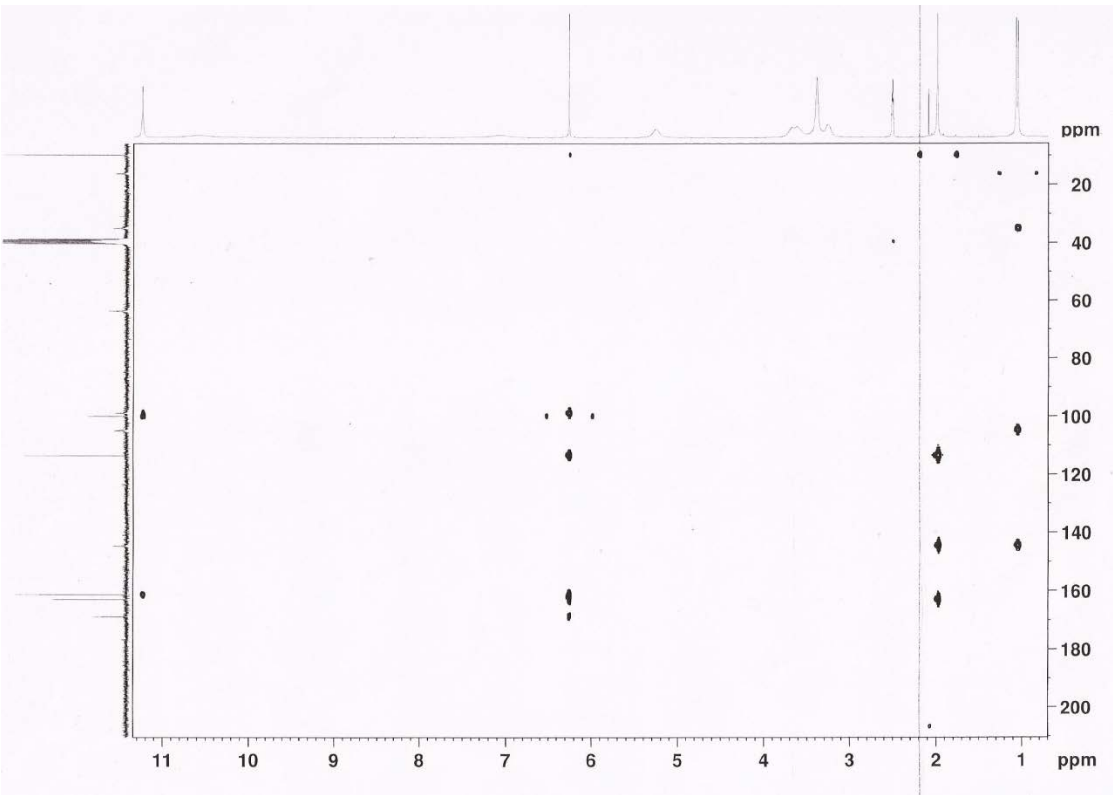


**Figure S34.** HMBC spectrum of compound **5** (DMSO, 300.13 MHz).

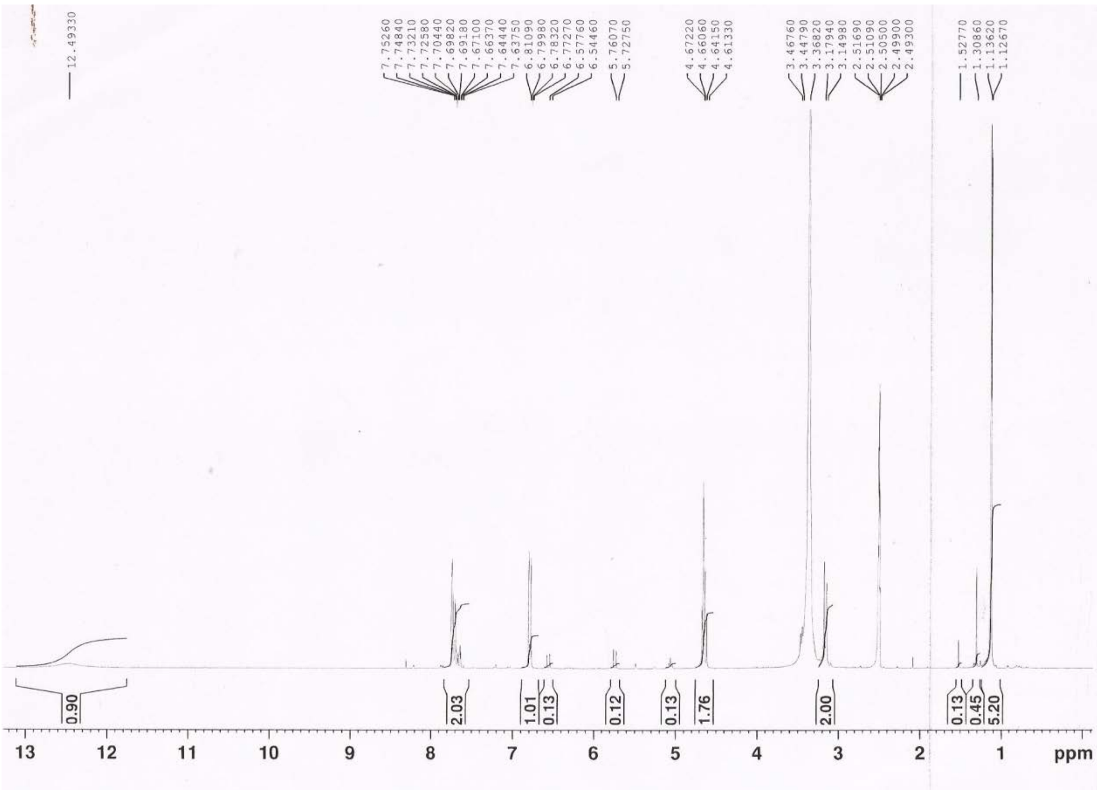


**Figure S35.** $^{1}$H spectrum of compound **6a** (DMSO, 300.13 MHz).

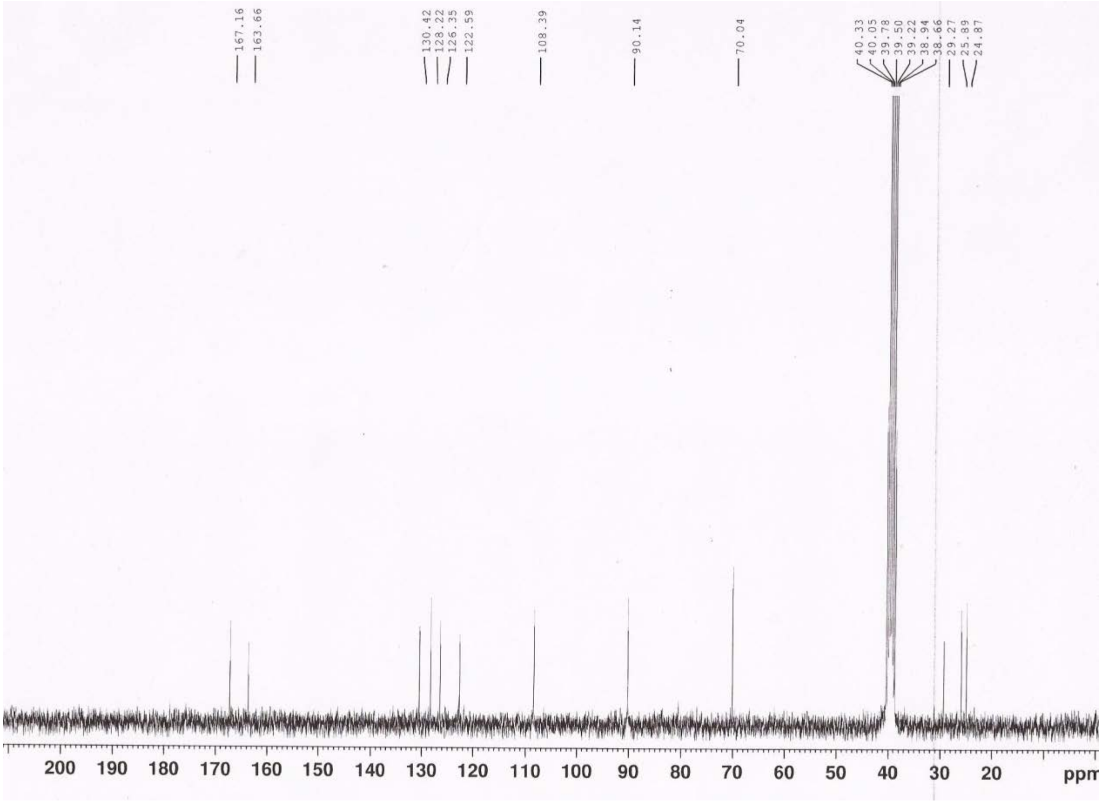


**Figure S36.** $^{13}C$ spectrum of compound **6a** (DMSO, 75.4 MHz).

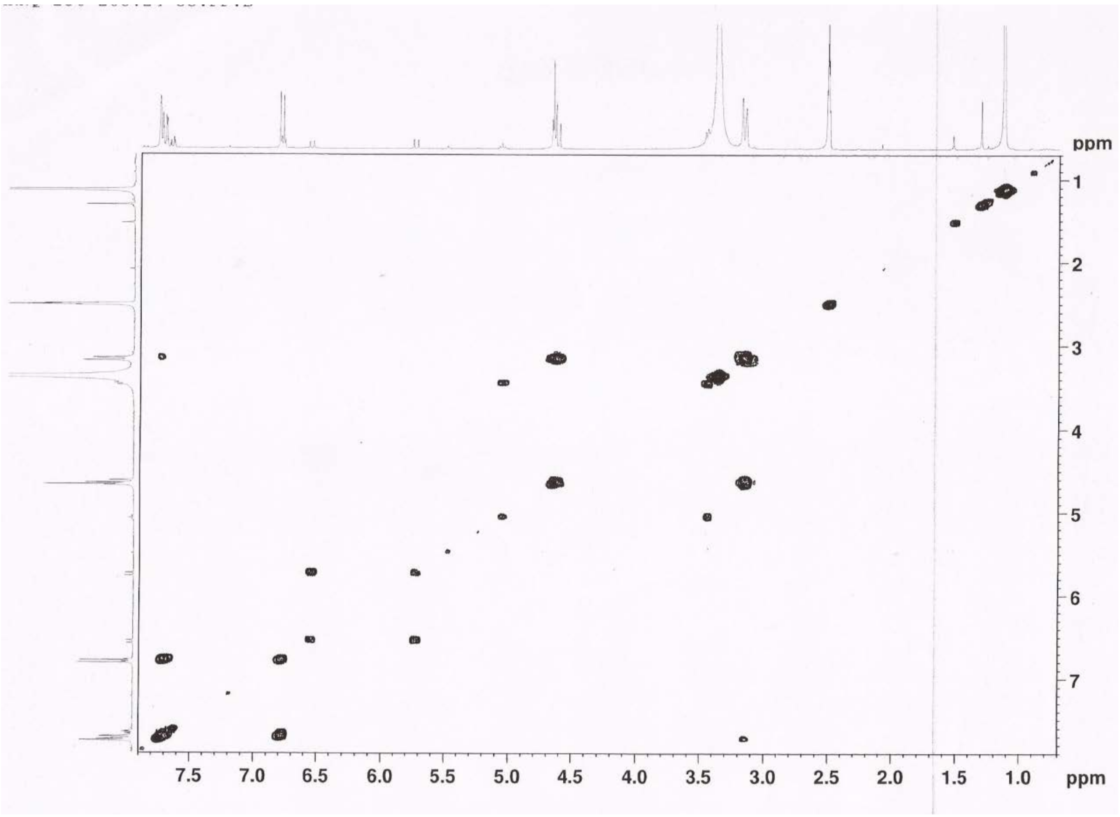


**Figure S37.** COSY spectrum of compound **6a** (DMSO, 300.13 MHz).

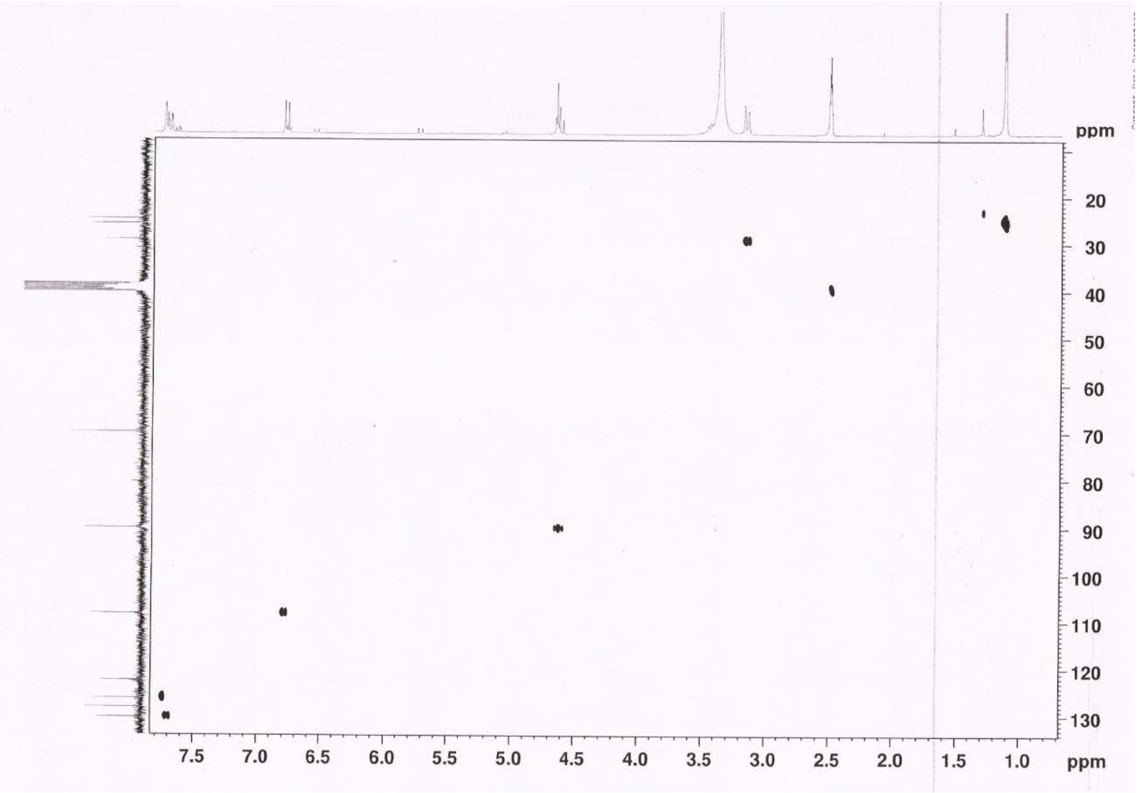


**Figure S38.** HSQC spectrum of compound **6a** (DMSO, 300.13 MHz).

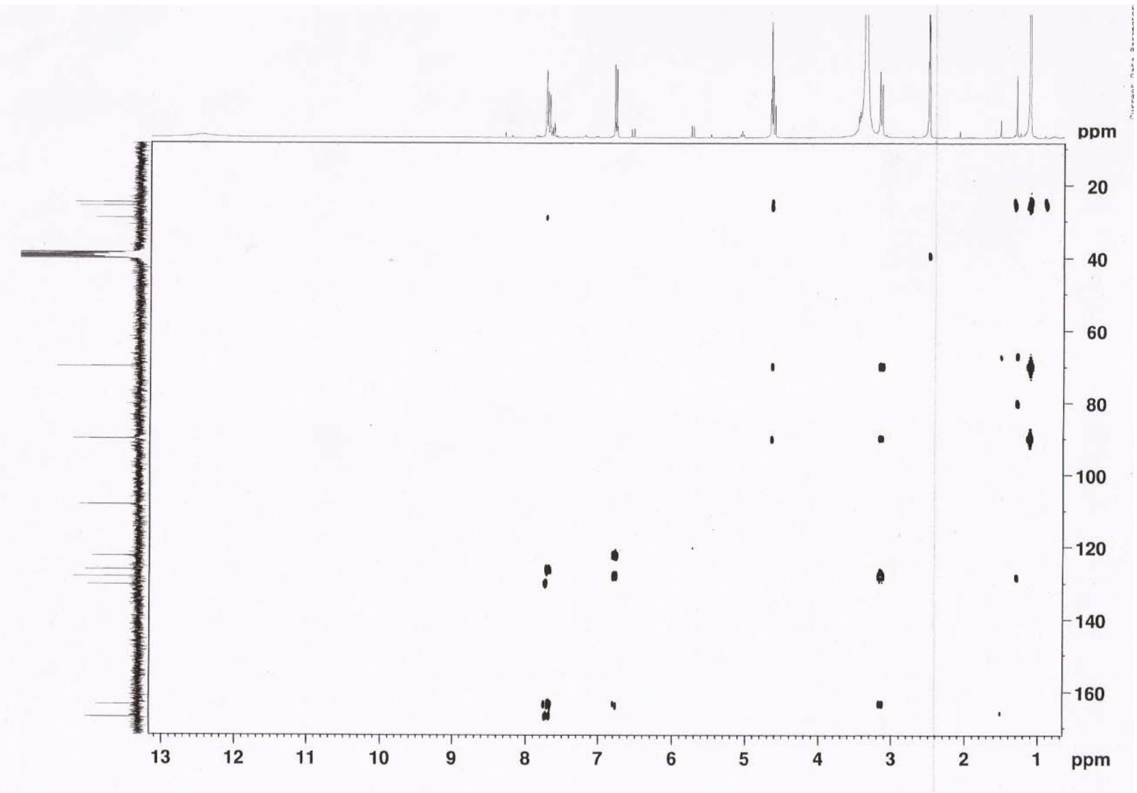


**Figure S39.** HMBC spectrum of compound **6a** (DMSO, 300.13 MHz).

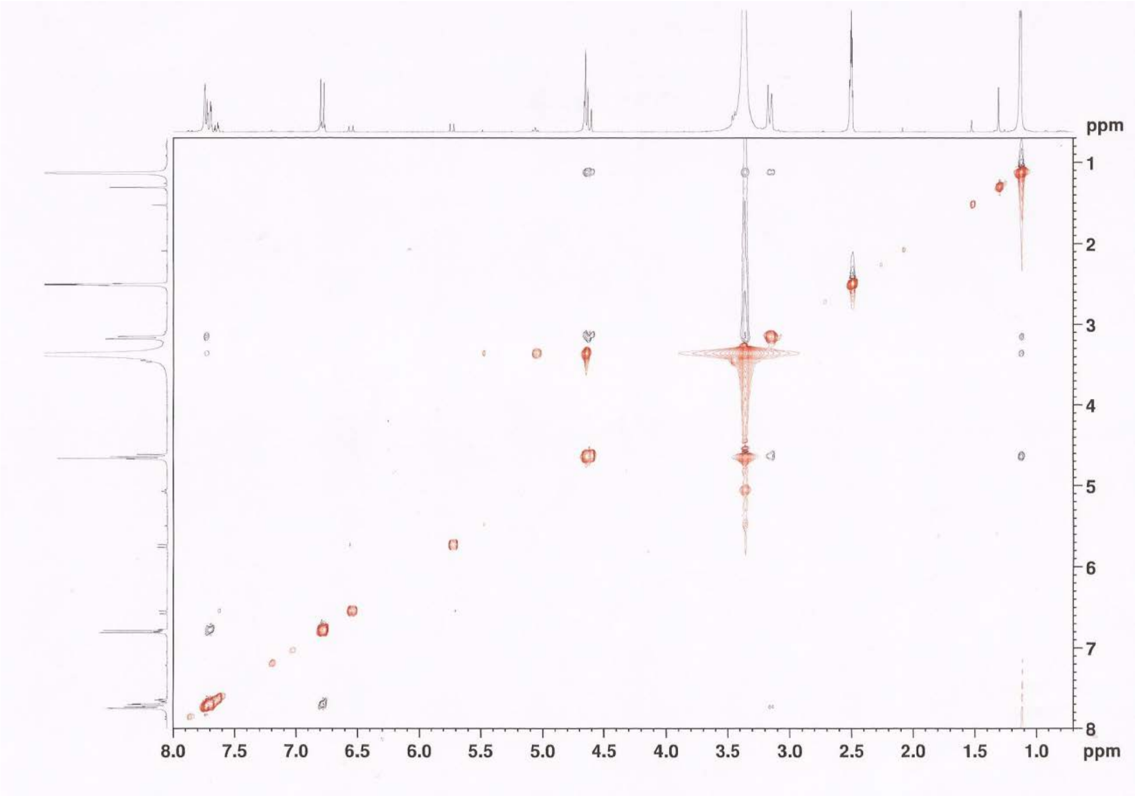


**Figure S40.** NOESY spectrum of compound **6a** (DMSO, 300.13 MHz).

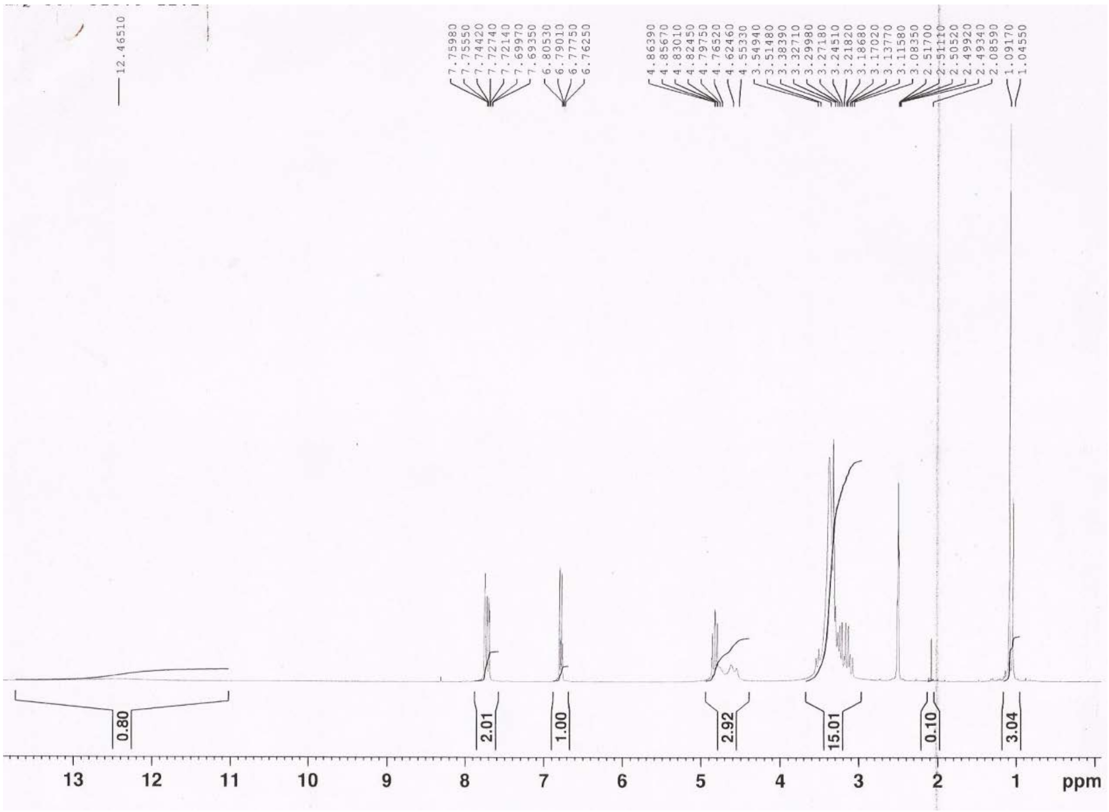


**Figure S41.** $^{1}$H spectrum of compound **6b** (DMSO, 300.13 MHz).

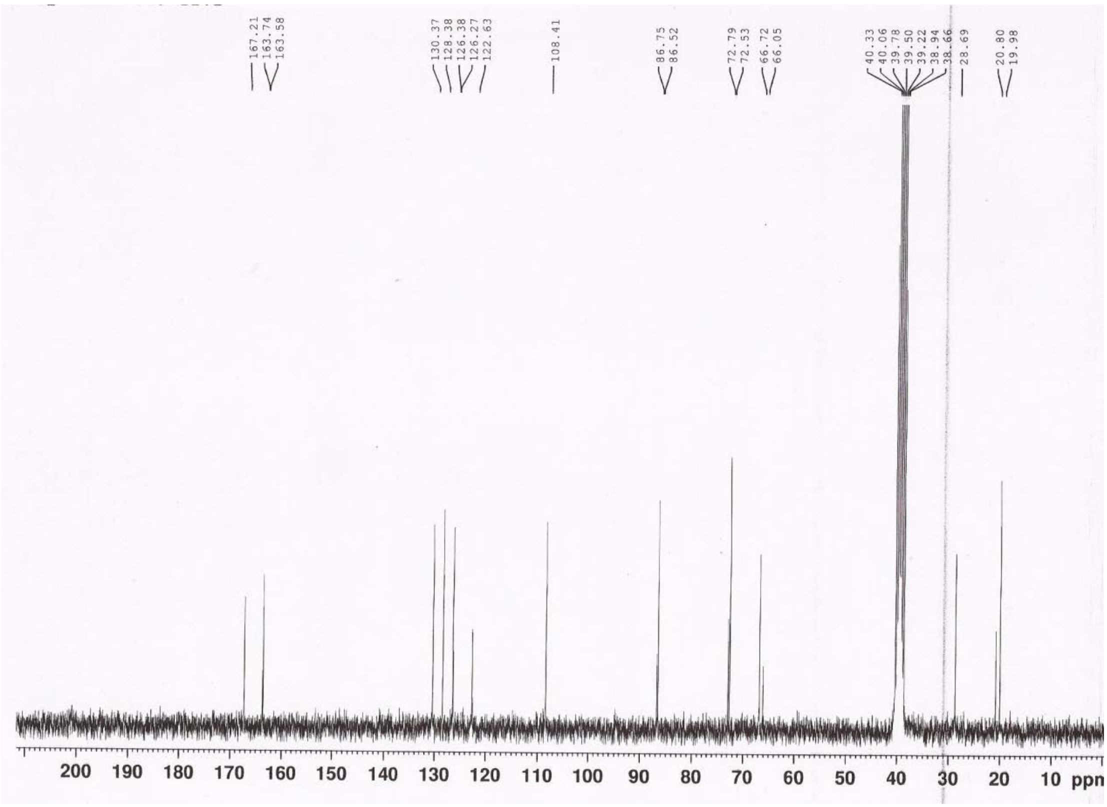


**Figure S42.** $^{13}C$ spectrum of compound **6b** (DMSO, 75.4 MHz).

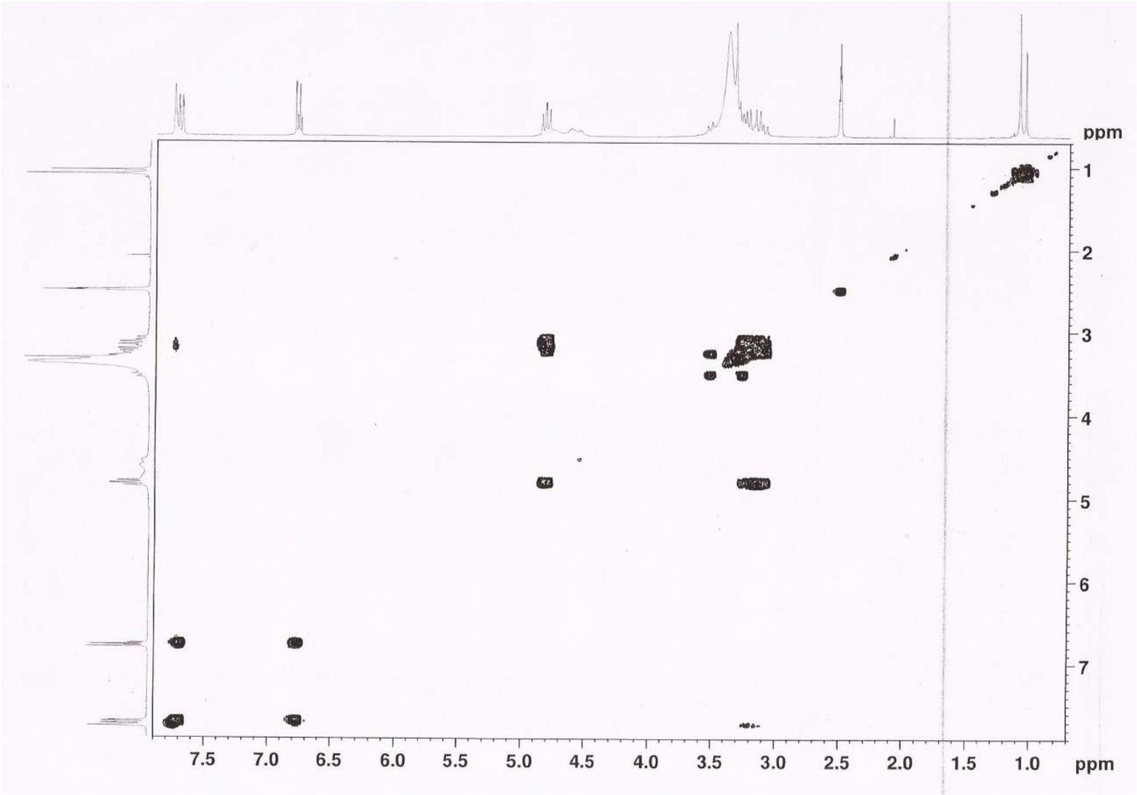


**Figure S43.** COSY spectrum of compound **6b** (DMSO, 300.13 MHz).

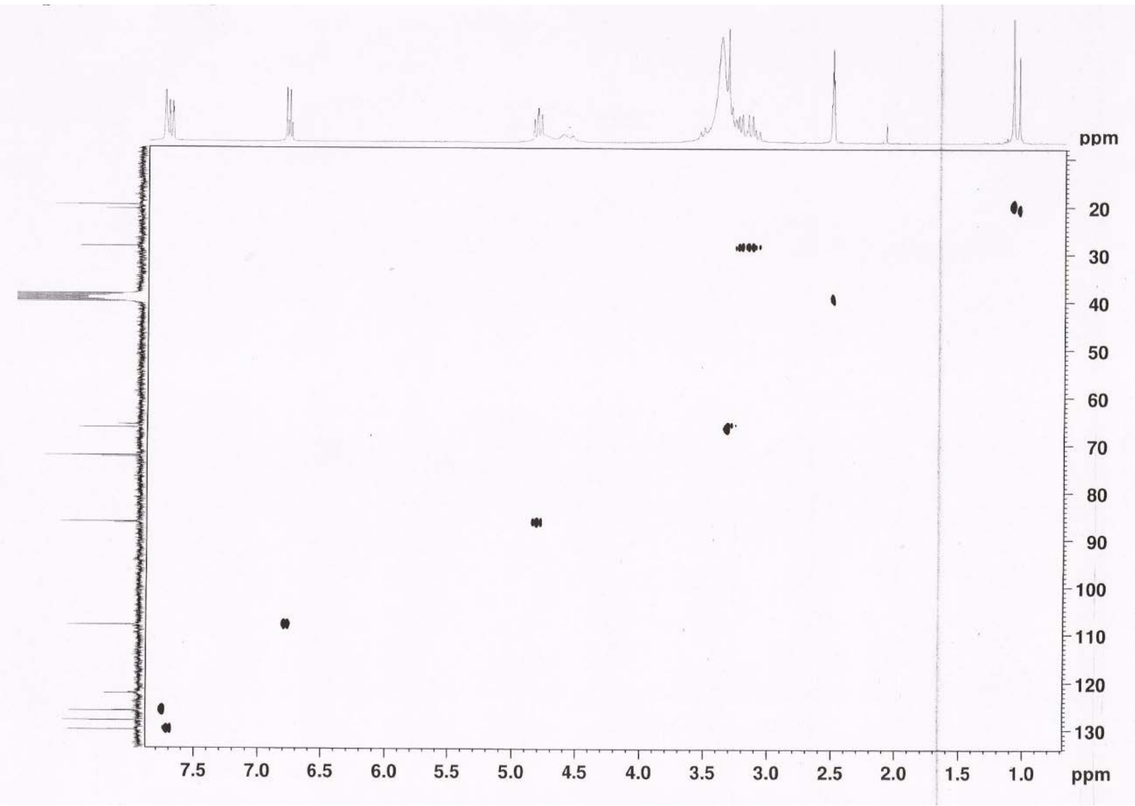


**Figure S44.** HSQC spectrum of compound **6b** (DMSO, 300.13 MHz).

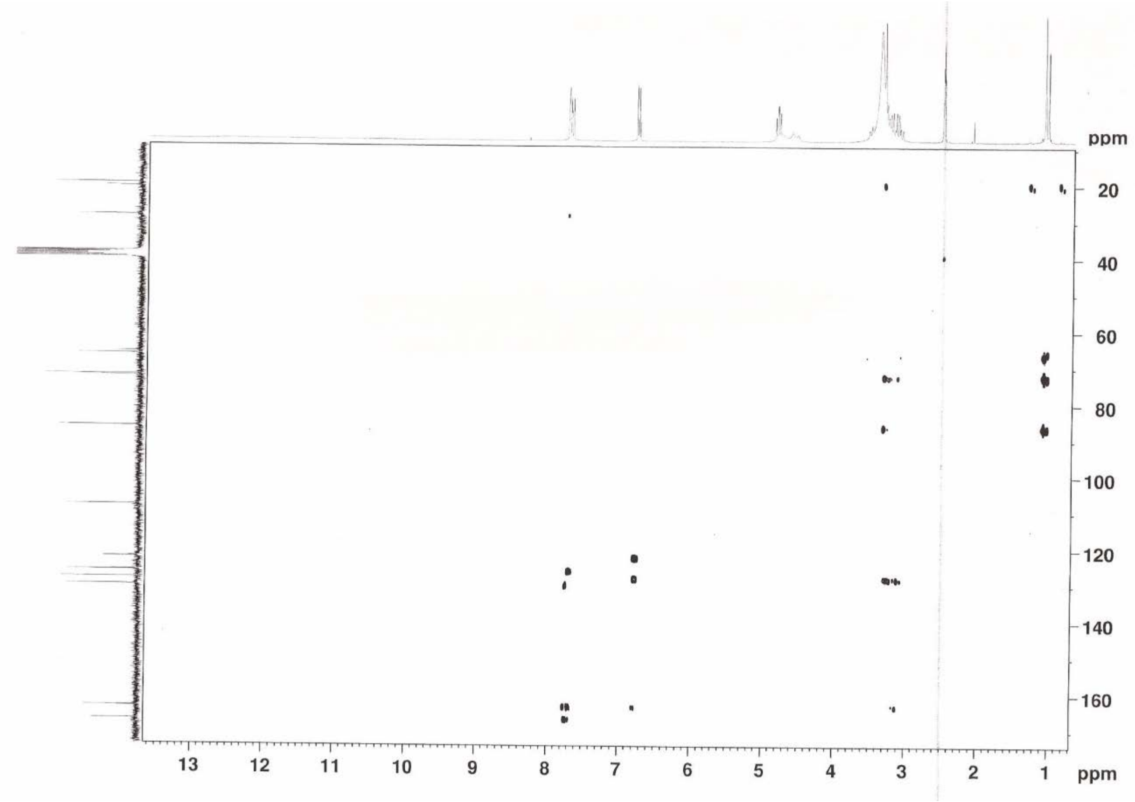


**Figure S45.** HMBC spectrum of compound **6b** (DMSO, 300.13 MHz).

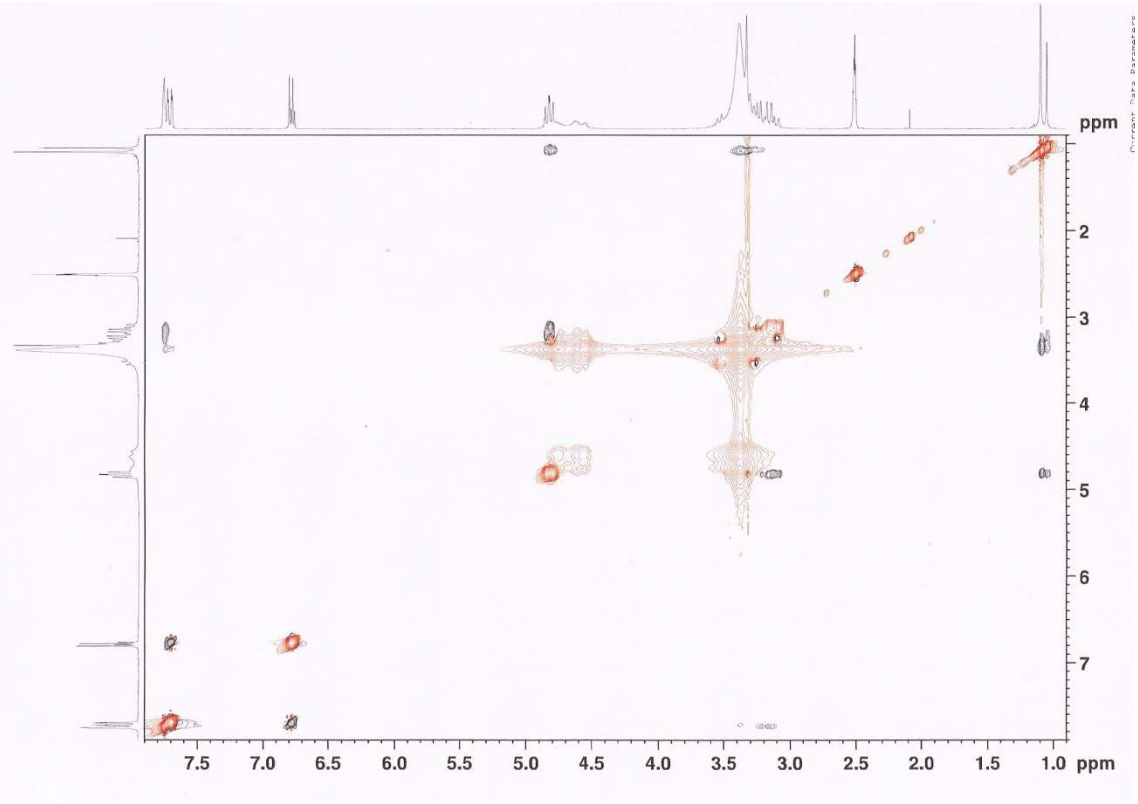


**Figure S46.** NOESY spectrum of compound **6b** (DMSO, 300.13 MHz).

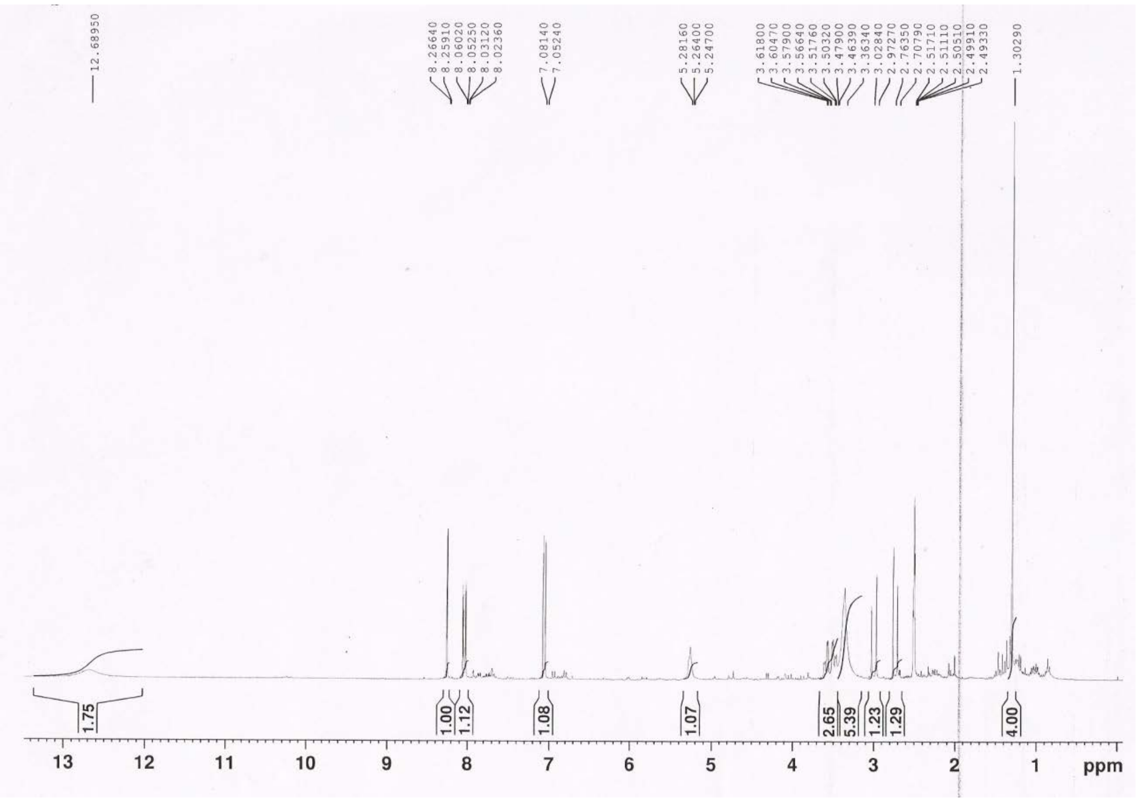


**Figure S47.** $^1$H NMR spectrum of compound **7** ($CDCl_3$, 300.13 MHz).

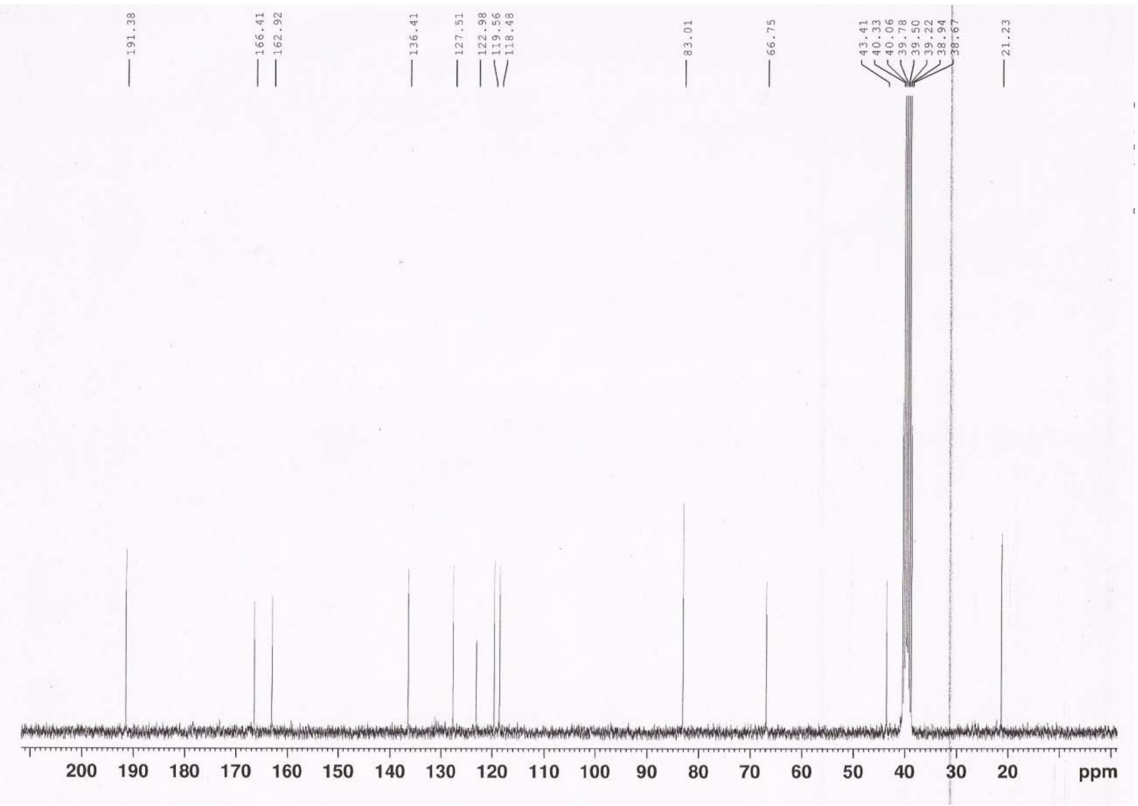


**Figure S48.** $^{13}$CNMR spectrum of compound **7** ($CDCl_3$, 75.4 MHz).

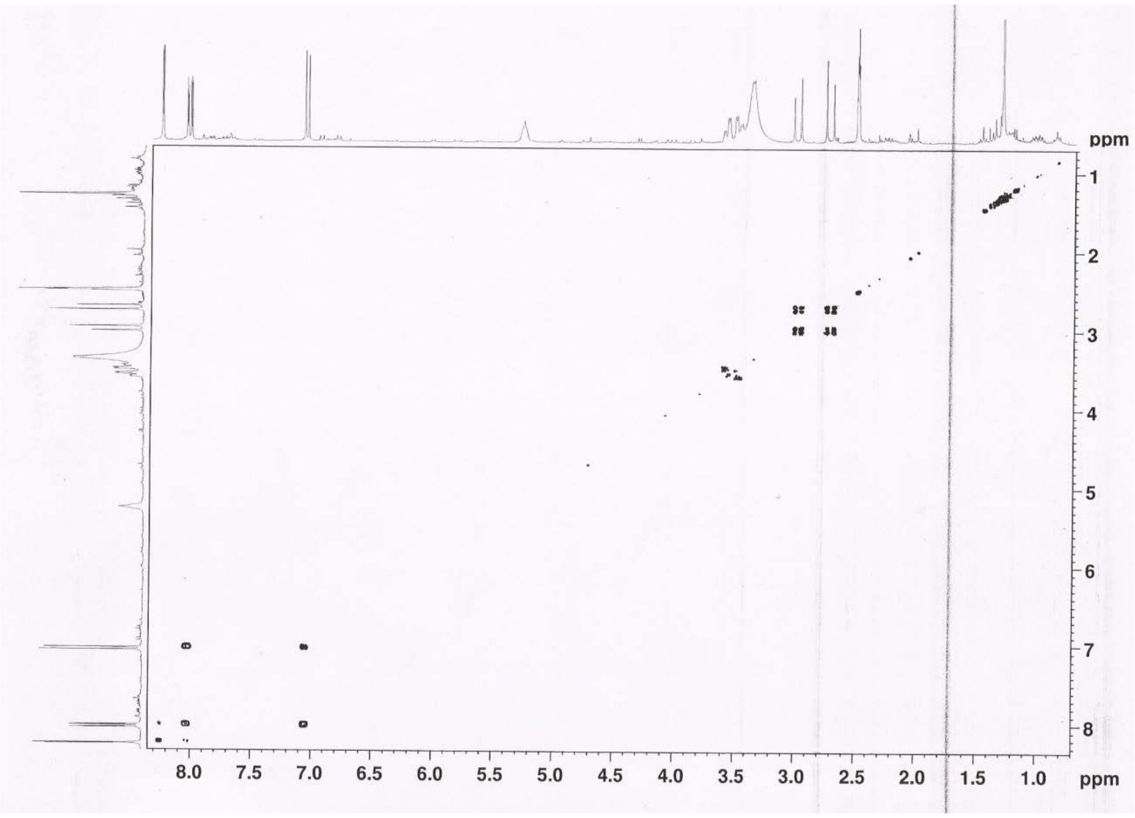


**Figure S49.** COSY spectrum of compound **7** ($CDCl_3$, 75.4 MHz).

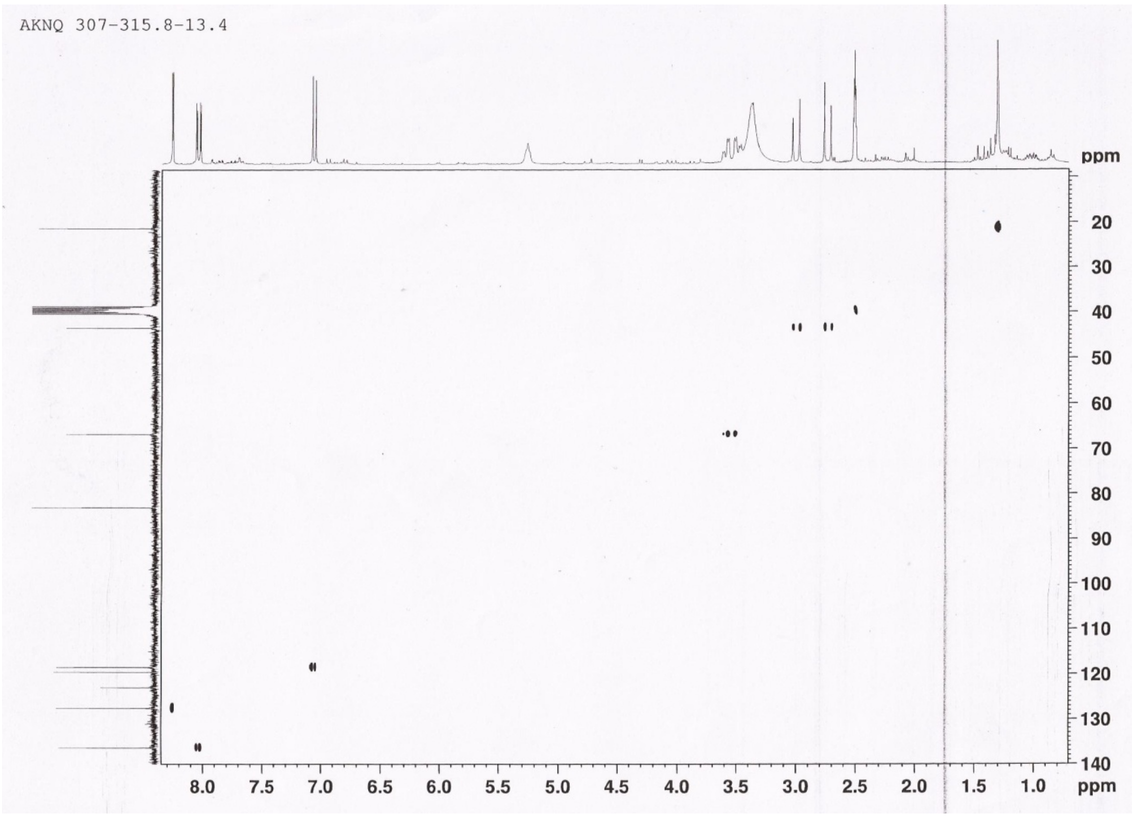


**Figure S50.** HSQC spectrum of compound **7** ($CDCl_3$, 75.4 MHz).

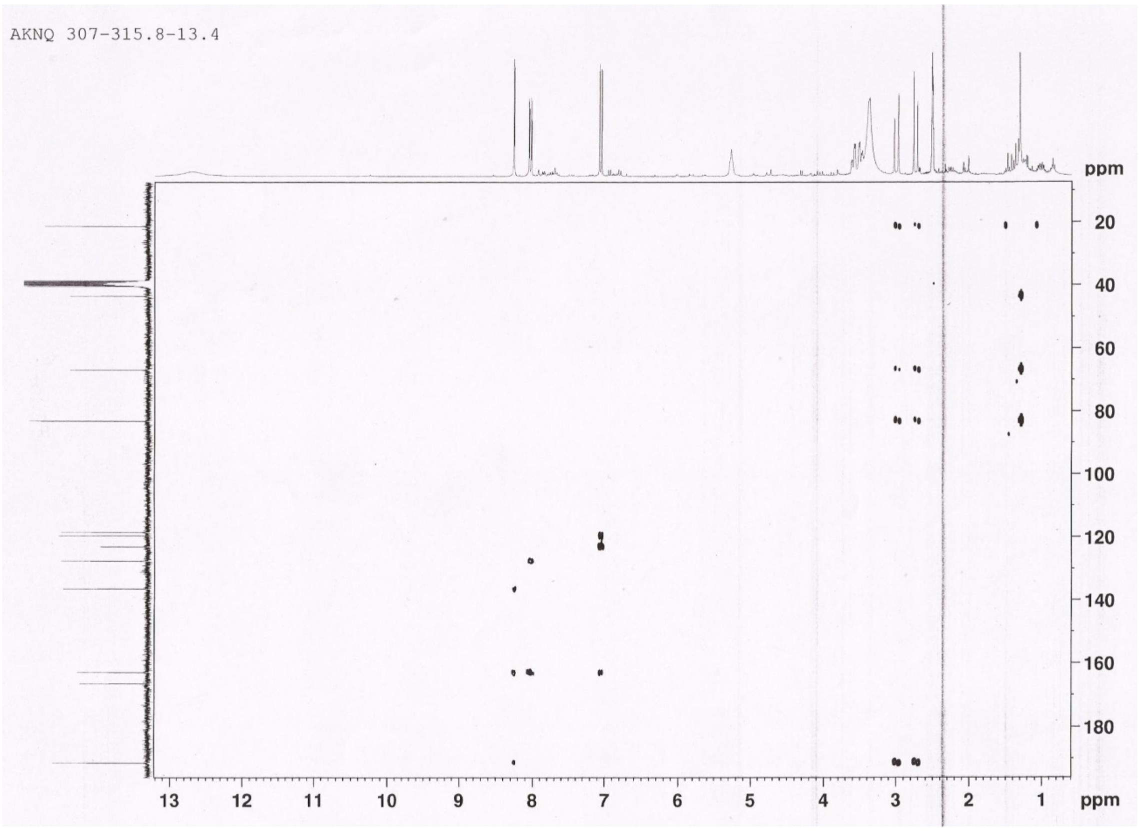


**Figure 51S.** HMBC spectrum of compound **7** ($CDCl_3$, 75.4 MHz).

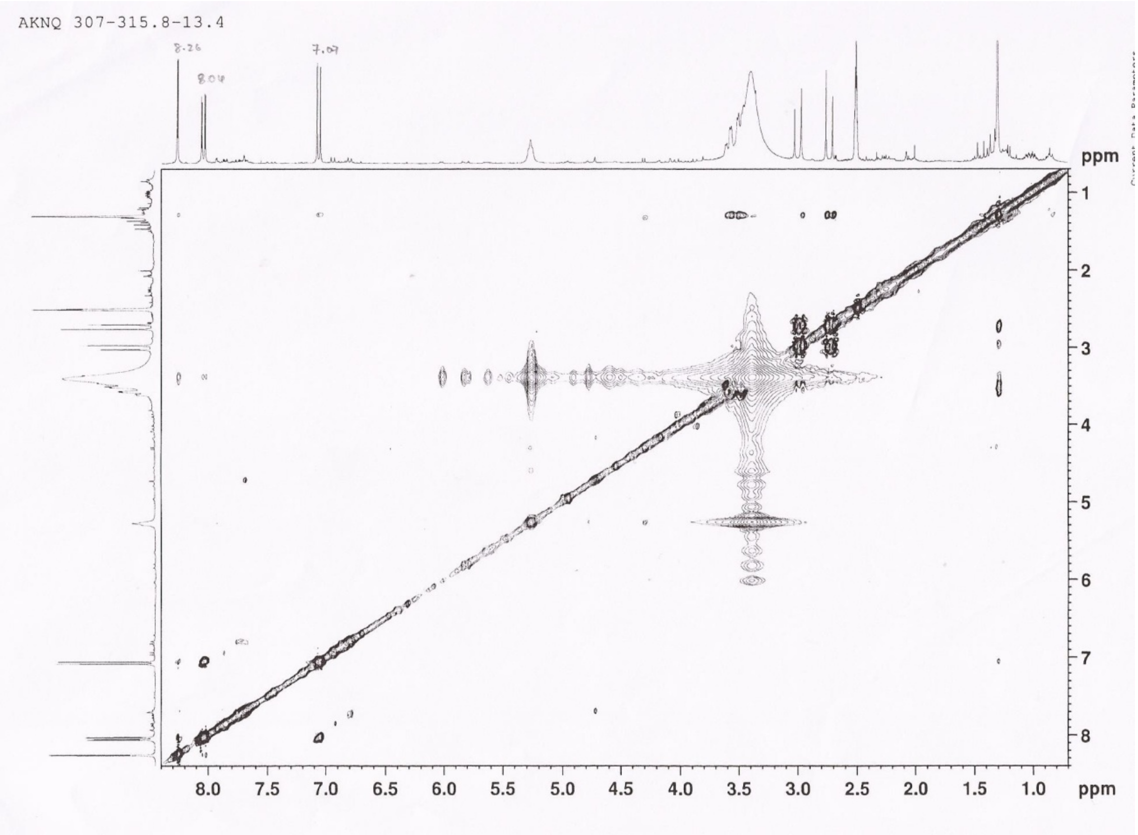


**Figure S52.** NOESY spectrum of compound **7** ($CDCl_3$, 75.4 MHz).
